# A high-coverage Neandertal genome from the Altai Mountains reveals population structure among Neandertals

**DOI:** 10.1073/pnas.2534576123

**Published:** 2026-03-23

**Authors:** Diyendo Massilani, Stéphane Peyrégne, Leonardo N. M. Iasi, Cesare de Filippo, Fabrizio Mafessoni, Alba Bossoms Mesa, Arev P. Sümer, Yaniv Swiel, Divyaratan Popli, Shahar Silverman, Michael James Boyle, Maxim B. Kozlikin, Michael V. Shunkov, Anatoly P. Derevianko, Tom Higham, Katerina Douka, Matthias Meyer, Hugo Zeberg, Janet Kelso, Svante Pääbo

**Affiliations:** ^a^Department of Genetics, Yale School of Medicine, New Haven, CT 06510; ^b^Department of Evolutionary Genetics, Max Planck Institute for Evolutionary Anthropology, Leipzig 04103, Germany; ^c^Department of Life Sciences, University of Trieste, Trieste 34127, Italy; ^d^Institute of Archaeology and Ethnography of the Siberian Branch of the Russian Academy of Sciences, Novosibirsk 630090, Russia; ^e^Department of Evolutionary Anthropology, Faculty of Life Sciences, University of Vienna, Vienna 1030, Austria; ^f^Human Evolution and Archaeological Sciences Network, University of Vienna, Vienna 1030, Austria

**Keywords:** Neandertal genome, Denisovans, archaic hominins

## Abstract

We present a genome sequenced to ~37-fold genomic coverage from an approximately 110,000-y-old male Neandertal from Denisova Cave in the Altai Mountains and analyze it together with previously published Neandertal genomes of high quality. We show that he belonged to a population more closely related to a ~120,000-y-old Neandertal from Denisova Cave than to Neandertals in Europe or to a ~80,000-y-old Neandertal from Chagyrskaya Cave in the Altai Mountains. Both Neandertals from Denisova Cave show evidence of gene flow from Denisovans, a pattern not seen in later Neandertals from the Altai region or from Western Europe. The extent of chromosomal regions of homozygosity in Neandertals from the Altai region between 120,000 and 80,000 y ago indicates that they lived in smaller and more isolated groups than later Neandertals in Europe (54,000 to 40,000 y ago). We estimate the extent of allele frequency differentiation among Neandertal populations and find that the older Eastern Neandertals in the Altai region and younger Western Neandertals in Europe were as differentiated as the most differentiated present-day human populations worldwide.

## Significance

We present a high-quality genome of a ~110,000-y-old male Neandertal from Denisova Cave in the Altai Mountains. He as well as a ~120,000-y-old Neandertal from the same cave lived in smaller and more isolated groups than later Neandertals in Europe and the ancestors of both individuals mixed with Denisovans. The older Eastern and younger Western Neandertals were as differentiated in terms of the frequencies of genetic variants as the most differentiated present-day human populations worldwide, suggesting that present-day humans exhibit relatively low levels of population differentiation compared to Neandertals.

**Fig. 1.**
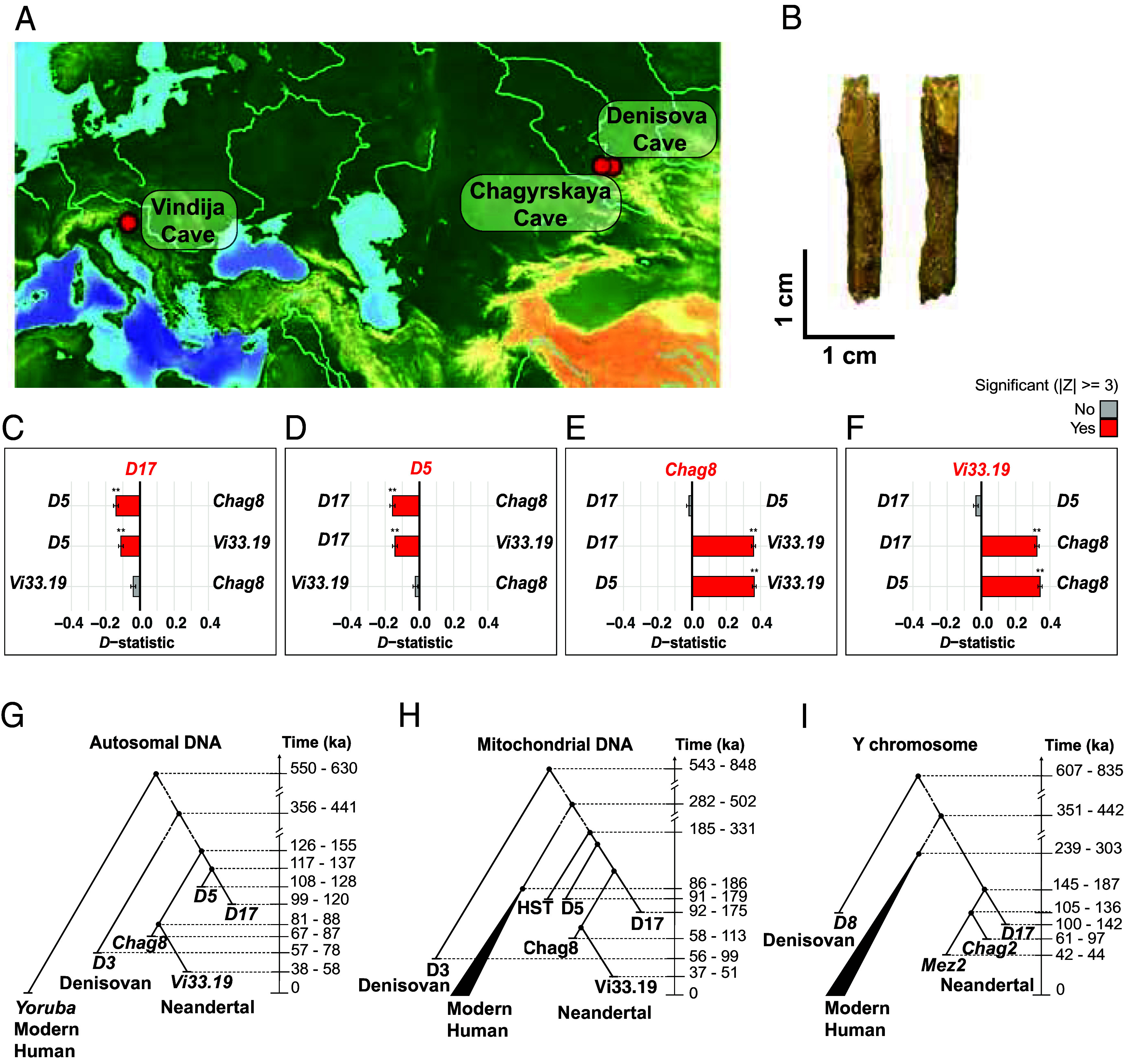
Neandertal *D17* and its relationship with other Neandertals. (*A*) Locations of high-coverage Neandertal genomes used in the study. (*B*) Picture of the undiagnosed bone fragment from Neandertal *D17*. (*C*–*F*) Relative derived allele sharing between Neandertal genomes, computed using *D-*statistics of the form *D-(ind1, ind2; ind3, Mbuti)*, where *ind3* is either (*C*) Neandertal *D17*, (*D*) Neandertal *D5*, (*E*) Neandertal *Chag8* or (*F*) Neandertal *Vi33.19*. In each panel, ind1 and ind2 are indicated at the *Left* and *Right* of the graph. Positive values indicate greater allele sharing between *ind3* and *ind1*; negative values indicate greater allele sharing between *ind3* and *ind2*. |Z-score| ≥ 3 are in red. (*G*) Schematic phylogenetic relationships among Neandertal *D17,* other archaic genomes, including the Denisovan *D3*, and modern humans inferred from autosomal DNA analyses using branch shortening and demographic modeling with *cecast* and F(A|B) statistics (*SI Appendix*, *SI Appendix* 6, 10, and 12). (*H*) Schematic mitochondrial (mt) DNA inferred from a Bayesian tree estimated using BEAST2 from previously published study on *D17* mtDNA (11). (*I*) Y chromosome phylogeny as inferred from a Bayesian tree estimated using BEAST2 including previously published Y chromosomes of the Denisovan *Denisova 8* (*D8*) and Neandertals *Mezmaiskaya 2* (*Mez2*) (12) and *Chagyrskaya 2* (*Chag2*) (7) (*SI Appendix*, *SI Appendix* 18).

Although genomic information from over 30 Neandertals has been generated to date (1–10), genomes of high genomic coverage have been published for only three Neandertal individuals: a ~120,000-y-old female from Denisova Cave, Altai, Siberia (*Denisova 5*, also called the “Altai Neandertal”) (1); a ~54,000-y-old female from Vindija Cave, Croatia (*Vindija 33.19*) (2); and an ~80,000-y-old female from Chagyrskaya Cave, Altai, Siberia (*Chagyrskaya 8*) (3) (Fig. 1*A* and Table 1). The two younger individuals are more closely related to one another and to the Neandertals who interacted and admixed with modern humans (2) than to the older Neandertal from Denisova Cave.

**Table 1.** Ancient human genomes used in this study

Individual	Abbreviation	Lineage	Site	Location	Age	Sex	Coverage	Reference
*Denisova 5* “Altai Neandertal”	*D5*	Neandertal	Denisova Cave	Altai Mountains, southern Siberia (Russia)	118 ka ± 10 ka (2 SD)	F	52-fold	Prüfer et al. (1)
*Vindija 33.19*	*Vi33.19*	Neandertal	Vindija Cave	Central Europe (Croatia)	49 ka ± 10 ka (2 SD)	F	30-fold	Prüfer et al. (2)
*Chagyrskaya 8*	*Chag8*	Neandertal	Chagyrskaya Cave	Altai Mountains, Siberia (Russia)	77 ka ± 10 ka (2 SD)	F	27-fold	Mafessoni et al. (3)
*Denisova 17*	*D17*	Neandertal	Denisova Cave	Altai Mountains, Siberia (Russia)	110 ka ± 10 ka (2 SD)	M	37-fold	This study
*Goyet 1*	*GN1*	Neandertal	Goyet Cave	Western Europe (Belgium)	N/A	F	22-fold	ENA accession PRJEB98484
*Denisova 3*	*D3*	Denisovan	Denisova Cave	Altai Mountains, Siberia (Russia)	65 ka ± 10 ka (2 SD)	F	31-fold	Meyer et al. (13)
*Denisova 25*	*D25*	Denisovan	Denisova Cave	Altai Mountains, Siberia (Russia)	205 ka ± 10 ka (2 SD)	M	24-fold	Peyrégne et al. (14)
*Ust’Ishim*	N/A	Modern Human	N/A	Western Siberia (Russia)	40 ka ± 9 ka (2 SD)	M	42-fold	Fu et al. (15)
*Loschbour*	N/A	Modern Human	Loschbourg rock shelter	Western Europe (Luxembourg)	10 ka ± 9 ka (2 SD)	M	22-fold	Lazaridis et al. (16)
*Stuttgart LBK*	N/A	Modern Human	Viesenhäuser Hof	Central Europe (Germany)	7 ka ± 7 ka (2 SD)	F	19-fold	Lazaridis et al. (16)

“Abbreviation” indicates the specimen designations proposed and used throughout the manuscript. Ages and associated uncertainty ranges (±2 SDs) are reported in thousands of years before present (ka). They are estimated from the branch shortening of nuclear genomes (*SI Appendix*, *SI Appendix* 6).

Here, we present the high-coverage genome of a male Neandertal from Denisova Cave and show that he is more closely related to the older Neandertal from Denisova Cave than to other Neandertals sequenced to date. We explore the population history of this Neandertal as well as the extent of genetic differentiation among Neandertal populations.

## Results

### DNA Extraction, Sequencing, and DNA Preservation.

The *Denisova 17* specimen is a small morphologically undiagnostic bone fragment reported to have been recovered from Layer 12 of the East Chamber of Denisova Cave (Fig. 1 *A* and *B* and *SI Appendix*, Fig. S1 and *SI Appendix* 1). It was excavated in 2011 and was identified as hominin using palaeoproteomics (ZooMS) (DC4969). Its mitochondrial (mt) genome has been sequenced and found to be of the Neandertal type (11).

As previously described (11), we removed 14.1 mg of the bone by drilling, extracted DNA, and generated a DNA sequencing library (*SI Appendix*, Table S1 and *SI Appendix* 2). Shotgun sequencing of the library revealed that 73% of DNA fragments longer than 35 nucleotides mapped uniquely to the human reference genome. At the 5’- and 3’-ends of the DNA molecules, 30% and 27% of the cytosine residues appeared as thymine residues, respectively, indicating that ancient human DNA is present in the library (*SI Appendix*, Fig. S3 and *SI Appendix* 3). We estimate contamination by present-day human DNA to be around 1% using four different methods based on the patterns of cytosine deamination and the probability that DNA sequences derive from present-day humans (*SI Appendix*, *SI Appendix* 5) (17–19).

We estimate that each milligram of bone powder contains 1.6 × 10^9^ DNA molecules, of which 51% pass all quality filters and align with high confidence to the human genome, corresponding to ~15-fold genomic coverage per milligram of bone powder. This remarkable preservation of ancient DNA allowed us to generate an average autosomal coverage of ~34-fold from a single library without sequencing it to exhaustion. By contrast, the other four high-coverage Neandertal and Denisovan genomes published to date required 5 to 20 libraries sequenced to exhaustion to reach comparable coverage. After filtering out regions where sequencing reads cannot be uniquely aligned, we genotyped 1,850,816,801 bases of the genome of *Denisova 17*, with an average read depth of 37-fold across the autosomes (*SI Appendix*, Fig. S5, Table S5, and *SI Appendix* 3).

### Sexing and Lineage Assignment.

Coverage of the X and Y chromosomes is roughly half of that of the autosomes (~17 - and ~16-fold, respectively), showing that *Denisova 17* comes from a male individual (*SI Appendix,* Tables S4 and S5). To determine whether the nuclear genome of this individual is closest to modern humans, Denisovans, or Neandertals, we analyzed genomic positions where at least one of two Neandertal genomes [*Denisova 5* (1); *Vindija 33.19* (2)], the Denisovan genome [*Denisova 3* (13)] or a present-day African genome (Mbuti, HGDP00982) carries a derived allele, while the genomes of chimpanzees and other primates (bonobo, gorilla, orangutan, rhesus macaque) carry the ancestral allele (20) (*SI Appendix*, *SI Appendix* 4). The *Denisova 17* genome carries 91% of the derived alleles found in both Neandertals, as well as 45% and 20% of those unique to *Denisova 5* and *Vindija 33.19*, respectively (*SI Appendix,* Fig. S9). This indicates that *Denisova 17* was a Neandertal more closely related to *Denisova 5* than to *Vindija 33.19*.

### Specimen Terminology.

To improve clarity when referring to archaic genomes, especially given that both Neandertals and Denisovans have been recovered from Denisova Cave, we adopt a simplified terminology. The newly sequenced Neandertal genome is referred to as “Neandertal *D17*” and the previously sequenced Neandertal genomes as “Neandertal *D5*” (formerly the “Altai Neandertal” or “*Denisova 5*”), “Neandertal *Vi33.19*” (Vindija 33.19) and “Neandertal *Chag8*” (*Chagyrskaya 8*), while the Denisovan genome is here referred to as “Denisovan *D3*.”

### MtDNA and Y Chromosome.

Previous analyses of the mt genome of Neandertal *D17* have shown that it falls outside the variation of most of the Neandertals from western Eurasia (Fig. 1*H*). The only exceptions are two 120,000-y-old Neandertals: the Hohlenstein-Stadel specimen from Germany whose mtDNA is an outgroup to all other Neandertal mtDNAs and the Scladina individual from Belgium, whose mtDNA clusters with Neandertal D5 mtDNA (11).

While the Neandertal *D17* Y chromosome is more closely related to other Neandertal Y chromosomes than to those of modern humans or Denisovans, it falls outside the variation of previously sequenced Neandertal Y chromosomes (Fig. 1*I*) (4, 7, 12). We estimate that the Neandertal *D17* Y chromosome shared a common ancestor with the other Neandertal Y chromosome about 166 ± 21 thousand years ago (kya), whereas previously sequenced Neandertal Y chromosomes share a common ancestor with each other around 121 ± 16 kya (Fig. 1*I*). We estimate the age of the common ancestor of the Y chromosome of *D17* and modern humans to around 395 ± 44 kya, consistent with previous estimates of the divergence between Neandertal and modern human Y chromosomes (Fig. 1*I*) (7, 12).

### Genetic Dating.

By counting the number of transversion substitutions on autosomes that occurred since the last common ancestor with chimpanzees, we estimate the age of the Neandertal *D17* genome to 110 ky (99 to 120 ky, 2 SD) (*SI Appendix*, *SI Appendix* 6) (2). This date is consistent with both the estimates obtained for the mt genome 132 ky (95% High Posterior Density Interval: 92 to 175 ky) (11) and with the age estimate of ~120 ky (95% High Posterior Density Interval: 100 to 142 ky) for the Y chromosome (Fig. 1*I*).

We note that the confidence intervals of the autosomal age estimates for *D17* and *D5* largely overlap, but the point estimate of *D17* is 6 to 9 ky younger than that of *D5* (Fig. 1*F* and *SI Appendix*, Table S9 and *SI Appendix* 6). This is consistent with the molecular age difference obtained from the mtDNA, which indicates that *D17* is 4 to 7 ky younger than *D5* (11).

### Population Relationships.

To estimate how *D17* is related to other Neandertals whose genomes have been sequenced to high-coverage, we compare the number of derived substitutions that these genomes share [*D-*statistics (21) and *SI Appendix*, *SI Appendix* 7]. We find that *D17* shares significantly more derived substitutions with the Neandertal *D5* than with *Vi33.19* or with *Chag8*, while *D5* similarly shares more derived alleles with *D17* than with other Neandertals (Fig. 1 *C* and *D*). This pattern is consistent with the lineage assignment described above and with analyses of sites heterozygous in *D17 and D5* (*SI Appendix*, *SI Appendix* 10–12).

Consistent with the difference in their ages, we find no long segments identical by descent that would indicate close kinship between the Neandertals *D5* and *D17* (*SI Appendix*, *SI Appendix* 8). Furthermore, demographic modeling approaches (19, 22) show that *D5* is not a direct ancestor of *D17* and that the populations represented by these individuals diverged approximately 7 ky after their common ancestor split from the population ancestral to *Chag8* and *Vi33.19* (Fig. 1*G*) (*SI Appendix*, *SI Appendix* 10, 12, and 13).

### Heterozygosity and Population Structure.

**Fig. 2.**
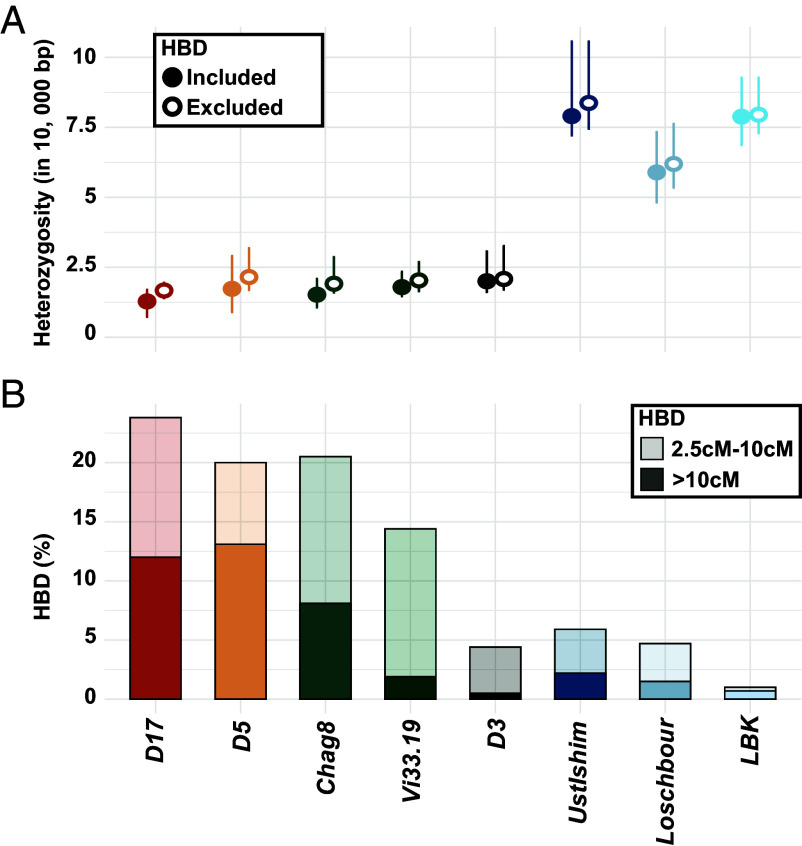
Genome wide heterozygosity and HBD tracts in Neandertal *D17* and other ancient human genomes (Neandertals D5, *Chag8*, *Vi33.19*; Denisovan *D3*; modern humans *Ust-Ishim*, *Loschbour*, and *LBK*). (*A*) Genome-wide heterozygosity for each individual computed with (filled circles) or without (open circles) inclusion of HBD tracks. (*B*) Proportion of HBD tracts in each genome. Proportion of each genome spanned by HBD tracts longer than 2.5 cM. Lighter shading indicates HBD tracts between 2.5 cM and 10 cM; darker shading indicates tracts >10 cM.

In the *D17* genome, ~1.2 of every 10,000 nucleotide positions are heterozygous, compared to 1.7 in the *D5* genome. By contrast, in early modern human genomes, 6 to 8 positions per 10,000 nucleotides are heterozygous (Fig. 2*A* and *SI Appendix*, *SI Appendix* 9). The fraction of the genome located in homozygous chromosomal segments resulting from mating between related individuals is 24% in the Neandertal *D17,* 20% in *D5* and *Chag8,* and 14% in *Vi33.19.* In contrast, in the Denisovan *D3* it is 4% and in early modern humans between 1% and 6% (Fig. 2*B* and *SI Appendix*, *SI Appendix* 9). This indicates that Neandertals lived in smaller groups than modern humans and that this may have been particularly typical of Neandertals in the Altai Mountains, where recent ancestors of *D5, D17,* and *Chag8* were close kin, such as first cousins or double first cousins (*SI Appendix*, *SI Appendix* 9) (1).

To investigate the structure and connectivity among archaic human subpopulations, we modeled the metapopulations from which archaic genomes are drawn, varying subpopulation sizes and number (and thus the total size of the metapopulation), as well as migration rates among them, to maximize the likelihood of the observed tracts of homozygosity in each genome, as previously described (3) (*SI Appendix*, *SI Appendix* 14). The results suggest that the Neandertals *D17*, *D5,* and *Chag8* differ from the Neandertal *Vi33.19* in that the former individuals lived in population sizes below 50 individuals under realistic scenarios of migration rate while the latter, as well as a 45,000-y-old modern human from Russia (15), lived in population sizes larger than 50 (*SI Appendix,* Figs. S36 and S40). This suggests that Neandertals in the east, represented by *D17*, *D5,* and *Chag8,* may have differed from those in the west, represented by *Vi33.19*, in that they lived in smaller and more isolated populations.

### Genetic Differentiation among Neandertals.

We quantify the genetic differentiation among Neandertals by estimating the fixation index (F_ST_), which measures the proportion of genetic variation explained by population structure (23). When doing this, the fact that the archaic populations are represented by single genomes presents two challenges. First, Wright’s original estimator of F_ST_ (24) is upwardly biased for small samples, a limitation that can be addressed by the Hudson estimator (25). Second, small sample sizes yield large SEs in allele-frequency estimates. However, this is mitigated by including large numbers of variants across the genomes.

**Fig. 3.**
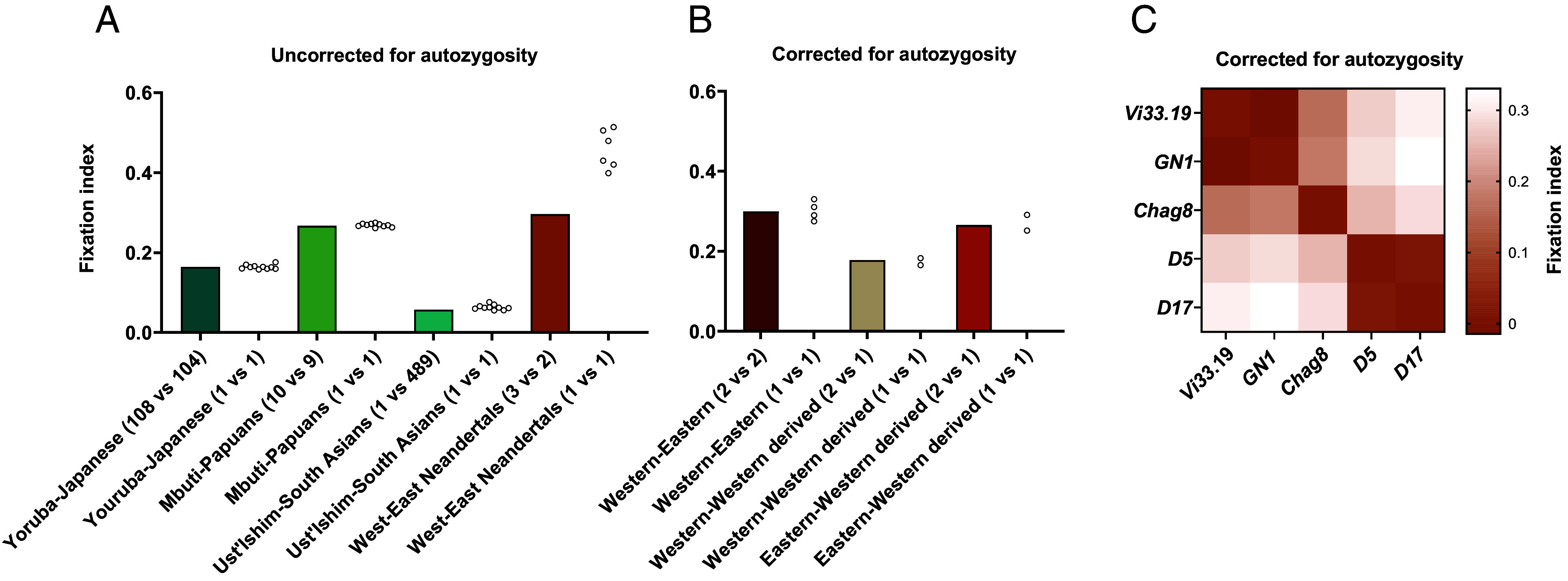
Fixation indices for modern and Neandertal population. (*A*) Fixation indices (Hudson’s F_ST_) for different population pairs. Bars show estimates based on multiple genomes per population, while open circles indicate values based on single-genome comparisons. Note the apparent inflation of F_ST_ between *Western* (*Chag8*, *GN1*, *Vi33*.*19*) and *Eastern* (*D5*, *D17*) Neandertals when using single genomes. (*B*) F_ST_ between the three Neandertal groups corrected for autozygosity. Bars represent estimates based on several genomes, while open circles show subsampled single-genome comparisons. (*C*) Heatmap of pairwise F_ST_ among high-coverage Neandertal genomes, corrected for autozygosity. Three clusters are evident: *Western* (*Vi33.19, GN1*), *Western*-derived (*Chag8*), and *Eastern* (*D5*, *D17*).

We assessed the reliability of the Hudson estimator when single genomes per population are used by comparing F_ST_ estimates between 108 Yoruba and 104 Japanese genomes, the population pair with the highest F_ST_ in the 1000 Genomes Project (26, 27), to F_ST_ values from random subsamples of single genomes from each population. As shown in Fig. 3*A*, single genomes reproduce the estimate from the full cohorts (F_ST_ = 0.16, 95% CI: 0.16 to 0.17). Similarly, F_ST_ estimates from single genomes matched those from the full sets of genomes when 10 Mbuti and 9 Papuan Highlanders were used (F_ST_ = 0.27, 95% CI: 0.26 to 0.27), another pair of populations which are highly differentiated (28). These analyses show that the Hudson estimator behaves well when single genomes per population are used.

However, using single Neandertal genomes results in F_ST_ estimates that are inflated compared to when multiple genomes are used (Fig. 3*A*). This may be explained by the high level of autozygosity in Neandertals, i.e., that chromosomal segment in an individual share recent ancestry resulting in that they are not independent observations of allele frequencies. To account for this, we estimated the degree of autozygosity and adjusted the effective number of observations accordingly (*SI Appendix*, *SI Appendix* 15). After correction, F_ST_ values calculated between single genomes faithfully reproduce those calculated with all available Neandertal genomes (Fig. 3*B*).

When pairwise F_ST_ values are calculated among currently available high-quality Neandertal genomes, three clusters of genomes showing little differentiation within them are observed (Fig. 3*C*). First, F_ST_ is close to zero (95% CI: 0.00 to 0.014) between the Neandertals *Vi33.19* and *GN1*, an ~45,000-y-old Neandertal genome from Belgium sequenced to ~22-fold coverage that recently became available (ENA accession PRJEB98484). Henceforth, we refer to these as *Western* Neandertals. Similarly, F_ST_ is close to zero between *D5* and *D17* (95% CI: 0.00 to 0.066). We refer to these as *Eastern* Neandertals. Third, *Chag8* forms a distinct cluster, which we term *Western-derived* Neandertals, as *Chag8* shares ancestry with *Western* Neandertals yet is differentiated from those (95% CI: 0.15 to 0.20). *Western*-*derived* Neandertals are thought to have replaced the *Eastern* Neandertals in the Altai Mountains, and presumably elsewhere, sometime between ~110,000 and ~70,000 y ago.

### Denisovan Ancestry.

**Fig. 4.**
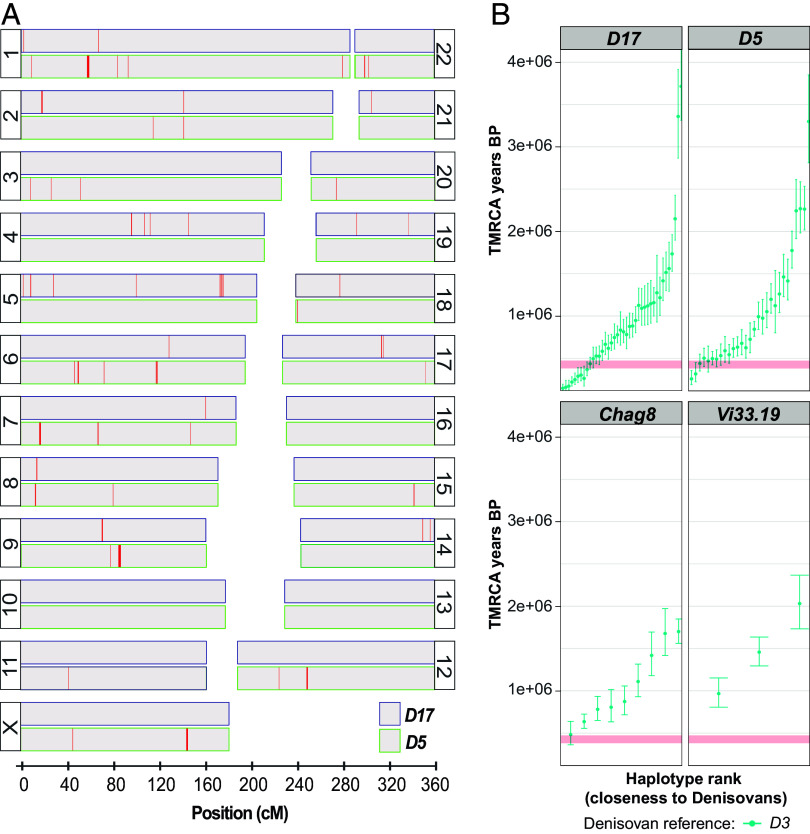
Denisovan ancestry in the Neandertals *D17* and *D5*. (*A*) Genomic distribution of introgressed Denisovan DNA segments longer than 0.2 cM (red vertical lines) across autosomal and X chromosomes in Neandertal *D17* (*Top*, blue frame) and *D5* (*Bottom*, green frame). (*B*) Estimated times to the most recent common ancestors (TMRCA) between each of the putatively introgressed homozygous Denisovan segments in Neandertals *D17*, *D5*, *Chag8,* and *Vi33*.*19* and the corresponding segments in Denisovan *D3*. The estimated split time between Neandertals and Denisovans is marked by a horizontal red band.

Using a hidden Markov model that infers local ancestry along genomes (29), we find that the *D17* and *D5* genomes contain chromosomal segments longer than 0.2 cM that indicate gene flow from Denisovans (Fig. 4). The locations of the Denisovan-like segments in *D17* and *D5* are significantly correlated with each other, suggesting that at least some of the gene flow traces back to the same admixture events (*SI Appendix*, *SI Appendix* 16).

We estimate the time from the Denisovan introgression to the time when the *D5* and *D17* individuals lived using the distribution of genetic lengths of the Denisovan-like segments in the two genomes. Although the exact dates depend on the length cut-offs used to distinguish introgressed segments from segments inherited from the common ancestors of Neandertals and Denisovans, as well as the genetic maps used, the time since introgression estimated for *D5* is always shorter than for *D17*. Using a conservative length cut-off, a generation time of 29 y, and assuming shared introgression events, the results indicate that *D5* lived ~8,000 y earlier than *D17* (*SI Appendix*, *SI Appendix* 16).

In the *Chag8* and *Vi33.19* genomes, we find a few segments with similarity to the Denisovan genome that are shorter than those seen in the *D5* and *D17* genomes. To determine if they are inherited from the common ancestors of Neandertals and Denisovans or if they come from Denisovan introgression, we estimate the time to their most recent common ancestor (TMRCA) with the matching sequences in the Denisovan *D3* genome (*SI Appendix*, *SI Appendix* 16). To avoid ambiguity about chromosomal phasing, we restrict these analyses to homozygous segments in the Neandertal genomes. Introgressed Denisovan segments can have a TMRCA younger than the Denisovan-Neandertal divergence, whereas the TMRCA of segments inherited from the common ancestor are, by necessity, of similar age or older than the Denisovan-Neandertal divergence. While several segments in the Neandertal *D5* and *D17* genomes have a TMRCA younger than the Neandertal–Denisovan divergence, indicating introgression from Denisovans, this is not the case for any of the segments in the *Chag8* or *Vi33.19* genomes (Fig. 4*B*). Thus, although it has previously been suggested that there is evidence for Denisovan admixture in the *Chag8* genome (7), we find clear evidence of recent Denisovan gene flow only in the Neandertals *D5* and *D17*.

### Interactions with Modern Humans.

In addition to Denisovan gene flow, we detect gene flow into the ancestors of *D17* and *D5* from a group related to early modern humans (*SI Appendix*, *SI Appendix* 17). This is consistent with previous work (30–33) and with demographic modeling (*SI Appendix*, *SI Appendix* 10 and 13), which identify modern human-like ancestry in Neandertals from introgression events ~200 kya.

In addition, we investigate how different Neandertal genomes are related to the Neandertal populations that contributed DNA to present-day humans. We used introgressed Neandertal genomic tracts identified in 4,091 non-African genomes from the 1000 Genomes Project (27) and the HGDP datasets (34) and counted the number of single-nucleotide variants in these tracts that matched each Neandertal genome, assigning each tract to the Neandertal genome with the highest number of matching variants (*SI Appendix*, *SI Appendix* 17). The largest number of tracts match *Western* Neandertals, particularly the *Vi33.19* genome, consistent with *Vi33.19* being the closest to the Neandertals that contributed ancestry to present-day non-Africans (2) (*SI Appendix*, Fig. S48 and *SI Appendix* 17). In contrast, the number of Neandertal tracts in present-day human genomes matching the *Eastern* Neandertals is about four times smaller.

## Discussion

As the second Neandertal older than 100,000 y for which a high-quality genome has been determined, the Neandertal *D17* provides a number of insights into Neandertal population history.

First, it is striking that the allele frequency differentiation between *Eastern* Neandertals (*D5* and *D17)* and *Western* Neandertals (*Vi33.19* and others) (F_ST_ = 0.30, 95% CI: 0.29 to 0.31) exceeds that of even the most differentiated pairs of present-day populations (28, 35, 36), such as the Mbuti of Central Africa and the Papuan Highlanders of New Guinea (F_ST_ = 0.27, 95% CI: 0.26 to 0.27). The divergence between Mbuti and Papuan is estimated to have occurred 130 to 220 kya (37–39), resulting in separate genetic drift along the two lineages over 260 to 440 ky. The divergence between *Eastern* and *Western* Neandertals occurred about 35 ky before *D5* and *D17* and about 80 ky before *Vi33.19* lived (2) resulting in separate genetic drift along the two Neandertal lineages over about 115 ky. This suggests that Neandertal populations reached greater levels of differentiation over shorter timescales than modern humans did. This is also illustrated by the modest differentiation between a ~45,000-y-old modern human genome from Siberia (*Ust’Ishim*) (15) and present-day populations (Fig. 3*A*) (F_ST_ = 0.052, 95% CI: 0.049 to 0.056). The relatively large genetic differentiation among Neandertal populations is consistent with smaller effective population sizes and with models of group sizes in Neandertals based on the distribution of homozygous tracts in their genomes (*SI Appendix*, Fig. S36 and *SI Appendix* 14), which would have increased genetic drift and resulted in greater allele frequency differences over time. It is also compatible with the observation that at least one late Neandertal population in Western Europe may have been isolated over long time (9) as well as with other work (40). Thus, in contrast to modern humans, Neandertals may often have been divided into distinct regional groups, despite being separated by relatively modest geographic distances.

Second, it is interesting that the Neandertals in the Altai Mountains lived in groups that were smaller than later Neandertals in the west (*SI Appendix*, *SI Appendix* 14). This is not only true for the *Eastern* Neandertals represented by *D5* and *D17*, who lived between 120,000 and 110,000 y ago, but also for the *Western-derived* population in the Altai Mountains represented by *Chag8* (3), who lived about 80,000 y ago as well as for Denisovans from the same cave (14). As both Neandertal and Denisovan populations during the earlier periods were small, it is possible that the environmental conditions at those times did not sustain larger groups in the Altai region. However, genomes from more well-dated individuals are needed to elucidate if population sizes correlate with changing environmental conditions or other factors.

Third, the replacement of the older *Eastern* Neandertal population in the Altai region by a *Western-derived* Neandertal population, represented by *Chag8,* occurred without any detectable mixing between the two groups (Fig. 1 *C* and *D*). This may indicate that the two Neandertal populations did not meet, perhaps because the older Neandertal population disappeared before the younger population from the west appeared in the Altai Mountains.

Fourth, we find clear evidence for gene flow from Denisovans into the older *Eastern* Neandertals *D5* and *D17*, whereas the evidence in the younger *Chag8* is tenuous. This is surprising as skeletal remains and sedimentary DNA from Denisova Cave show that Denisovans were in the Altai region from 250 kya to 60 kya (11, 13, 41), i.e., both before and after *Chag8* lived. It may also be surprising given that a first-generation offspring of a Denisovan father and Neandertal mother, whose age is comparable to that of *Chag8* (3, 42), has been found in Denisova Cave. The lack of Denisovan ancestry in *Chag8* genome could indicate that their ancestors were recent arrivals in the region. However, any speculations are limited by the fact that very few genomes spanning time scales of thousands of years are currently available. In the future, an alternative approach such as dense sampling of sediments from many archaeological sites in the region (43, 44) may clarify the distribution and overlap of Neandertals and Denisovans in the Altai.

In conclusion, our results suggest that Neandertal populations accumulated allele frequency differences more rapidly than the ancestors of present-day human groups. This indicates that the population history of modern humans was different from that of Neandertals and perhaps other archaic groups in that modern human populations were never small enough to allow drift on the scale that affected the Neandertals, even when modern humans left Africa and subsequently colonized new landmasses.

## Materials

The high-coverage Neandertal genome presented in this study was generated from DNA extracted from a morphologically uninformative bone fragment identified as hominin by ZooMS among bone fragments from layer 12 in the East Chamber of Denisova Cave (11). Details are provided in *SI Appendix*, *SI Appendix* 1.

## Methods

### DNA Extraction, Sequencing, Genotyping.

Bone powder was collected from the specimen using a dental drill. DNA was extracted following the “Dabney” protocol for the purification of short DNA fragments (45). Single-stranded sequencing libraries were prepared using an established protocol (46). DNA extraction purification and library preparation were performed using an automated liquid-handling platform (46, 47). Shotgun sequencing was conducted on a NovaSeq Illumina platform using a paired-end configuration of 2 × 76 bp + 8 cycles for the insert and index reads. Details are provided in *SI Appendix*, *SI Appendix* 2.

Base calling was performed using Bustard (Illumina). Overlapping paired-end reads were merged using LeeHom with the “ancientdna” parameters (48). Reads were mapped to the revised human reference genome (hg19) using Burrows-Wheeler Aligner BWA (49) with parameters “-n 0.01 –o 2 –l 16500” (13). Basic statistics to estimate endogenous aDNA content, duplicate removal, fragment length distribution, cytosine-to-thymine substitution frequencies, and genetic sex determination were obtained using in-house perl scripts as described previously (13, 46). The scripts used are implemented in C++ as “the Ancient DNA C++ tools” suite (50). Genotypes were called at each position of the genome using snpAD (version 0.3.11) (51). Reads were further filtered for genomic mappability (map35_99%), tandem repeat, indels and GC-corrected coverage thresholds following previously described procedures (1). Details are provided in *SI Appendix*, *SI Appendix* 3.

Subsequent analyses were conducted using previously published high-coverage ancient nuclear genome of Neandertals (1–3), Denisovans (13, 14), and modern human (15, 16) as well as genomic data from present-day modern human (13, 27, 34, 36, 52). Details are provided in *SI Appendix*.

### Lineage Assignment.

The *D17* individual was identified as a Neandertal by estimating the proportion of shared alleles with high-quality genomes of Neandertal (*D5* and *Vi33.19*), Denisovan (*D3*), and a modern human (African Mbuti). Analyses were restricted to diagnostic positions, defined as sites where one or more reference individuals carry a derived allele while chimpanzees and other primates carry the ancestral allele (20). Details are provided in *SI Appendix*, *SI Appendix* 4.

### Contamination Estimates.

Estimates of present-day human DNA contamination were obtained using heterozygosity observed in the mt DNA and Y chromosome. Additional contamination estimates were based conditional cytosine-to-thymine misincorporation patterns (20), AuthentiCT (17), and Cecast (19), a maximum likelihood framework that compares the observed alleles to expectations under a coalescent model using a panel of present-day human reference populations. Details are provided in *SI Appendix*, *SI Appendix* 5 and 18.

### Genetic Dating.

Molecular ages were estimated by counting the number of derived alleles accumulated in each ancient genomes since their divergence with great apes, as previously described (2). Age estimates were calibrated by comparison to the number of derived alleles observed in present-day African individuals. Details are provided in *SI Appendix*, *SI Appendix* 6.

### Population Relationship.

Relationship among ancient human genomes mt DNA and Y chromosome were inferred using comparative datasets through phylogenetic analyses under a Bayesian framework implemented in BEAST2 (53). We estimated the time to the most recent common ancestor among Neandertal Y chromosome sequences as previously described (12). Details are provided in ref. 11 and *SI Appendix*, *SI Appendix* 18.

Population relationships based on nuclear genomic data were assessed by estimating allele sharing between ancient human genomes using *D-statistics* implemented in Admixtools (21). Relatedness between close individuals was inferred by measuring how much Identity-by-descent segments they share using *KIN*, a method to infer kinship from aDNA genomes (54). Demographic modeling and split time between high-coverage archaic genomes were estimated using *Cecast* (19) and *momi2* (22). Relative genetic affinities between individuals were quantified using the F(A|B) statistics, as previously described (1). Details are provided in *SI Appendix*, *SI Appendix* 7, 8, and 10–13.

### Population Demography and Structure.

Relative group size among individuals was inferred from genome-wide measures of heterozygosity and from the length and distribution of homozygosity-by-descent (HBD) segments, as previously described (1). HBD patterns were also analyzed within a metapopulation framework using coalescent simulations to model the expected distribution of HBD tract lengths under varying group sizes and migration rates, with likelihoods estimated by comparing observed and simulated HBD tract proportions. Population sizes were also estimated using *momi2* based on sites frequency spectra across individuals under specified models of population history (22). Changes in effective population size through time were inferred using PSMC. Details are provided in *SI Appendix*, *SI Appendix* 9 and 14.

### Genetic Differentiation among Neandertals.

F_ST_ was estimated between pairs of single individuals, using genome-wide SNPs using the Hudson estimator (23, 25). To account for elevated levels of autozygosity in Neandertal genomes, F_ST_ estimates were corrected for the proportion of the genome affected by HBD. Details are provided in *SI Appendix*, *SI Appendix* 15.

### Denisovan and Modern Human Ancestry in Neandertal Genomes.

Denisovan and modern human ancestry in Neandertal genomes was analyzed using a hidden Markov model to infer local ancestry along the genome (29). To distinguish introgression from incomplete lineage sorting, the time to the most recent common ancestor (TMRCA) between Denisovan-introgressed segments and the corresponding sequences in Denisovan genomes was estimated and compared to the Neandertal–Denisovan split time. We assessed whether Denisovan ancestry observed in different Neandertal genomes derived from shared introgression events by measuring whether Denisovan-introgressed segments longer than 0.2 cM overlap between genomes more frequently than expected by chance, using bootstrap reshuffling of segments across the genomes as previously described (55). The timing of Denisovan introgression was inferred from the distribution of genetic lengths of introgressed segments. Details are provided in *SI Appendix*, *SI Appendix* 16 and 17.

## Supplementary Material

Appendix 01 (PDF)

## Data Availability

DNA sequence data have been deposited in ENA (PRJEB108287) (56).
