# Supplementary material for: A high-coverage Neandertal genome from the Altai Mountains reveals population structure among Neandertals
Source: Proc Natl Acad Sci U S A. 2026 Mar 23;123(13):e2534576123. doi: 10.1073/pnas.2534576123 (PMC13037865; doi:10.1073/pnas.2534576123)
Supplement: Supplementary file 1 — Appendix 01 (PDF) [file pnas.2534576123.sapp.pdf]

A high-coverage Neandertal genome from the Altai Mountains reveals population structure among Neandertals.

Diyendo Massilani[1], Stéphane Peyrégne[2], Leonardo N.M. Iasi[2], Cesare de Filippo[2], Fabrizio Mafessoni[3], Alba Bossoms Mesa[2], Arev Pelin Sümer[2], Yaniv Swiel[2], Divyaratan Popli[2], Shahar Silverman[1], Michael James Boyle[1], Maxim B. Kozlikin[4], Michael V. Shunkov[4], Anatoly P. Derevianko[4], Tom Higham[5,6], Katerina Douka[5,6], Matthias Meyer[2], Hugo Zeberg[2], Janet Kelso[2], Svante Pääbo[2]

1. Department of Genetics, Yale School of Medicine, New Haven, CT, USA
2. Department of Evolutionary Genetics, Max Planck Institute for Evolutionary Anthropology, Leipzig, Germany
3. Department of Life Sciences, University of Trieste, Italy
4. Institute of Archaeology and Ethnography of the Siberian Branch of the Russian Academy of Sciences, Novosibirsk, Russia
5. Department of Evolutionary Anthropology, Faculty of Life Sciences, University of Vienna, Vienna, Austria
6. Human Evolution and Archaeological Sciences (HEAS) network, University of Vienna, Vienna, Austria

Corresponding authors: Diyendo Massilani, Svante Pääbo

Email: diyendo.massilani@yale.edu, paabo@eva.mpg.de

This PDF file includes:

- Supporting text
- Figures S1 to S55
- Tables S1 to S44
- SI References

Other supporting materials for this manuscript include the following:

- Datasets: tba

Table of Contents

SI Appendix 1 – Material 3

SI Appendix2 – Sampling, DNA Extraction and Sequencing 5

SI Appendix 3 – Data Processing, Genotyping, General Filters 7

SI Appendix 4 – Lineage Assignment 15

SI Appendix 5 – Contamination 17

SI Appendix 6 – Genetic Dating of D17 21

SI Appendix 7 – Allele sharing between D17 and the other high-coverage archaic human genomes 27

SI Appendix 8 – Relatedness between Neandertals D17 and D5 30

SI Appendix 9 – Heterozygosity and Inbreeding 35

SI Appendix 10 – Estimates of split time between D17 and other archaic genomes using cecast 40

SI Appendix 11 – Archaic human effective population sizes inferred by PSMC 43

SI Appendix 12 – Estimation of split time using F(A|B) statistics 51

SI Appendix 13 – Demographic history and divergence between the Neandertal D17 and other high coverage archaic genomes inferred from momi2 56

SI Appendix 14 – Population structure and effective population size estimates 64

SI Appendix 15 – Genetic differentiation among Neandertals 70

SI Appendix 16 – Denisovan ancestry in Neandertals D17 and D5 73

SI Appendix 17 – Neandertals and modern human admixture 86

SI Appendix 18 – The Y chromosome of the Neandertal D17 91

References 107

Supporting Information Text

SI Appendix 1 – Material

Diyendo Massilani

Archaeological context, peptide mass fingerprinting and mitochondrial genome analyzes.

D17 (*DC4969*) is a non-diagnostic bone fragment of approximately 30 mm in length and 6-8 mm in width (Fig. S1), which was discovered within the bulk fragment collection from layer 12 of the East Chamber of Denisova Cave (Altai Mountains, southern Siberia) (1). The specimen was identified to be of hominin origin through ZooMS analysis (ZooMS ID: DC4969), and mitochondrial genome reconstruction revealed its maternal lineage to be Neandertal (1). Using the newly generated shotgun DNA sequences, we reconstructed the complete mitochondrial sequence of the individual to 1,384-fold coverage and found it to be identical to the previously published sequence determined via hybridization capture (1). Peptide mass fingerprinting and mitochondrial analyses and phylogeny of *D17* are detailed by in Brown et al. in 2021 (1).

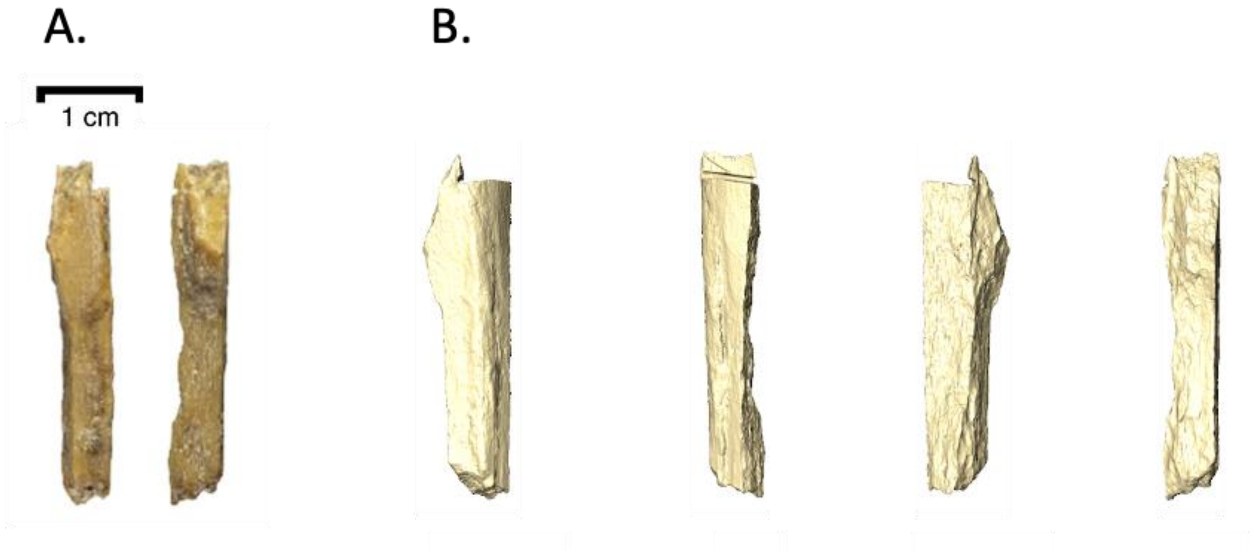

Figure S1. *D17* bone fragment. A) Photography of the bone fragment. B) 3D surface images obtained from microCT scanning(1).

SI Appendix 2 – Sampling, DNA Extraction and Sequencing

Diyendo Massilani

Prior to sampling for ancient DNA analyses, the *D17* bone fragment was µCT-scanned at the Max Planck Institute for the Science of Human History in Jena, Germany (1).

Sampling and DNA extraction

All sample processing prior to DNA amplification were performed in the ancient DNA clean room facility at the Max Planck Institute for Evolutionary Anthropology in Leipzig, Germany.
A sample of 14.1mg of bone powder was removed at one extremity of the bone fragment using a sterile dental drill. DNA extraction was carried out using silica-coated magnetic particles and a protocol optimized for retrieving short DNA molecules (2) on an automated liquid handling platform (3). Given the small amount of input bone powder, we adjusted the volume of the lysis buffer to 300 µL, of which 150 µL were used for DNA extraction into a final volume of 30 µL.

DNA Library preparation and sequencing

The 30 µL of purified DNA extract was converted into a Next Generation Sequencing (NGS) library using a single-stranded approach (4, 5). Using qPCR, we estimated that the library (‘A26088’) contained 11.3 billion DNA molecules (Table S1). Throughout all experimental steps, extraction and library negative controls were included. Additionally, a short oligonucleotide was spiked into the *D17* library and the negative control library to assess the efficiency of the library preparation. The incorporation rate of the short oligonucleotide in the *D17* extract was estimated to 99.1%, indicating no detectable inhibition in the sample. Libraries amplification was performed using a double indexing scheme (6), as detailed in Gansauge et al., 2020 (5). The library was then paired end sequenced on a NovaSeq Illumina platform.

Table S1. DNA extract and library prepared from the *D17* bone fragment.

	Extract ID	Bone powder (mg)	Input in library (mg)	Library ID	Number of DNA molecules in library	Number of control oligo in the library	Library preparation efficiency
Denisova 17	E16282	14.1	7.05	A26088	$1.13x10^{10}$	$1.21x10^{6}$	99.10%
Extraction negative control	E16297	NA	NA	A26103	$1.03x10^{8}$	$1.22x10^{6}$	NA

NA=Not Applicable

SI Appendix 3 – Data Processing, Genotyping, General Filters

Diyendo Massilani, Stéphane Peyrégne, Janet Kelso

Data Processing

We generated 4.69x10^9 sequencing reads from the library using a paired-end configuration of 2 x 76 bp + 8 cycles for the insert and index reads on the NovaSeq platform (Illumina). Base calling was performed using *Bustard* (Illumina). Only sequences with the expected index reads, allowing for a maximum of one mismatch, were retained. Adapter sequences were removed, and overlapping paired-end reads were merged using *LeeHom* with the “ancientdna” parameters (7). The DNA sequences were then mapped to the revised human reference genome sequence *hg19* with an added decoy sequence (8) using Burrows-Wheeler Aligner BWA (9) with parameters « -n 0.01 –o 2 –l 16500 » (10). Sequences with identical alignment start and end positions were collapsed into single sequences by consensus calling using bam-rmdup (https://github.com/mpieva/biohazard-tools). Sequences shorter than 35 bases and sequences with a mapping quality lower than 25 were excluded. Summary statistics, including the number of reads generated, endogenous DNA content and complexity, duplication rate, average read size, and read size distribution (Fig. S2), proportion of sequences with apparent cytosine (C) to thymine (T) substitutions at their ends (Fig. S3), were obtained using an in-house *perl* scripts as previously described (4, 11) (Table S2, S3). These scripts are now implemented in C++ and are distributed as part of the Ancient DNA C++ tools suite (12), a collection of high-performance utilities optimized for large-scale processing and basic statistical analysis of aDNA sequencing data. For each chromosome, average coverage and standard deviation were estimated using *samtools* depth version 1.3 (13) (Table S3). The average coverage of chromosome X is approximately half that of the autosomes, indicating that *D17* bone fragment belonged to a male individual (Table S4). To reduce alignment artifacts, reads near indels were realigned using GATK’s Indel Realigner version 1.3-14 (14).

Genotyping

Genotypes were called at each position with a base quality of at least 30 using the ancient DNA genotype caller snpAD (version 0.3.11) (15).

General Filters

Following the methodology in earlier high-coverage archaic genomes, we applied the following filters to minimize mapping errors.

- **Genome mappability “map35_99%”**: Retains genomic regions where at least 99% of all possible 35-base-long sequences (35-mers) can be uniquely mapped to the reference genome (16).
- **Tandem Repeat**: Removing all regions identified as repetitive according to the Tandem Repeats Finder (17)

- **Coverage:** We exclude sites with a coverage of less than 10x for autosomes and less than 5x for the sex chromosomes.
- **Indels:** We exclude from the analyses all indel differences to the human reference genome, unless otherwise specified.
- **Coverage and GC content:** Previous high-coverage archaic genomes exhibit a coverage bias, with higher coverage in regions of low GC-content and lower coverage in region of high G-C content. To mitigate this effect, sites falling outside the 2.5% and 97.5% quartiles of the coverage distribution were excluded from analyses of these genomes. *D17* differs from the other high coverage archaic genome in that the data doesn't show a higher coverage for region with low GC-content (Fig. S4). When considering GC-content of sliding window of 51 bases along the genome, *D17* coverage is similar for window of GC-content of 10% to 60% (representing 90,68% of the data) and start to decrease for window of GC-content over 60% (Fig. S4). Similarly to other high coverage archaic human genome, we excluded sites from the *D17* sites that do not fall within the 2.5% and 97.5% quartiles of the coverage distribution. However, to mitigate the less pronounced bias in *D17* compared to other archaic genomes, for some analyses, we excluded sites falling outside the 0.5% and 97.5% quartiles of the coverage distribution. The specific interval of the coverage filter used is specified for each analysis.

After filtering for genome mappability and tandem repeats, we retained a total of 1,850,816,801 bases, with an average coverage, genotype quality and GC content of 37x, 142 and 41%, respectively (Table S5, Fig. S5, and Fig. S6). For heterozygous calls, we observed on average 2.01 more transitions than transversions (Table S5, Fig. S7). At these positions, the reference allelic balance is 0.5214 indicating a reference to alternative coverage ratio of 1.089, meaning that reference alleles have on average 8.9% more coverage that the alternative, suggesting slight reference mapping bias (Fig. S8).

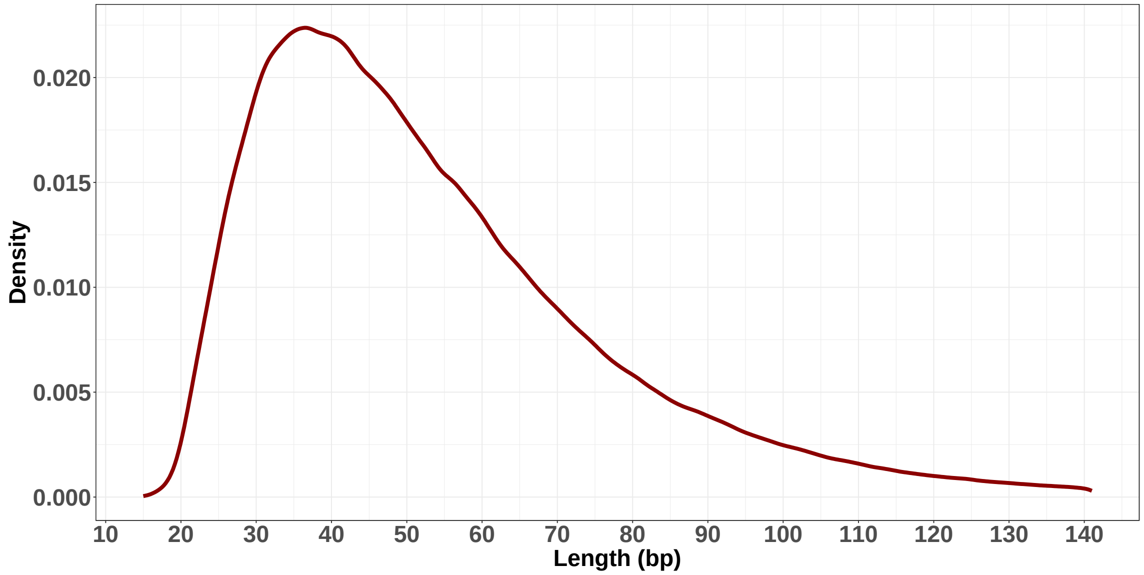

Figure S2. Size distribution of reads from *D17*. The distribution was obtained using a subsample of 5 million mapped sequences with mapping quality of at least 25 (MAPQ 25).

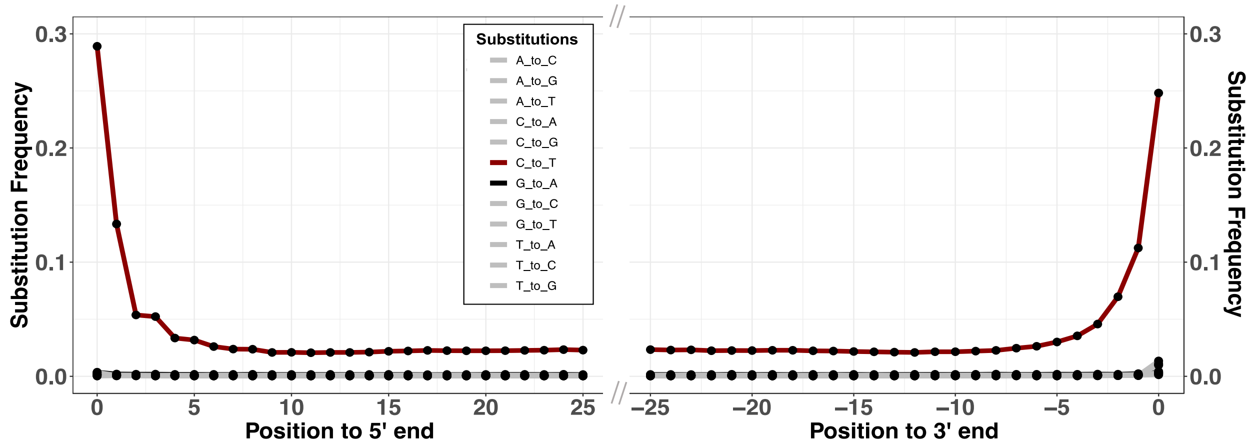

Figure S3. Substitution frequency at the first and last 25 bases of the reads from *D17* when compared to the human reference sequence *hg19*. The estimates were inferred from a subsample of 5 million reads after filtering for MAPQ 25 and minimal size of 35 bases.

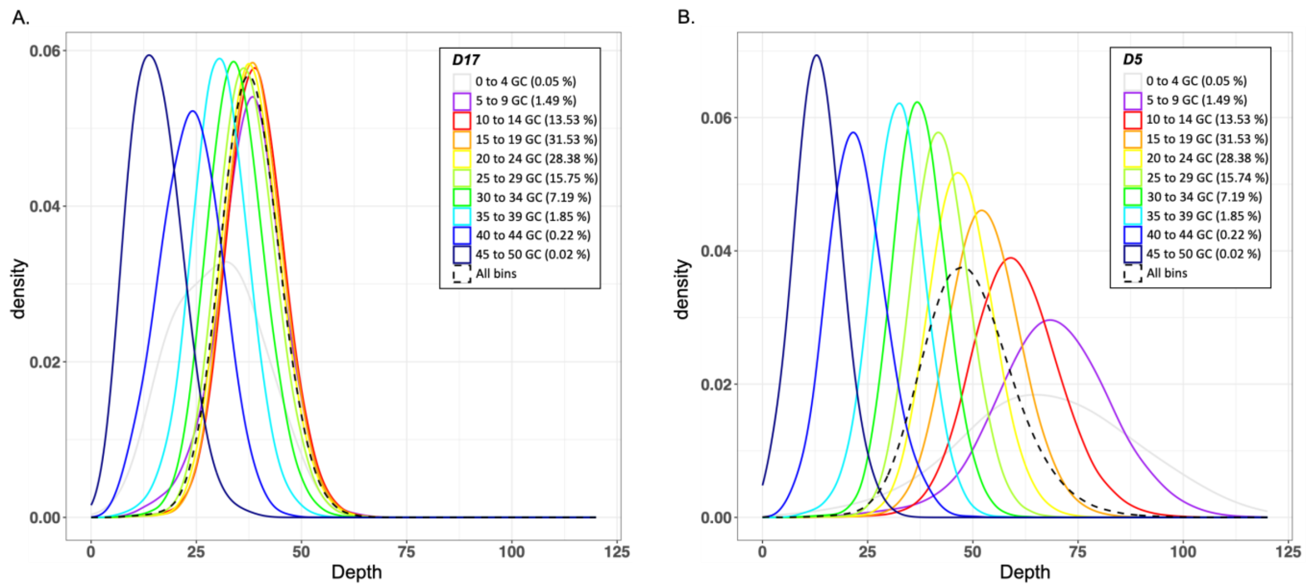

Figure S4. Coverage density for various GC content bins assessed by estimating the number of G or C bases in sliding windows of length 51bp along chromosome 21 for **A)** *D17* (*Denisova 17*) and **B)** *D5* (*Denisova 5*). The curves represent the coverage distribution for 10 different GC content bins (0 to 4 G or C bases per 51bp window, 5 to 9 G or C bases per 51bp window, 10 to 14, and so on). The proportion of sequences in each GC content bin is reported in parentheses. The coverage tends to be higher for bins with lower GC content. This effect is more pronounced for *D5* and previously published archaic genomes than for *D17*, for which coverage is consistent for bins with GC ranging from 5 to 29, which corresponds to 90,68 % of all sequences. A GC bias is noticeable only for bins with GC content greater than 30.

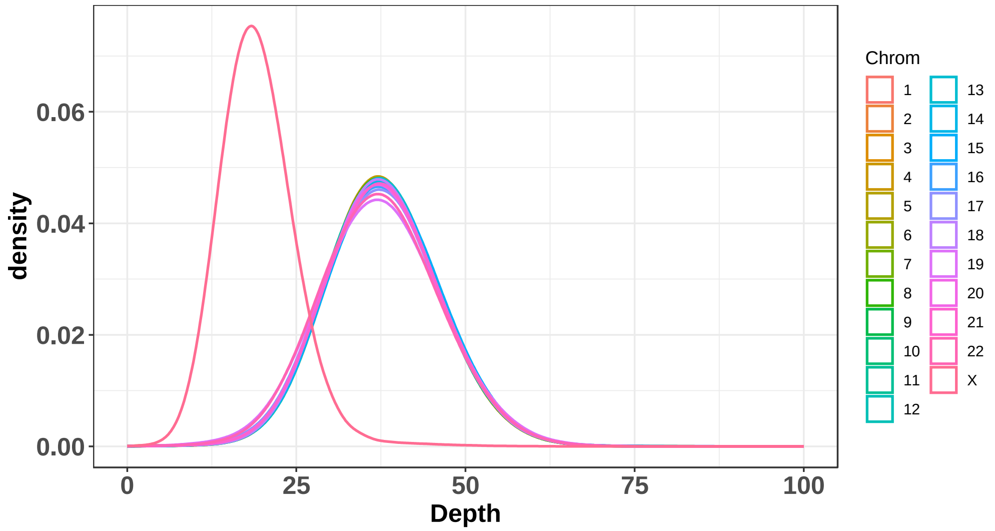

Figure S5. Depth coverage of bases called from each chromosome of the *D17* genome. The graph was made by using 1% of the data.

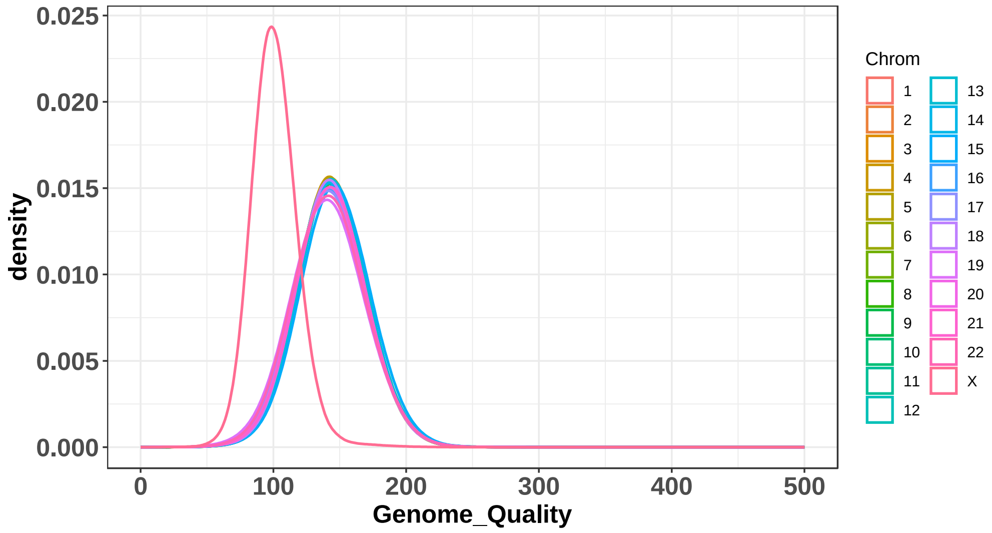

Figure S6. Genotype quality of bases called from each chromosome of the *D17* genome. The graph was made by using 1% of the data.

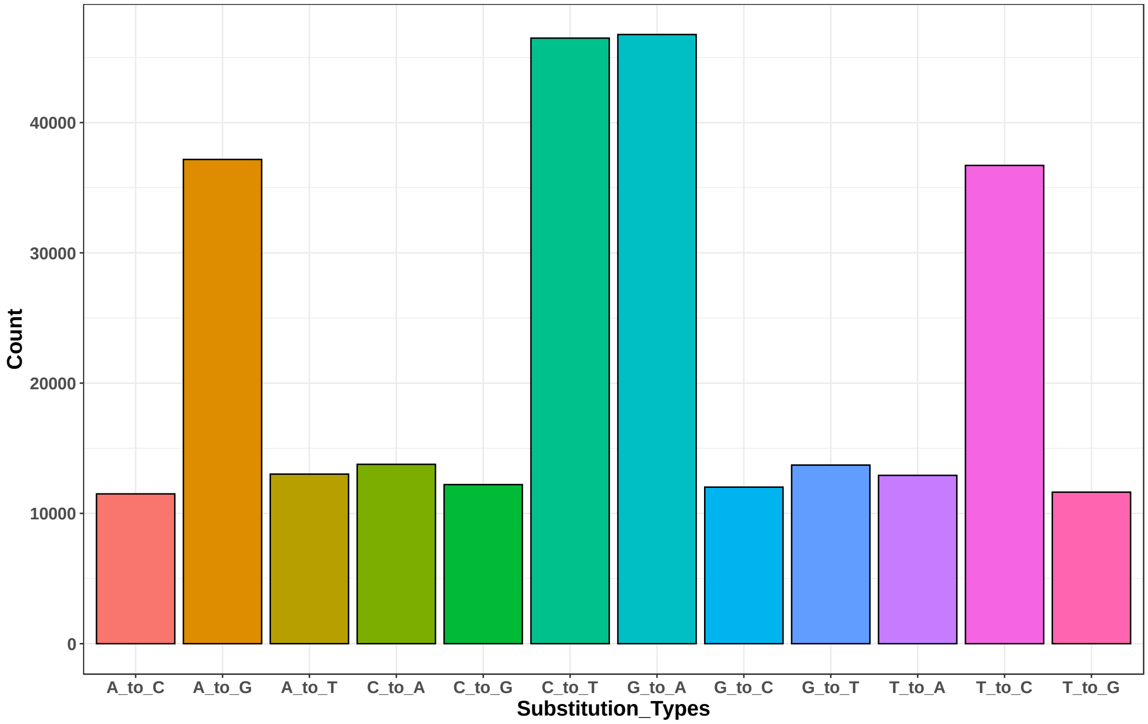

Figure S7. Count of each type of nucleotide difference at heterozygous positions in the *D17* genome.

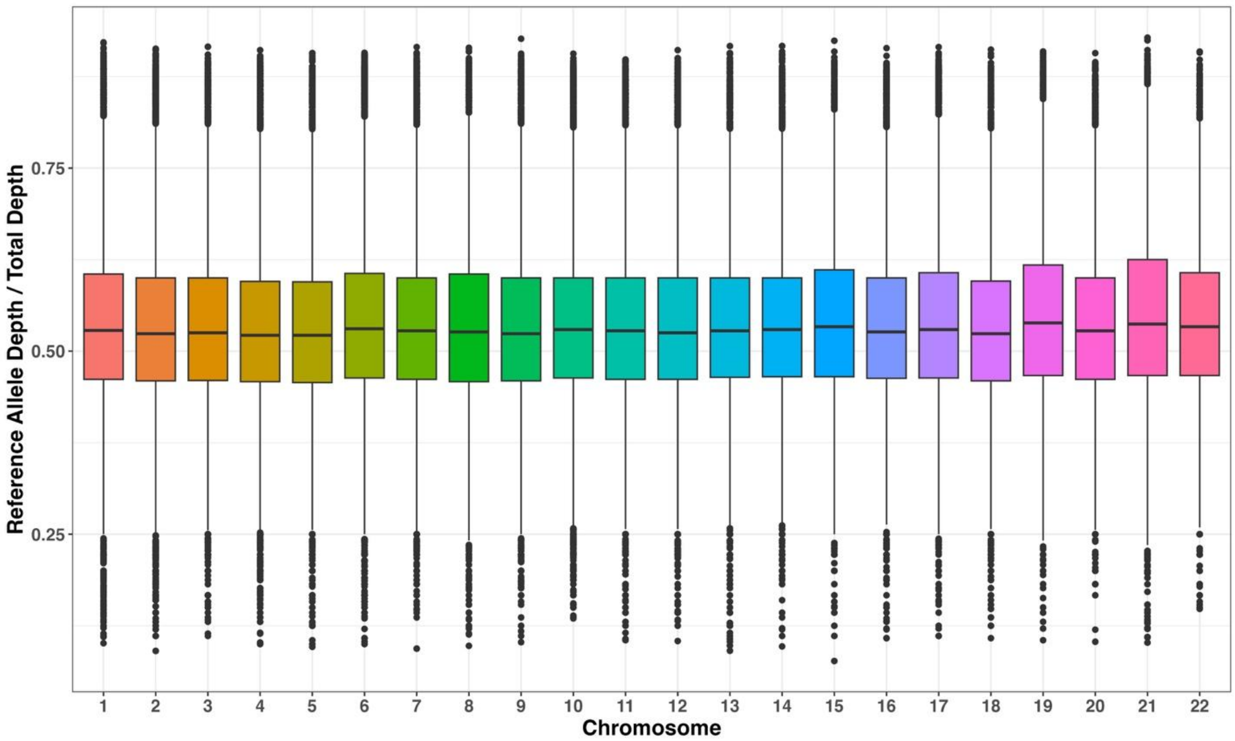

Figure S8. Allelic balance at heterozygous positions for each chromosome of the *D17* genome. The allelic balance is estimated by the ratio of the number of reads supporting the reference allele to the total number of reads covering the position

Table S2. DNA library characteristics

Sample	Sequencing Platform	Total nbr of seq. generated	Total nbr of seq. merged and filtered	Total mapped seq. (MAPQ 25 and L35)	% mapped seq.	Unique mapped seq. (MAPQ 25 and L35)	Dup. rate	Av. Size (MAPQ25 and L35)
D17 lib A26088	NovaSeq	4,833,913,824	3,370,394,065	2,391,808,803	70.96	1,601,003,236	1.49	59.54

Sequences merged and filtered for mapping quality 25 (MAPQ 25) and minimal size of 35 bases (L35). Seq=Sequences, nbr = number, deam = sequences showing evidence for deamination (C to T mismatch) at the first or last base, Dup.= duplication, NA = Not Applicable.

Table S3. Cytosine (C) to Thymine (T) substitution frequency

Sample	% 5' C to T	% 3' C to T	95% CI 5' C to T	95% CI 3' C to T
D17 lib A26088	28.9	24.8	28.8 – 29.0	24.7 – 24.9

The %5' and %3' C to T represent the frequency of cytosine (C) to thymine (T) mismatches to the reference at the first and last base of the sequences. The estimates were inferred from a subsample of 5 million reads after filtering for MAPQ 25 and minimal size of 35 bases. 95 CI = 95% Binomial Confidence Interval of the C to T mismatch frequency.

Table S4. Average coverage per chromosome

Chromosomes	Average coverage (x)	Standard deviation
1	34.72	12.68
2	35.06	11.89
3	34.96	11.71
4	34.90	11.84
5	34.93	11.69
6	35.01	11.69
7	34.42	12.29
8	34.96	11.70
9	34.74	11.94
10	34.73	12.12
11	34.51	12.02
12	34.46	12.27
13	35.19	11.57
14	34.66	12.10
15	34.76	12.50
16	33.98	12.81
17	33.56	13.37
18	35.18	11.46
19	31.42	14.18
20	34.65	12.04
21	34.73	12.30
22	33.40	12.92
X	17.43	7.08
Y	16.15	11.53

Table S5. *D17* genotype summary table

Chrom	Number of bases called	Depth	Genotype Quality	GC content	Homozygous alternative positions	Heterozygous positions	Number of Transversion (Tv)	number of Transition (Ti)	Ti/Tv ratio
**1**	1E+08	38.05	144.57	0.41	188159	22308	68704	141763	2.06
**2**	2E+08	38.00	144.77	0.40	210736	23163	78342	155557	1.99
**3**	1E+08	37.99	145.09	0.39	177428	17244	65804	128868	1.96
**4**	1E+08	37.93	144.02	0.37	180177	20889	68627	132439	1.93
**5**	1E+08	37.94	144.04	0.39	156805	19043	59514	116334	1.95
**6**	1E+08	38.15	145.20	0.39	156200	16510	57163	115547	2.02
**7**	1E+08	38.02	144.48	0.40	138530	14928	51355	102103	1.99
**8**	1E+08	37.91	145.13	0.40	146607	11985	55569	103023	1.85
**9**	7E+07	37.90	143.67	0.41	104530	12098	39741	76887	1.93
**10**	9E+07	38.04	143.95	0.41	122192	14581	44786	91987	2.05
**11**	9E+07	37.79	144.00	0.42	120798	12440	44359	88879	2.00
**12**	9E+07	37.99	144.34	0.40	108163	13185	39985	81363	2.03
**13**	7E+07	38.08	145.41	0.38	92811	8831	33793	67849	2.01
**14**	6E+07	37.97	144.34	0.40	77536	8923	28762	57697	2.01
**15**	5E+07	38.23	147.14	0.42	72395	4936	25929	51402	1.98
**16**	5E+07	37.83	142.99	0.45	76573	8961	30410	55124	1.81
**17**	5E+07	37.91	142.72	0.45	60932	9231	21956	48207	2.20
**18**	5E+07	37.90	143.65	0.39	72561	9136	26866	54831	2.04
**19**	3E+07	37.54	141.30	0.49	43125	5763	15623	33265	2.13
**20**	4E+07	37.82	143.43	0.44	57836	6572	20523	43885	2.14
**21**	2E+07	37.91	143.79	0.41	37688	3701	13669	27720	2.03
**22**	2E+07	37.45	142.12	0.49	31398	3327	10657	24068	2.26
**X**	9E+07	19.31	101.14	0.39	94238	1261	32679	62820	1.92
**All**	2E+09	37.12	142.23	0.41	2527418	269016	934816	1.86 E+06	2.01

Chrom=Chromosome

SI Appendix 4 – Lineage Assignment

Diyendo Massilani

To determine whether the *D17* individual is globally more related to modern humans, Denisovans, or Neandertals, we estimated the proportion of allele he shares with the high-quality genomes of the *D5* (“Altai/*Denisova 5*”) Neandertal (18), the Denisovan *D3* (10), the Vindija Neandertal Vi33.19 (8), and a present-day human from Africa (Mbuti, HGDP00982) (10) . We used positions defined as “diagnostic” in that one or more of these individuals carry a derived allele in either the homozygous or heterozygous state while the genomes of chimpanzees and other primates (bonobo, gorilla, orangutan, rhesus macaque) carry the ancestral allele (19).

At diagnostic positions, 1% of the *D17* sequences carry the derived alleles found in the present-day human Mbuti, 2% carry the derived alleles found in the Denisovan *D3* individual, 21% carry the derived alleles found in the Vindija Neandertal Vi33.19, and 46% carry the derived alleles found in the *D5* Neandertal (Fig. S9). The higher proportion of shared derived alleles with Neandertals, and particularly with the Neandertal *D5*, suggests that ***D17* is a Neandertal individual**, more closely related to the *D5* Neandertal than the *Vi33.19* Neandertal.

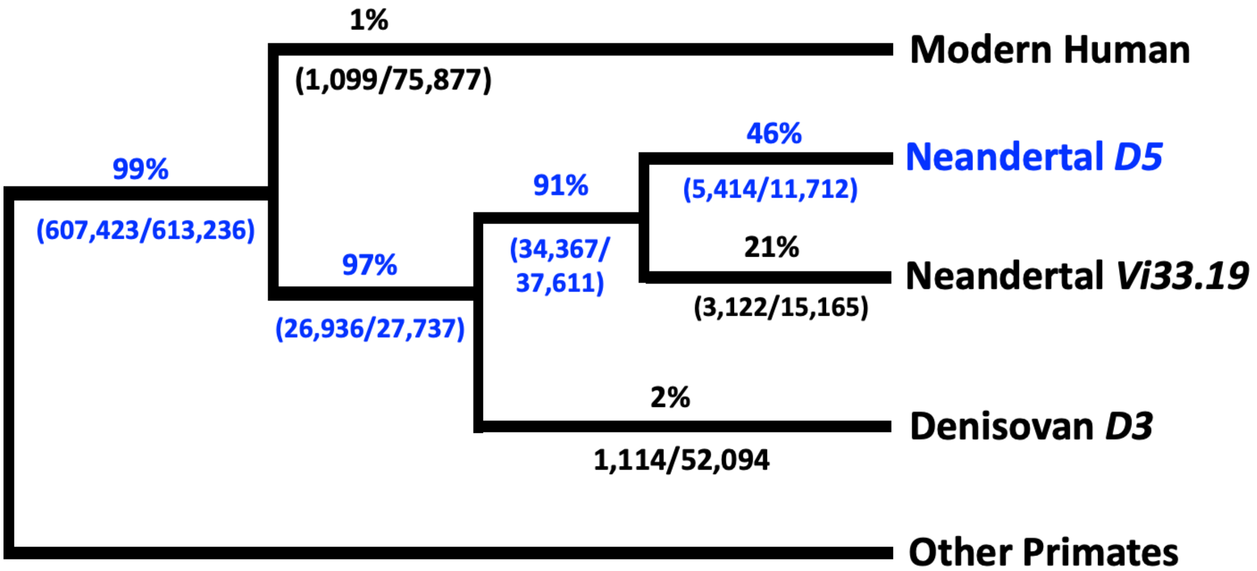

Figure S9. Hominin lineage assignment of the *D17* individual: The percentage of *D17* sequences that match the derived state at diagnostic positions for each branch of the tree is displayed above the respective branches, with the corresponding number of sequences shown below each branch. The assignment was made using a random subsample of 5 million sequences after filtering for MAPQ 25 and minimal size of 35 bases.

SI Appendix 5 – Contamination

Diyendo Massilani

We estimated present-day human DNA contamination in the *D17* genome using four complementary approaches: one based on heterozygosity observed in mitochondrial DNA, two based on characteristic aDNA damage patterns, and a fourth using a maximum likelihood framework that compares the observed alleles to the expectations predicted by a coalescent model, using a panel of diverse modern reference populations. The four approaches yield estimates of present-day human DNA contamination ranging from 0% to 1,9%.

Mitochondrial DNA contamination estimate

To estimate the mitochondrial DNA contamination, we realigned the newly generated shotgun DNA sequences to the mitochondrial revised Cambridge Reference Sequence (rCRS, NC_012920.1) using the Burrows-Wheeler Aligner (BWA) (9) with ancient DNA parameters « -n 0.01 –o 2 –l 16500 » (10). After filtering out reads shorter than 35 bases, retaining only those with a mapping quality of at least 25, removing duplicates and masking the first and last three bases of each read to minimize the impact of cytosine deamination, we reconstructed a consensus mitochondrial genome sequence for the individual to an average coverage of 1,384x (s.d. 206). We focused on 45 positions where the mitochondrial genomes of 25 previously sequenced Neandertals differ from those found in at least 99.5% of 56,427 present-day modern human mitochondrial genomes from the gnomAD database (20). Only positions with coverage within 10% of the average depth of the newly reconstructed genome were retained to ensure reliable genotype calls. At each position, we counted the total number of reads covering the site, those carrying the reference state and those carrying the alternative Neandertal allele (Table S6). We found a total of 996 reads carrying the reference state at all 45 positions and 52,556 reads with the alternative Neandertal allele, resulting in an estimate of mitochondrial DNA contamination rate of **1.9%** (95% binomial CI: 1.78%–2.02%).

Conditional C to T mismatch frequencies

We can assess modern DNA contamination by exploiting the characteristic cytosine-to-thymine (C→T) substitutions that accumulates due to deamination of cytosine residues at the ends of ancient DNA fragments (21, 22). Present-day DNA molecules that contaminate the sample carry virtually no such damage, so their presence should dilute the overall C→T frequency observed. To recover the true deamination rate of endogenous molecules, we first filtered for reads that show a C→T change in the first or last three bases, reads that are highly likely to be genuine ancient DNA (19). Under the assumption that damage at the two fragment ends occurs independently, we then calculated the “conditional” C to T frequency, i.e., the C to T rate at one end of fragments that carry a C to T change at the opposite end. In an uncontaminated sample this conditional rate should match the overall C to T rate; a higher conditional

rate indicates that undamaged contaminant fragments lower the overall rate and therefore that the samples are contaminated.

For *D17*, the conditional C to T frequencies are 24.4 % (95 % CI 24.2–24.7) at the 5′-ends and 20.9 % (95 % CI 20.6–21.1) at the 3′-ends (Table S7), significantly lower than the overall rates of 28.9 % and 24.8 %, respectively, computed from all fragments (Table S3). Because the conditional estimates do not exceed the overall ones, the data are consistent with none or at most very low non-detectable modern DNA contamination. A rough contamination fraction can be obtained as contamination (c) = 1 − (overall C to T / conditional C to T); in the case of *D17* the value is negative, reinforcing the conclusion that detectable contamination is absent. We stress that this estimate is meant only as an order-of-magnitude check; it ignores factors such as sequencing error, GC bias, and non-independent damage patterns, and should not be treated as a precise contamination estimate.

Contamination estimates with AuthentiCT

We estimated contamination using AuthentiCT, a computational model designed for single-stranded aDNA libraries that leverages C to T substitutions along the entire DNA fragments explicitly accounting for dependence among multiple damage sites (23). Using a subsample of five million reads from the *D17* library A26088, AuthentiCT estimates a contamination rate of **1.39 ± 0.87 %**, consistent with the low levels inferred by the other methods.

Contamination estimates with Cecast

Finally, we used Cecast, a maximum-likelihood framework that estimates the proportion of modern-human contamination in archaic genomes (https://github.com/cesaredef/cecast/tree/main) (24). The program compares the observed distribution of ancestral and derived alleles in the sample with expectations generated by coalescent simulations, testing them against a panel of high-coverage genomes that includes two present-day Africans (Mbuti, Yoruba), the Neandertals *D5*, *Vi33.19* and *Chag8* and the Denisovan *D3*. For the contamination component, Cecast evaluates a wider set of ten present-day human genomes (Mbuti, Yoruba, Sardinian, Dai, French, Han, Mandenka, Papuan, San and Karitiana) to identify the most likely source of modern DNA contamination.

Using five million reads from the *D17* library A26088, we run Cecast (i) filtering out reads with indels, (ii) randomly sampling one fragment per informative site ascertained to distinguish the reference genomes, (iii) excluding fragments that carry neither the ancestral nor the derived allele, and (iv) retaining only strand-specific reads longer than 35 bases with a map quality of at least 25. This results in an estimate of contamination of **0.07%** across the ten potential modern-human source populations, with a bootstrap 95% confidence interval of **0.01%–1.16%** (Table S8), indicating that present-day human contamination is negligible. Because the inferred contamination is so low, the log-likelihoods for the ten reference populations are essentially indistinguishable, not allowing any contaminant source to be identified.

Table S6. Mitochondrial contamination estimates for *Denisova 17*

Position on the rCRS mtDNA REF	REF base	ALT base	Number of informative reads at the position	Number of informative reads - REF state	Number of informative reads - ALT state	Number of informative reads - no REF and no ALT	Contamination Estimate	Contamination Estimate 95% CI (%)
243	A	G	1196	23	1173	7	1.92%	1.28 - 2.87
2523	C	T	1312	12	1300	9	0.91%	0.52 - 1.59
2831	G	C	972	0	972	44	0.00%	0 - 0.39
3399	A	G	1154	18	1136	3	1.56%	0.99 - 2.45
3414	C	T	995	1	994	0	0.10%	0.02 - 0.57
3909	C	T	982	2	980	2	0.20%	0.06 - 0.74
4204	T	C	1026	79	947	11	7.70%	6.22 - 9.49
4532	G	A	1190	49	1141	6	4.12%	3.13 - 5.40
4856	T	C	1190	33	1157	10	2.77%	1.98 - 3.87
4940	C	T	1396	5	1391	0	0.36%	0.15 - 0.84
5387	C	T	1095	2	1093	2	0.18%	0.05 - 0.66
5673	T	C	1288	90	1198	14	6.99%	5.72 - 8.51
5840	C	T	1209	5	1204	2	0.41%	0.18 - 0.96
6266	A	C	1114	0	1114	15	0.00%	0 - 0.34
6410	C	T	1346	7	1339	2	0.52%	0.25 - 1.07
6452	C	T	1403	10	1393	4	0.71%	0.39 - 1.31
6641	T	C	1251	43	1208	14	3.44%	2.56 - 4.60
7106	A	C	1200	63	1137	24	5.25%	4.12 - 6.66
7868	C	T	1153	3	1150	2	0.26%	0.09 - 0.76
7891	C	T	1197	5	1192	5	0.42%	0.18 - 0.97
8021	A	G	1273	13	1260	8	1.02%	0.60 - 1.74
8065	G	A	1289	0	1289	2	0.00%	0 - 0.30
8365	A	G	1278	94	1184	19	7.36%	6.05 - 8.92
8461	C	T	1312	2	1310	2	0.15%	0.04 - 0.55
9329	G	C	981	2	979	26	0.20%	0.06 - 0.74
9345	C	T	956	43	913	6	4.50%	3.36 - 6.00
9869	C	T	1509	9	1500	2	0.60%	0.31 - 1.13
10101	T	C	1032	35	997	2	3.39%	2.45 - 4.68
10281	C	T	993	1	992	1	0.10%	0.02 - 0.57
10307	C	T	964	2	962	1	0.21%	0.06 - 0.75
10324	T	C	998	21	977	4	2.10%	1.38 - 3.20
10532	A	G	1010	61	949	8	6.04%	4.73 - 7.68
11458	A	T	1080	51	1029	6	4.72%	3.61 - 6.16
11623	C	T	1416	31	1385	3	2.19%	1.55 - 3.09
11770	T	C	1261	25	1236	11	1.98%	1.35 - 2.91
12474	C	T	1421	0	1421	2	0.00%	0 - 0.27
13095	T	C	982	61	921	14	6.21%	4.87 - 7.90
13707	G	A	1161	4	1157	1	0.34%	0.13 - 0.88
13801	A	G	1178	13	1165	12	1.10%	0.65 - 1.88
13879	T	A	1268	7	1261	3	0.55%	0.27 - 1.14
15226	A	G	1075	14	1061	4	1.30%	0.78 - 2.17
15295	C	T	1157	3	1154	2	0.26%	0.09 - 0.76
15649	A	G	990	15	975	2	1.52%	0.92 - 2.48
15667	C	T	1133	5	1128	0	0.44%	0.19 - 1.03
16037	A	G	1170	34	1136	3	2.91%	2.09 - 4.03
**All 45 positions**	**NA**	**NA**	**52556**	**996**	**51560**	**320**	**1.90%**	**1.78 - 2.02**

rCRS: Revised Cambridge Reference Sequence; REF: Reference; ALT: Alternative

Table S7. Conditional C to T substitution frequency of *Denisova 17*

Sample	Cond. % 5' C to T	Cond. % 3' C to T	95% CI cond. 5' C to T	95% CI cond. 3' C to T
D17 lib A26088	24.4	20.9	24.2 – 24.7	20.6 – 21.1

The conditional %5' and %3' C to T give the frequency of cytosine (C) to thymine (T) mismatches to the reference at the first and last base of the sequences with C to T mismatches at the opposite end. After filtering the data for MAPQ ≥ 25 and a minimum sequence length of 35 bp, we drew a subsample of five million reads. Of these, 361,831 reads carried a C to T substitution at the 5′-ends and 258,724 reads carried a C to T substitution at the 3′-ends; these two subsets were then used to estimate the conditional C to T frequencies at the 3′- and 5′-ends, respectively. 95 CI = 95% Binomial Confidence Interval of the C to T mismatch frequency.

Table S8. Contamination estimate using *Cecast*

Sample	Contamination estimate	95% CI contamination estimate	Log likelihood	Potential contamination source	Number of informative sequences
D17 lib A26088	0.06%	0.01% – 0.84%	-827.0578	Mbuti	108,000
D17 lib A26088	0.07%	0.01% – 1.03%	-827.0454	Yoruba	108,000
D17 lib A26088	0.08%	0.01% – 1.08%	-827.0419	Dai	108,000
D17 lib A26088	0.08%	0.01% – 1.07%	-827.0407	French	108,000
D17 lib A26088	0.08%	0.01% – 1.1%	-827.0409	Han	108,000
D17 lib A26088	0.07%	0.01% – 1.03%	-827.0574	Mandenka	108,000
D17 lib A26088	0.08%	0.01% – 1.16%	-827.031	Papuan	108,000
D17 lib A26088	0.08%	0.015 – 1.08%	-827.0419	San	108,000
D17 lib A26088	0.08%	0.01% – 1.08%	-827.0419	Sardinian	108,000
D17 lib A26088	0.08%	0.01% – 1.09%	-827.0369	Karitiana	108,000

SI Appendix 6 – Genetic Dating of *D17*

Stéphane Peyrégne

We genetically dated the *D17* individual by counting derived alleles on his lineage since the last common ancestor with great apes, as previously described (8). This age estimate was calibrated by comparing the count of derived alleles to those computed from a present-day Mbuti individual (HGDP00982, SS6004471, or B_Mbuti-4 (10, 18)). For comparison, we also included four other high-coverage archaic human genomes (8, 10, 18, 25) and three ancient modern human genomes (Ust'-Ishim (26), Loschbour and Stuttgart "LBK" (27)).

For each genome, we only considered sites in the autosomes aligning to the reference genomes of four great apes (chimpanzee panTro4, bonobo, gorilla gorGor3, and orangutan ponAbe2), and where all four great apes carry the same allele (different from a gap "-"or a missing base "N"), which we considered to be the ancestral allele. We calculated the relative age of each ancient individual with the formula:

$$Relative\ age = \frac{n_M - n_A}{n_M}$$

With n_M = number of derived alleles in the Mbuti and n_A = number of derived alleles in the ancient genomes.

At heterozygous sites, we randomly sampled one allele. Estimates were converted to years assuming a divergence time between humans and chimpanzees of 13 million years, corresponding to a mutation rate of 0.5×10^{-9} per base pair per year (18). This calibration is based on direct estimates of per-generation mutation rates from pedigree sequencing studies (28–30) and their synthesis (31), as well as on the number of missing mutations in the high-quality genome of a ~45,000-year-old modern human from Ust'-Ishim (26). This mutation rate has been used in previous high-coverage Neandertal genome analyses (8, 18, 25) and may be more consistent with some aspects of the fossil record (31, 32).

Variance in the dating estimates was computed with a block-jackknife approach with a window size of 5Mb (33). Potential biases due to coverage differences among genomes were minimized by excluding positions outside the intersection of the basic filter of *D17* (SI Appendix 3) and those of the other genomes (8, 25). We further excluded transitions, which are prone to recurrent and back mutations.

While the dates estimated for the Denisovan *D3* and modern humans were consistent with published estimates (8, 26), those of Neandertals were consistently older by about 5,000 years (Fig. S10). As the only difference from previous analyses is the inclusion of *D17* and the restriction to sites that also pass the filters of this genome, we investigated the feature of these filters that may conceal derived alleles in Neandertals. We found that ignoring the GC-corrected coverage cutoffs of *D17* (while still applying the minimum 10x coverage cutoff) restored the previously estimated dates for Neandertals (Fig. S11). In particular, applying only lower minimum coverage cutoffs on the GC-corrected coverage distributions of *D17* yields older dates (Fig. S12), suggesting that mutations in the *D17* genome, and by extension

mutations shared with other Neandertals, are concealed by this cutoff. Although intended to mask heterozygous deletions and potential genotyping errors due to low coverage, the standard cutoff of removing the lowest 2.5% of the GC-corrected coverage distributions of *D17* introduces a reference bias, masking positions with mutations more often than positions that match the reference. This likely occurs because sequences with differences to the reference genome are more difficult to align, resulting in lower sequence coverage at positions with a mutation. This bias is exacerbated at positions with a high local GC content of 50% or higher, consistent with more frequent deamination-induced C-to-T substitutions that represent additional mismatches to the reference genome (Fig. S13). Moreover, the extent of allelic imbalance at heterozygous positions is consistent with a larger reference bias in the *D17* genome compared to the other archaic genomes (Fig. S14) as we observe in average 8.9% more sequences supporting the reference allele than the alternative allele. However, other factors such as differences in present-day human DNA contamination may contribute to these differences in allelic imbalance among these genomes. The reason why reference bias would be larger in *D17* is unclear.

In conclusion, mutations in *D17*, which are more often shared with Neandertals than with Denisovans or modern humans, are missing with the standard filter and affect the age estimates of other Neandertals. Using a lower minimum coverage cutoff (removing the lowest 0.5% of the distribution instead of 2.5%) for *D17* leads to dates for other Neandertal genomes (for which the filters were unchanged) that are consistent with previous estimates (Table S9; (8)), suggesting minimal reference bias in the *D17* genome using this filter. As other analyses may also be affected by the observed reference bias, throughout this study, we compared the results obtained with the standard filters and this version of the filter that includes a more lenient minimum GC corrected coverage cutoff. **Using the latter cutoff, we estimated that the *D17* individual lived 110ka (100ka-120ka, 2 s.d.),** overlapping with previous estimates based on the mitochondrial genome (132ka; 95% High Posterior Density Interval: 92ka-175ka) (1) and the Y chromosome (120ka; 95% High Posterior Density Interval: 100-142ka; SI Appendix 18). While the point estimate for the date of *D17* is nine thousand years more recent than for the *D5* Neandertal, the confidence intervals largely overlap precluding precise determination of the age difference, if any, between the two individuals.

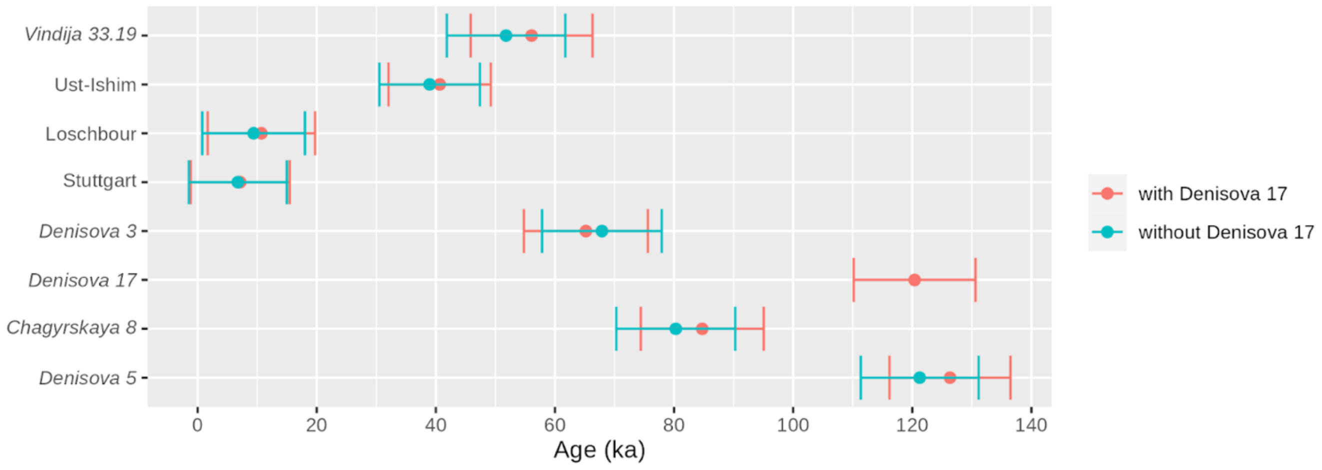

Figure S10: Autosomal branch shortening estimates with a bias for Neandertals. Bars correspond to two standard deviations computed with a block-Jackknife using a window size of 5Mb. We applied the minimal set of filters described in SI Appendix 3, but these filters conceal mutations in *D17* that are often shared with other Neandertals. As the bias is large enough to affect the estimates for other Neandertals, we disregard these estimates. Neandertals *D17* (Denisova17), *D5* Denisova5), Chag8 (Chagyskaya8), Vi33.19 (Vindija33.19); Denisovan D3 (Denisova3); Modern human UstIshim, Loschbour, Stuttgart LBK.

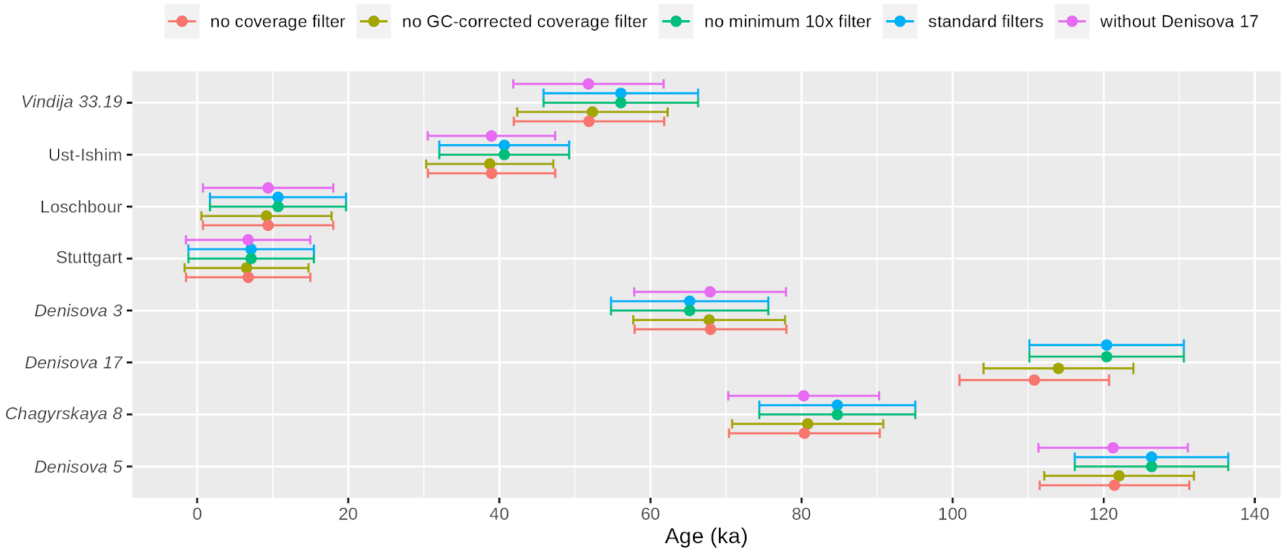

Figure S11: Autosomal branch shortening estimates without applying some coverage filters to *Denisova 17*. Bars correspond to two standard deviations computed with a block-Jackknife using a window size of 5Mb. Colours correspond to different filtering approaches where we did not apply the GC-corrected coverage filters and/or the minimum 10x coverage filter but applied the other minimal set of filters described in SI Appendix 3. For comparison, we include the estimates from Fig. S10 ("standard filters" and "without *D17*"). Neandertals *D17* (Denisova17), *D5* Denisova5), Chag8 (Chagyskaya8), Vi33.19 (Vindija33.19); Denisovan D3 (Denisova3); Modern human UstIshim, Loschbour, Stuttgart LBK.

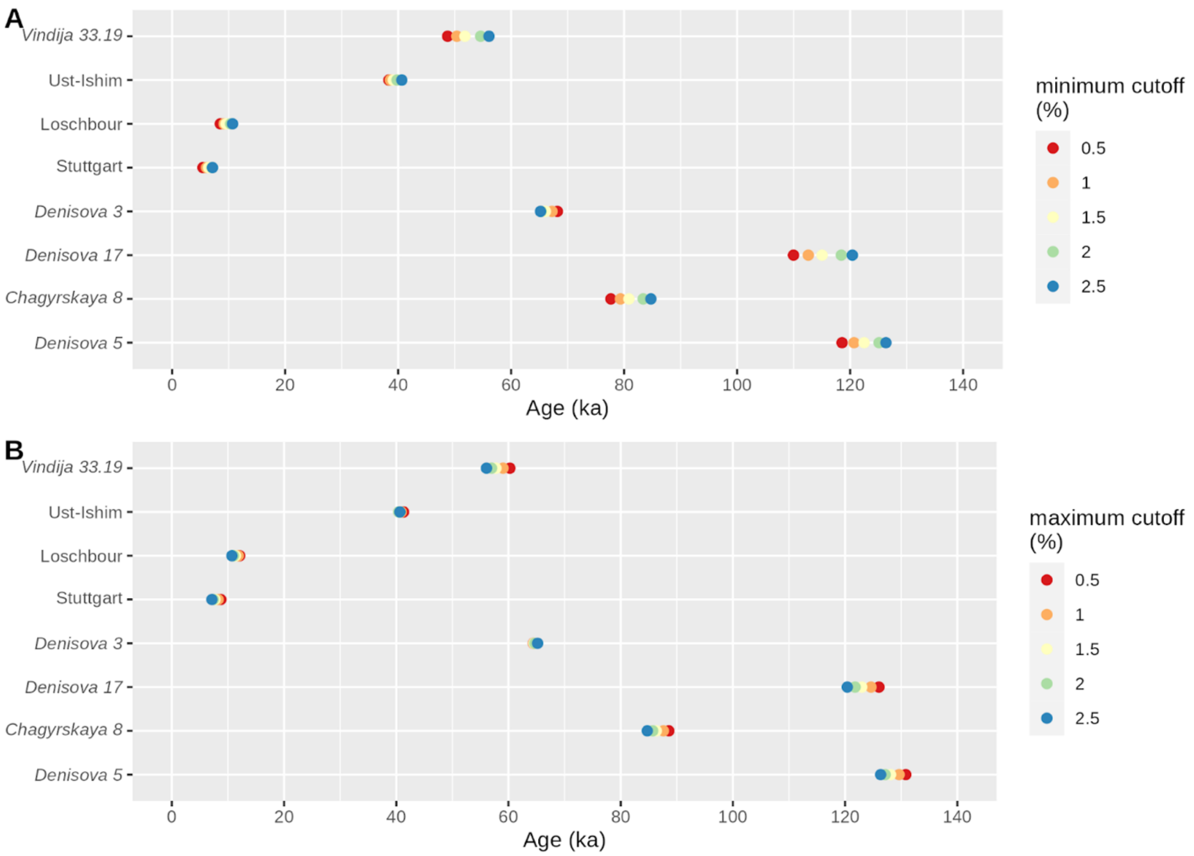

Figure S12: Effect of the GC-corrected coverage filters on the autosomal branch shortening estimates. We applied the minimal set of filters described in SI Appendix 3 (including the minimum 10x coverage cutoff) and minimum (A) or maximum (B) cutoffs (different colours) removing different proportions of the tails of the GC-corrected coverage distributions. Neandertals *D17* (Denisova17), *D5* Denisova5), Chag8 (Chagyskaya8), Vi33.19 (Vindija33.19); Denisovan D3 (Denisova3); Modern human UstIshim, Loschbour, Stuttgart LBK.

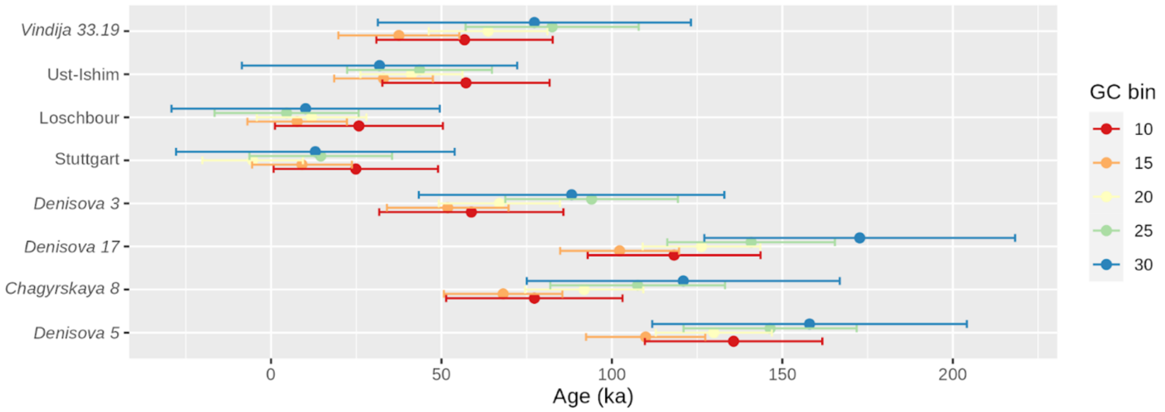

Figure S13: Effect of the local GC content on the autosomal branch shortening estimates. Each site was assigned to a GC bin based on the local GC content computed in a 51bp-window centered on that site. The numbers in the legend correspond to the count of G or C bases per 51bp window (e.g. 10 and 15 indicate 10-14 and 15-19 G or C bases, respectively). Branch shortening estimates were computed from the sites of each bin, but we excluded the bins with less than 10 or more than 34 G or C bases as they include too few sites to compute branch shortening. Bars correspond to two standard deviations computed with a block-Jackknife using a window size of 5Mb.

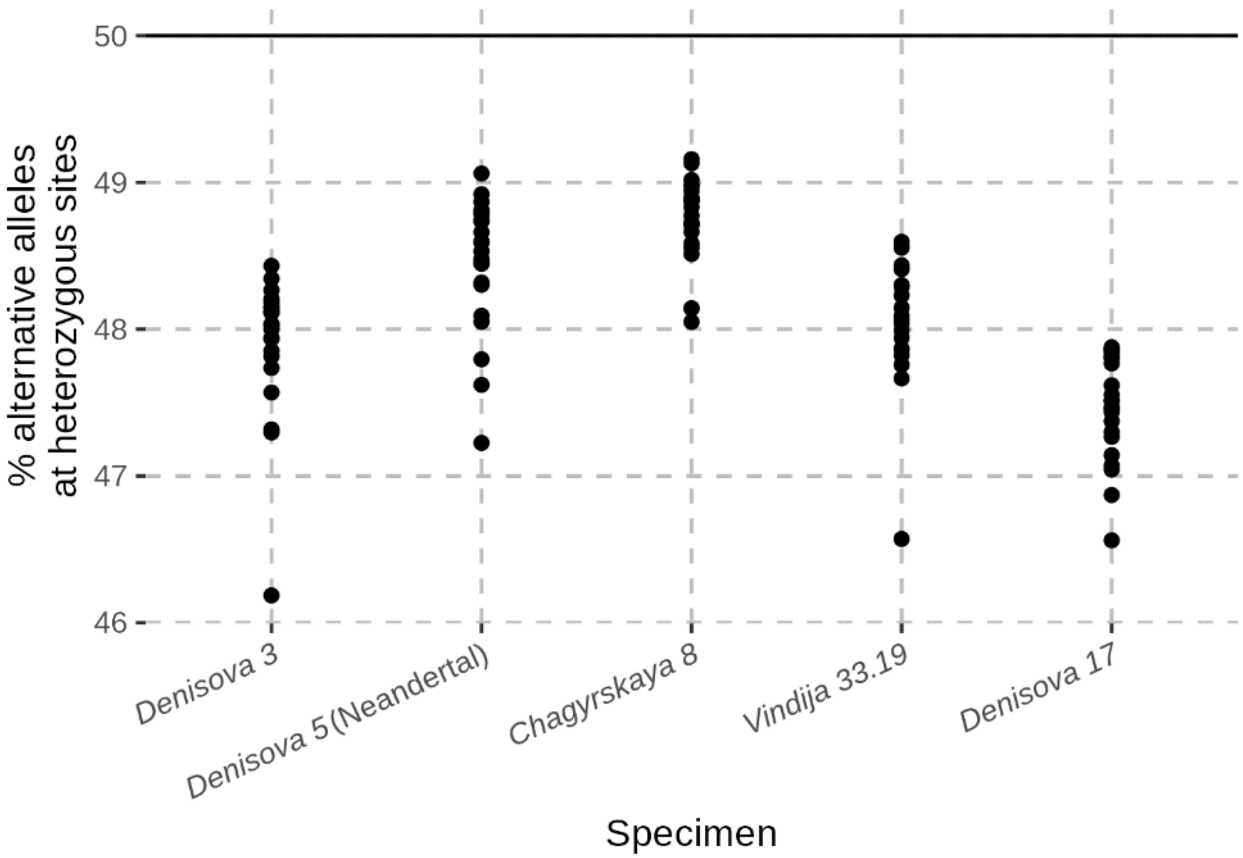

Figure S14: Allelic imbalance at heterozygous positions in archaic human genomes. Each dot represents the proportion of sequences that carry the alternative allele (with a base quality of at least 30) at the heterozygous positions of a given autosome. We only considered sequences that are at least 35bp, that align to the human genome with a mapping quality of at least 25, and that overlap positions that pass the minimal set of filters described in SI Appendix 3. Neandertals *D17* (Denisova 17), *D5* Denisova 5), Chag8 (Chagyskaya 8), Vi33.19 (Vindija 33.19); Denisovan D3 (Denisova 3).

Table S9: Autosomal branch shortening estimates. These estimates correspond to the data filtered with the standard set of filters or the set including the more lenient GC-corrected coverage cutoffs that remove for each GC bin the 0.5% positions with the lowest coverage and 2.5% positions with the highest coverage.

Genome	Filter	Branch shortening (2 s.d.)
Ust-Ishim	standard	40,648 (32,053-49,243)
	0.5% minimum cov. cutoff	38,360 (29,829-46,890)
Loschbour	standard	10,700 (1,694-19,706)
	0.5% minimum cov. cutoff	8,518 (0-17,340)
Stuttgart	standard	7,139 (0-15,447)
	0.5% minimum cov. cutoff	5,439 (0-13,716)
Neandertal *D5*	standard	126,338 (116,176-136,499)
	0.5% minimum cov. cutoff	118,566 (108,511-128,620)
Neandertal *Vi33.19*	standard	56,078 (45,851-66,305)
	0.5% minimum cov. cutoff	48,759 (38,633-58,884)
Neandertal *Chag8*	standard	84,737 (74,410-95,065)
	0.5% minimum cov. cutoff	77,652 (67,423-87,880)
Denisovan *D3*	standard	65,194 (54,784-75,603)
	0.5% minimum cov. cutoff	68,177 (57,912-78,443)
**Neandertal *D17***	**standard**	**120,391 (110,171-130,612)**
	**0.5% minimum cov. cutoff**	**109,977 (99,877-120,077)**

SI Appendix 7 – Allele sharing between D17 and the other high-coverage archaic human genomes

Divyaratan Popli, Diyendo Massilani

We investigated how the *D17* Neandertal genome is related to other high-coverage archaic human genomes (Neandertals *D5* (18), *Vi33.19* (8) and *Chag8* (25) and Denisovan *D3* (10)) by computing *D*-statistics of the form *D(Ind1, Ind2; Ind3; Outgroup).* The test measures whether Ind3 shares more derived allele with Ind1 or Ind2, relative to an outgroup, here the African Mbuti (34).

A significantly positive *D* indicates an excess of derived allele sharing between Ind3 and Ind1, whereas a significantly negative *D* indicates an excess of derived allele sharing between Ind3 and Ind2. A non-significative *D* (not significantly different from 0) indicates that Ind3 shares a comparable number of derived alleles with both Ind1 and Ind2. Standard errors (SE) were estimated via chromosome-based block-jackknifes, and Z-scores were computed as *D*/SE; |Z-score| >= 3 are considered significant (34).

Using autosomes only, we filtered each genome's VCF to remove indels and regions with abnormal coverage (SI Appendix 3). We removed monomorphic sites, converted the filtered VCF to EIGENSTRAT format, and computed *D*-statistics for different individual combinations using the qpDstat module of the ADMIXTOOLS suite (https://github.com/DReichLab/AdmixTools (35)) (Table S10, Fig. S15).

The results show that the *D17* genome share more alleles with the Neandertal *D5* than with any other high-coverage archaic genome (Fig. S15). *D*-statistic tests indicate that the two Denisova Cave Neandertals older than 100 ka, *D5* and *D17*, cluster together, whereas the ~80 ka Neandertal *Chag8* and ~50 ka Neandertal *Vi33.19* form a separate clade (Fig. S15), reflecting population structure between the early and late Neandertals sampled thus far.

The extent of shared derived alleles between each high-coverage Neandertal genome and the Denisovan *D3* is comparable, suggesting that none of these genomes shows a significant level of Denisovan introgression using this test.

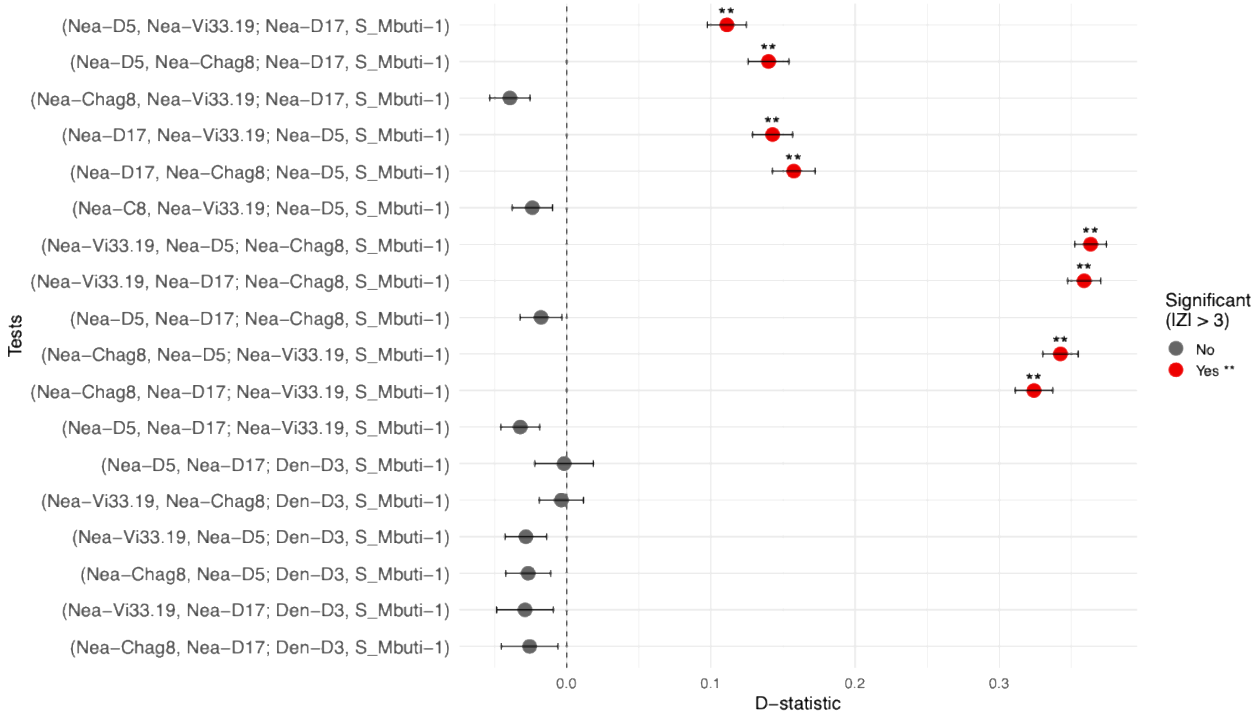

Figure S15. Allele sharing between high coverage archaic genomes. *D*-statistics among the currently available high-coverage archaic human genomes reveal population structure between early Neandertals (*D17* and *D5*) and late Neandertals (*Vi33.19*, *Chag8*). A comparison of shared derived alleles with the high-coverage Denisovan genome (*D3*) shows a slight, but non-significant, excess affinity with early Neandertals relative to late Neandertals (1.31 < |Z| < 1.97).

Table S10. *D*-statistics between high coverage archaic genomes. For each test, the table reports the test combination of individuals (ind), the *D*-statistic value (*D*), the block-jackknife standard error (SE), the Z-score (Z), the configurations of nucleotide variants (BABA, ABBA), and the number of sites analyzed (nbr_SNP_total). |Z| > 3 are considered statistically significant.

Ind1	Ind2	Ind3	Outgroup	*D*	SE	Z	BABA	ABBA	nbr_SNP_total
Nea-D5	Nea-Vi33.19	Nea-D17	S_Mbuti-1	0.1111	0.013491	8.234	46,822	37,459	19,684,614
Nea-D5	Nea-Chag8	Nea-D17	S_Mbuti-1	0.14	0.014129	9.911	47,510	35,836	19,390,001
Nea-C8	Nea-Vi33.19	Nea-D17	S_Mbuti-1	-0.0394	0.014009	-2.812	30,053	32,517	19,433,015
Nea-D17	Nea-Vi33.19	Nea-D5	S_Mbuti-1	0.1428	0.013938	10.243	46,822	35,121	19,684,614
Nea-D17	Nea-Chag8	Nea-D5	S_Mbuti-1	0.1575	0.014894	10.573	47,510	34,581	19,390,001
Nea-Chag8	Nea-Vi33.19	Nea-D5	S_Mbuti-1	-0.0238	0.013986	-1.703	30,854	32,357	19,478,564
Nea-Vi33.19	Nea-D5	Nea-Chag8	S_Mbuti-1	0.3634	0.011034	32.934	66,082	30,854	19,478,564
Nea-Vi33.19	Nea-D17	Nea-Chag8	S_Mbuti-1	0.3589	0.011494	31.225	63,707	30,053	19,433,015
Nea-D5	Nea-D17	Nea-Chag8	S_Mbuti-1	-0.0178	0.014498	-1.23	34,581	35,836	19,390,001
Nea-Chag8	Nea-D5	Nea-Vi33.19	S_Mbuti-1	0.3425	0.012265	27.927	66,082	32,357	19,47,8564
Nea-Chag8	Nea-D17	Nea-Vi33.19	S_Mbuti-1	0.3241	0.013041	24.853	63,707	32,517	19,433,015
Nea-D5	Nea-D17	Nea-Vi33.19	S_Mbuti-1	-0.0322	0.01349	-2.387	35,121	37,459	19,684,614
Nea-D5	Nea-D17	Den-D3	S_Mbuti-1	-0.0018	0.020326	-0.09	22,443	22,512	19,492,024
Nea-Vi33.19	Nea-Chag8	Den-D3	S_Mbuti-1	-0.0037	0.015364	-0.243	21,124	21,284	19,326,712
Nea-Vi33.19	Nea-D5	Den-D3	S_Mbuti-1	-0.0283	0.014379	-1.97	28,467	30,122	19,578,085
Nea-Chag8	Nea-D5	Den-D3	S_Mbuti-1	-0.0267	0.015592	-1.713	28,266	29,810	19,371,846
Nea-Vi33.19	Nea-D17	Den-D3	S_Mbuti-1	-0.0289	0.019634	-1.473	27,539	29,164	19,491,837
Nea-C8	Nea-D17	Den-D3	S_Mbuti-1	-0.0257	0.019627	-1.31	27,113	28,529	19,231,548

SI Appendix 8 – Relatedness between Neandertals *D17* and *D5*

Divyaratan Popli

Neandertals *D17* and *D5* were both recovered from the same site; however, because *D17*'s precise location in the stratigraphy is uncertain despite being reported as part of the assemblage of layer 12, it remains unclear whether they originate from the same archaeological context. Branch-length shortening analysis suggests that *D17* (100ka–120ka) may be younger than *D5* (108ka–129ka) (SI Appendix 6). However, the age ranges overlap by approximately 12ka (about 57–58% of each interval), indicating they could have coexisted, and potentially been related.

To test whether *D17* and *D5* are related, we investigate whether they share genomic tracts inherited from recent common ancestors, also called Identity-by-descent (IBD) segment. This allows the degree of kinship between two genomes to be quantified, providing direct evidence of whether they belonged to the same population group and potentially coexisted (36).

Additionally, to compare our result to a baseline expectation of unrelated and second-degree (grandparent-grandchild) related genomes, we performed coalescent simulations using msprime (37, 38). We modeled a Wright-Fischer population constant size 10,000 and generated for each individual 22 chromosomes of length 96 MB (similar to the size of the human chromosome 13), a uniform recombination rate of $1x10^{-8}$ per base pair per generation, and a mutation rate of $1x10^{-8}$ per base pair per generation under an infinite-sites model (every mutation hits a new site). From this pool of unrelated genomes, we simulate two generations of mating to generate pairs of genomes representing grandparent-grandchild relationship using Ped-sim (39). Ped-sim simulates crossovers on each chromosome based on empirical, sex-specific recombination maps and accounts for crossover interference, thereby producing realistic IBD tract distributions for second degree relatives.

Local genomic divergence between Neandertals *D17* and *D5*

To assess local genomic divergence between *D17* and *D5*, we partitioned their genomes into 10 Mb windows and computed the proportion of nucleotide differences in each window. To see how patterns of divergence in pair of presumably unrelated individuals look we repeated the same analysis for *D17* and *Chag8*, as the latter lived at least 12ky later than *D17,* on the outer bounds of their age estimates, and belonged to a distinct population. Additionally, to validate that our window size and filtering criteria are sensitive enough to distinguish relatedness from background noise, we perform the same analysis on both a simulated pair of unrelated individuals and a pair of simulated second-degree relatives.

After excluding windows with less than 3,000 overlapping sites, we found that the genome-wide average difference between *D17* and *D5* is of 0.00030 differences per site, slightly lower than the 0.00032 observed between *D17* and *Chag8.* Plotting the window-by-window average divergence for each genome pair (Fig. S16) reveals similar pattern of nucleotide-differences for *D17/D5* and *D17/Chag8*, suggesting that *D17* and *D5* are unlikely to be related. This is reinforced by the analogous analysis of simulated data for

unrelated pair of individuals and a second-degree relative pair (Fig. S17). Additionally, we observed a high correlation across windows between *D17/D5* and *D17/Chag8*, indicating that regions of reduced divergence between *D17* and *D5* are more likely due to low local genomic diversity than to recent identity-by-descent or close relatedness (Fig. S18).

Variance in windowed divergence between *D17* and *D5*

Next, we compared the window-to-window variance in mean-centered divergence for the two genome pairs, *D17/D5* and *D17/Chag8*, as well as for the simulated pairs of unrelated individuals and second-degree relative. Related individuals share long IBD tracts, causing windows to show reduced nucleotide divergence and inflating variance across the genome. In contrast, unrelated pairs exhibit more uniform divergence and thus lower variance.

The variance distribution between *D17/D5* and *D17/Chag8* are similar (Fig. S19), with an average of 5.06×10^{-9} for the *D17/D5* pair and 7.11×10^{-9} for the *D17/Chag8* pair. The variance distributions of the simulated pair of unrelated and second-degree relatives are clearly distinct (Fig. S20), with variance estimated at 3.01×10^{-5} for unrelated pairs and 2.2×10^{-4} for second-degree relatives.

In conclusion, the genomic divergence between the Neandertals *D17* and *D5* is consistent with them being unrelated at least to the second degree, and the variance of the mean-centered divergence rules out long IBD tract sharing. Thus, despite being found at the same location, with overlapping age estimates, the genomes of *D5* and *D17* Neandertals do not exhibit genomic signatures of kinship. Their shared derived alleles therefore reflect broader population history and local diversity patterns rather than recent relatedness.

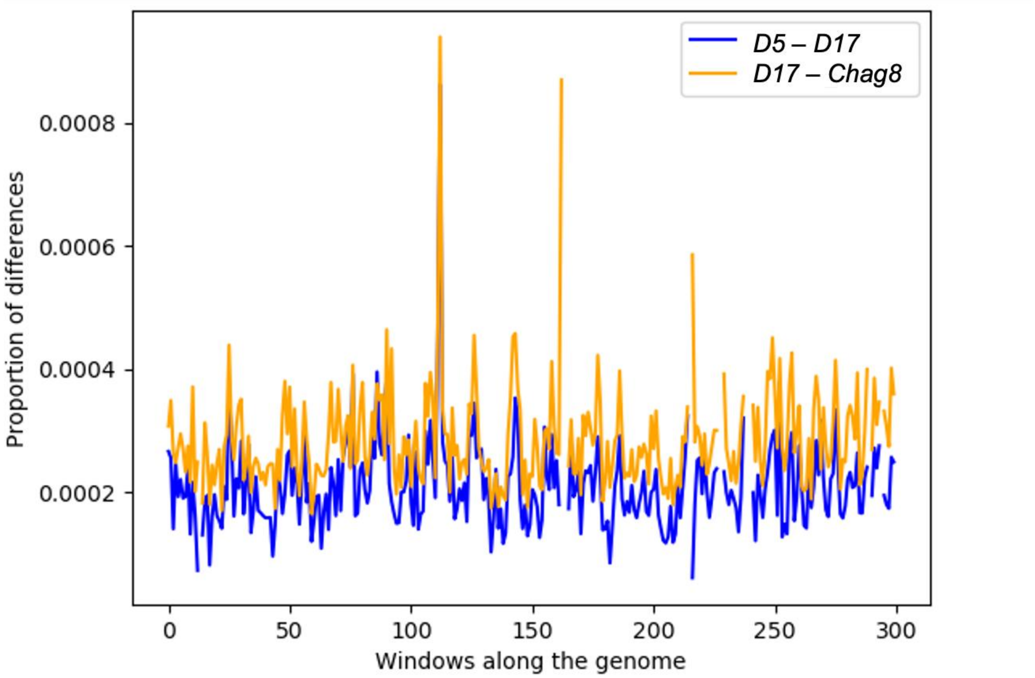

Figure S16. Proportion of differences in genomic windows between Neandertals *D17* and *D5* (blue) and between *D17* and *Chag8* (orange).

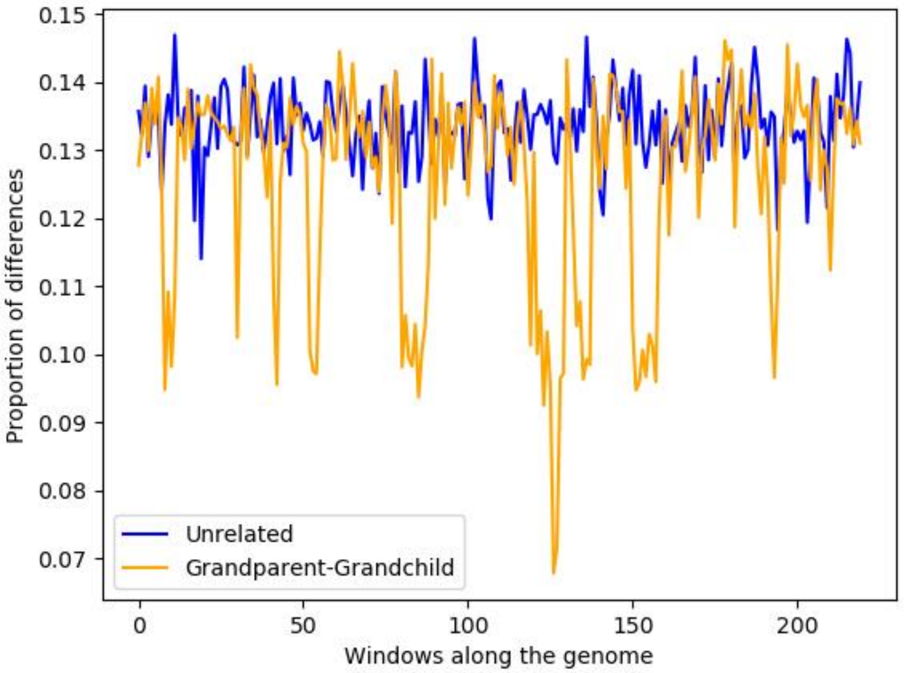

Figure S17. Proportion of differences in genomic windows between simulated pair of unrelated individuals (blue) and second-degree relatives (grandparent-grandchild) (orange).

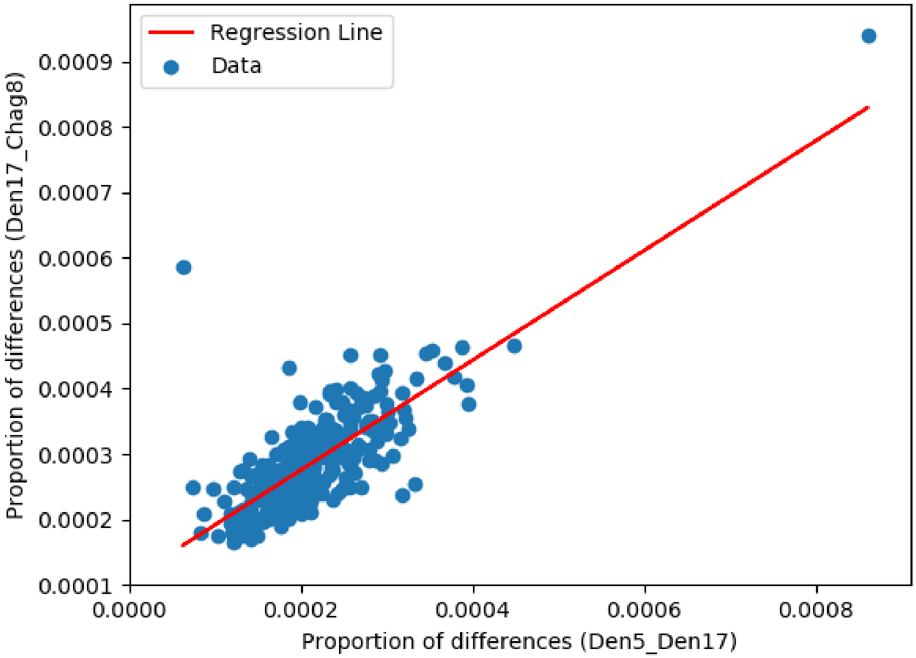

Figure S18. Correlation between the proportion of differences in genomic windows between *D17* and *D5* (x-axis) and between *D17* and *Chag8* (y-axis).

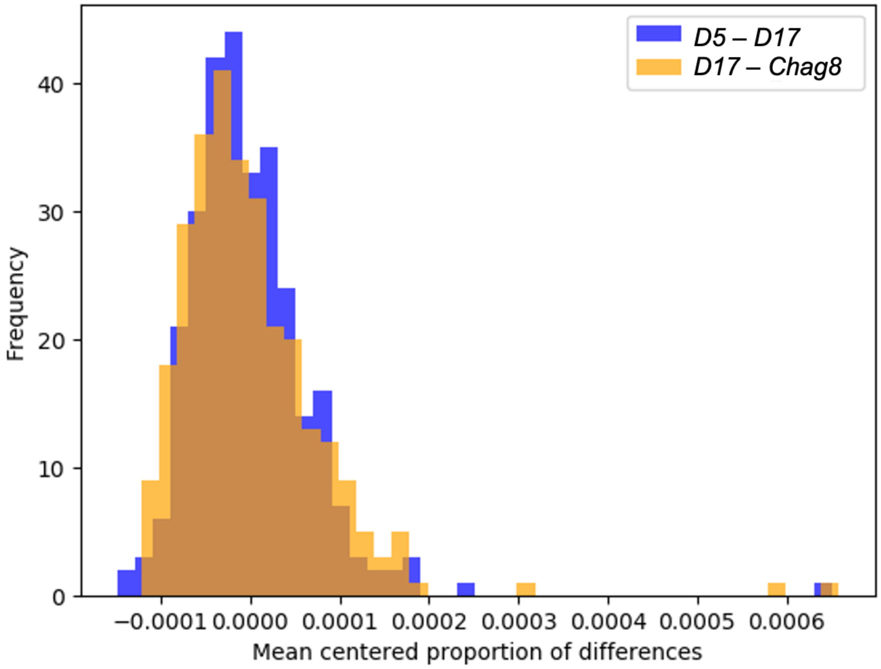

Figure S19. Histogram of mean-centered proportion of differences in genomic windows between *D17* and *D5* (blue) and between *D17* and *Chag8* (orange).

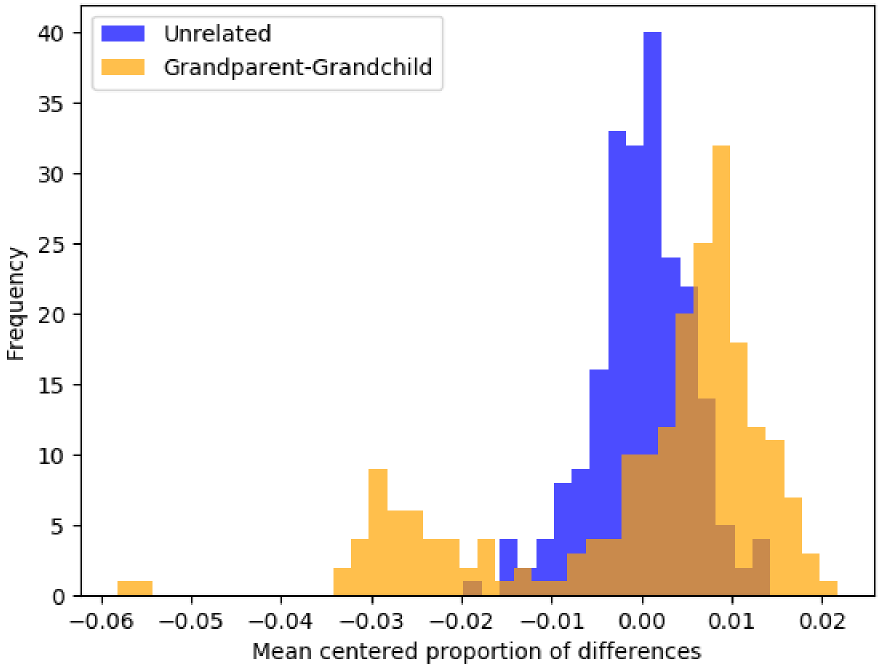

Figure S20. Histogram of mean-centered proportion of differences in genomic windows between simulated pair of unrelated individuals (blue) and second-degree relatives (grandparent-grandchild) (orange).

SI Appendix 9 – Heterozygosity and Inbreeding

Cesare de Filippo

We investigated the degree of isolation and social structure of the population represented by the newly sequenced Neandertal *D17*, by analyzing genome wide heterozygosity and runs of homozygosity, which revealed patterns of genetic diversity and inbreeding.

Heterozygosity in the *D17* genome

We calculated heterozygosity as the number of heterozygous sites divided by the total number of base-pairs that passed the minimum set of filters (SI Appendix 3), without applying any filter on genotype quality. Heterozygosity is expressed as the number of heterozygous sites per 10,000 base-pairs (Table S11 and Fig. S21) (8, 18, 25). *D17* shows the lowest heterozygosity (1.28) among the high-coverage archaic genomes. Even after excluding regions of the genome affected by inbreeding, its heterozygosity remains the lowest (1.67) (Table S11). In contrast, *D5* has the highest heterozygosity (2.15) among archaic individuals after similar filtering, albeit showing inbreeding levels comparable to those of *D17* (Table S11).

Homozygosity in the *D17* genome

We applied a previously described method (8, 18) to detect regions that are homozygosity by descent (HBD) due to inbreeding. Briefly, the method initially identifies continuous runs of homozygosity containing no heterozygous sites (ROHs), which are subsequently grouped into HBD segments, allowing for a specified proportion of heterozygous sites. This accounts for potential disruptions in the ROHs caused by genotyping errors and/or rare, *bona fide* new mutations. The proportion of the HBD tract that strictly consists of ROHs is defined as parameter π, which is specific to each genome, since errors and heterozygosity can differ between genomes.

To identify the optimal π, we look at the amount of HBD segments for different values of $\pi = \{0.8, 0.825, \ldots, 0.975, 0.99\}$. We posit that reducing π (or increasing errors) will cause adjacent short segments to merge into longer tracts, leading to a plateau or marginal increase once most errors are accounted for. The cutoff is selected as the π value that yields the highest proportion of long HBD segments relative to the total amount of intermediate and long HBD segments. This approach maximizes the detection of long segments while minimizing the detection of shorter ones, since lowering π could result in more false positives for shorter segments (Fig. S22). We also use the minimum and maximum π values as a measure of the variation in HBD estimates, providing an indication of their sensitivity to the chosen cutoff.

We computed the total number of sites within HBD segments divided by the total number of sites retained after filtering (Table S12). Alongside a constant recombination rate of 1.3cM/Mb, we explored the impact of employing the African Americans and European "deCODE" recombination maps to retrieve the genetic length (40, 41). Employing these methods for determining genetic length, we categorized regions into intermediate length if they are between 2.5 and 10 cM and long length if they are longer than 10 cM.

D17 and *D5* have comparable amounts and numbers of long HBD segments, although *D5* has the longest tract (Table S12). These values fall within the range expected under recent inbreeding scenarios, from first cousins to double first cousins (18). The Neandertals *Chag8* and *Vi33.19* show slightly but significantly lower values for all statistics associated with HBD segments longer than 10 cM. Regarding the intermediate HBD segments *D17*, *Chag8*, and *Vi33.19* shows similar values for the amount (~12%) and number of segments (~120), while *D5* has lower levels (~7% and 70 segments) (Table S12, Figure S22). The amounts of intermediate length segments in *D17*, *Chag8* and *Vi33.19* are much higher than expected under inbreeding scenarios ranging from first cousins to double first cousins, which would account for at most 6-8% (18). It suggests that these Neandertals experienced a lower long-term effective population size (or higher number of repeated inbreeding events) than *D5* (Table S11 and Figure S21). Similar patterns are observed when using the African Americans and European “deCODE” recombination maps to rescale the length of the HBD segments instead of the average recombination rate (Fig. S23, S24 and S25).

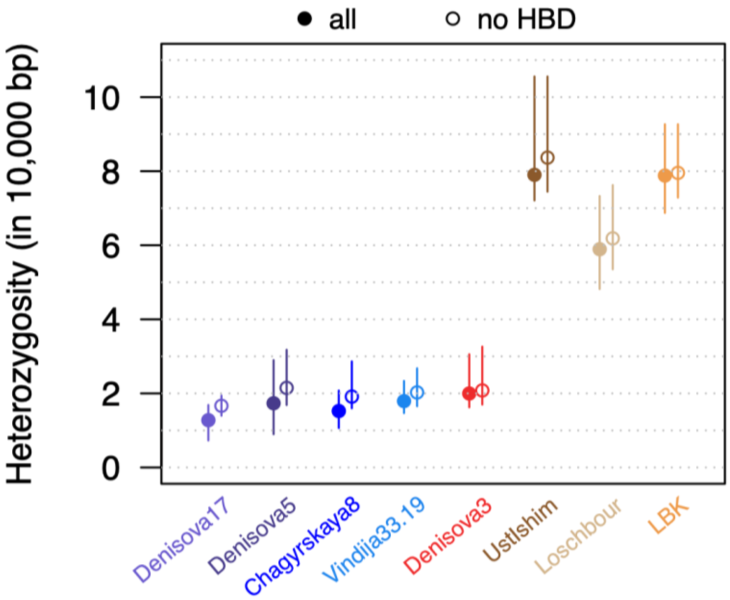

Figure S21: Average number of heterozygous sites per 10,000 bp. The vertical lines encompass the variation across the 22 autosomal chromosomes. Neandertals *D17* (Denisova17), *D5* Denisova5), Chag8 (Chagyskaya8), Vi33.19 (Vindija33.19); Denisovan D3 (Denisova3); Modern human UstIshim, Loschbour, Stuttgart LBK.

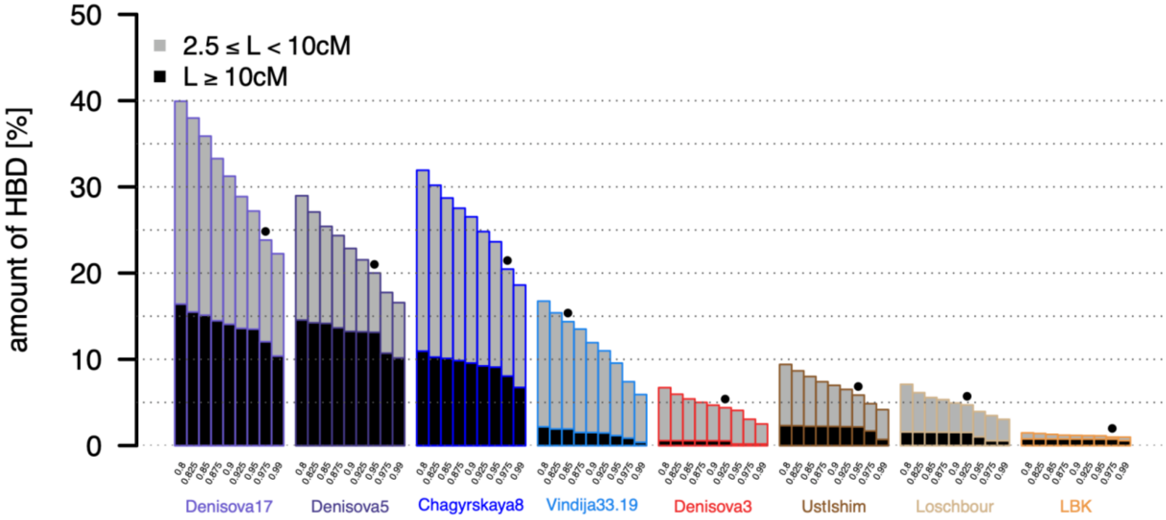

Figure S22: **Amount of HBD segments with different π parameters using average recombination rate.** The y-axis represents the percentage of HBD, while the x-axis displays various values of π for high-coverage ancient genomes. The filled black and gray bars indicate HBD segments with long and intermediate lengths (L), respectively, as indicated in the legend. The circle positioned above the bars signifies the optimal values of π for each genome, as detailed in the text. Neandertals *D17* (Denisova17), *D5* (Denisova5), Chag8 (Chagyskaya8), Vi33.19 (Vindija33.19); Denisovan D3 (Denisova3); Modern human UstIshim, Loschbour, Stuttgart LBK.

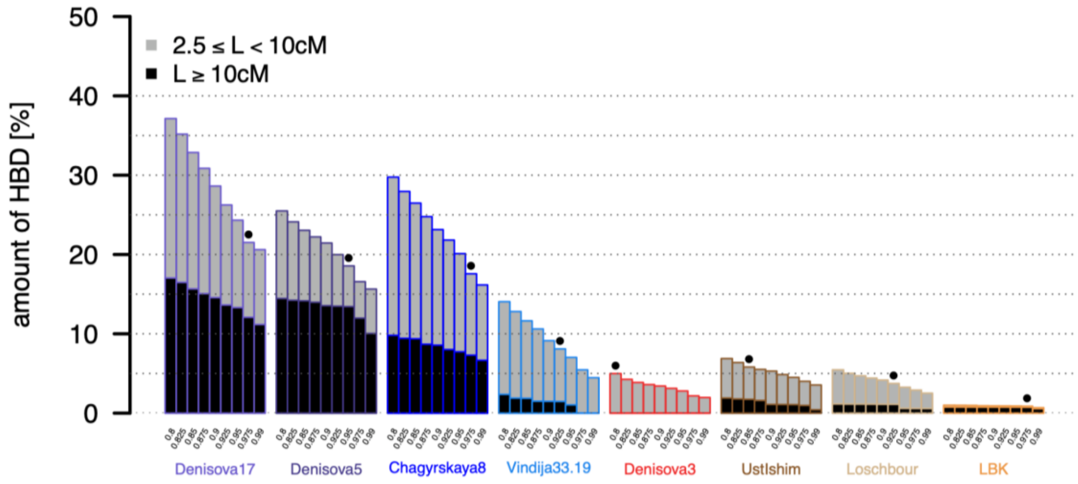

Figure S23: Amount of HBD segments with different π parameters as in Figure S22 but using the African American recombination map. Neandertals *D17* (Denisova17), *D5* (Denisova5), Chag8 (Chagyskaya8), Vi33.19 (Vindija33.19); Denisovan D3 (Denisova3); Modern human UstIshim, Loschbour, Stuttgart LBK.

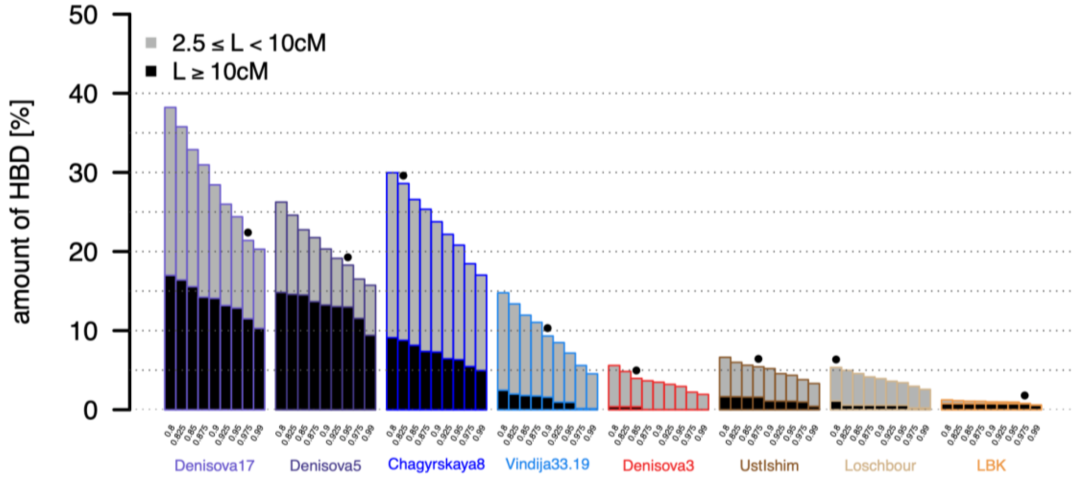

Figure S24: Amount of HBD segments with different π parameters as in Figure S22 and S23 but using the European deCode recombination map. Neandertals *D17* (Denisova17), *D5* (Denisova5), Chag8 (Chagyskaya8), Vi33.19 (Vindija33.19); Denisovan D3 (Denisova3); Modern human UstIshim, Loschbour, Stuttgart LBK.

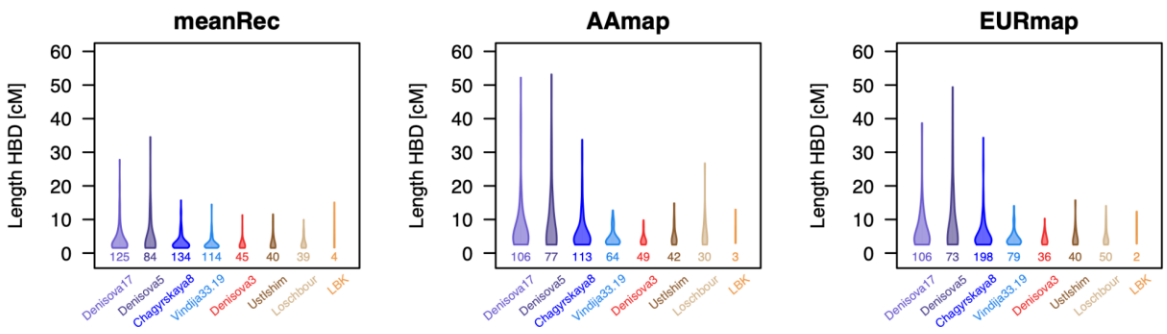

Figure S25: Distribution of the length of the HBD segments according to different recombination rates. Neandertals *D17* (Denisova17), *D5* (Denisova5), Chag8 (Chagyskaya8), Vi33.19 (Vindija33.19); Denisovan D3 (Denisova3); Modern human UstIshim, Loschbour, Stuttgart LBK.

Table S11: Heterozygosity in the archaic genomes. The numbers in parenthesis are the range of heterozygosity among the 22 autosomes.

Sample	Heterozygous sites in 10,000 bp	Heterozygous sites in 10,000 bp excluding HBD
Neandertal *D17*	1.28 (0.73-1.69)	1.67 (1.4-1.94)
Neandertal *D5*	1.73 (0.9-2.9)	2.15 (1.69-3.18)
Neandertal *Chag8*	1.52 (1.07-2.08)	1.91 (1.6-2.86)
Neandertal *Vi33.19*	1.79 (1.47-2.34)	2.03 (1.65-2.68)
Denisovan *D3*	2 (1.62-3.06)	2.08 (1.7-3.26)
UstIshim	7.9 (7.21-10.56)	8.37 (7.45-10.56)
Loschbour	5.89 (4.82-7.33)	6.19 (5.35-7.62)
Stuttgart LBK	7.88 (6.87-9.27)	7.95 (7.29-9.27)

Table S12. Amount and number of HBD segments and length of the longest HBD. The table gives the estimates under the optimal π specific for each sample (Fig. S22) and their variation using the minimum and maximum values of π (i.e., 0.8 and 0.99).

Samples	Amount long HBD	Amount all HBD	Longest HBD	Number of long HBD	Number of all HBD
Neandertal *D17*	12% (10.4-16.4)	23.8% (22.2-39.9)	27.8 cM (27.8-30.5)	10 (9-14)	125 (59-126)
Neandertal *D5*	13.1% (10.2-14.6)	20% (16.6-29)	34.5 cM (23.5-35)	14 (8-15)	70 (42-84)
Neandertal *Chag8*	8.1% (6.7-10.9)	20.5% (18.6-31.9)	15.7 cM (15.7-15.9)	5 (4-7)	119 (59-134)
Neandertal *Vi33.19*	1.9% (0.4-2.2)	14.4% (5.9-16.7)	14.5 cM (7.5-14.6)	1 (0-1)	114 (18-149)
Denisovan *D3*	0.5% (0.2-0.6)	4.4% (2.5-6.7)	11.3 cM (11.2-11.5)	1 (1-1)	29 (9-45)
UstIshim	2.2% (0.7-2.3)	5.9% (4.2-9.4)	11.5 cM (8.1-12.6)	1 (0-2)	26 (14-40)
Loschbour	1.5% (0.6-1.6)	4.7% (3-7.1)	9.9 cM (6.7-10.2)	0 (0-1)	25 (12-39)
LBK	0.7% (0.6-0.8)	1% (1-1.5)	15.1 cM (11.3-15.5)	1 (1-1)	4 (2-6)

SI Appendix 10 – Estimates of split time between *D17* and other archaic genomes using *cecast*

Cesare de Filippo, Diyendo Massilani

We used *cecast* (https://github.com/cesaredef/cecast/tree/main) to estimate the split time of *D17* relative to Neandertals *D5 (18)*, *Chag8 (25)* and *Vi33.19 (8)*. The method calculates the likelihood of observed combinations of ancestral and derived alleles across genomes (the sites patterns, *i.e*., lineage assignment proportions SI Appendix 4) and modern human contamination under specified demographic parameters (see section Contamination SI Appendix 5).

Analyses were performed using *mcecast, a* version of *cecast* that jointly fits demographic parameters in real time, including modern human contamination. In constrast to the default *cecast* implementation, which estimates only split times and branching relationships while keeping the other parameters of the demography of modern humans, Neandertals, and Denisovans fixed and estimates confidence intervals by bootstrapping the demographic parameters over reasonable ranges, *mcecast* simultaneously estimates all parameters together.

We ran the program using as input the lineage assignment results of the *D17* genome against five high coverage genomes: modern human Mbuti, Denisovan *D3*, and Neandertals *D5*, *Chag8* and *Vi33.19* (SI Appendix 4). The results estimate the *D17* split from the *D5* around 131ka (112-151ka) (Table S13 and S14). The results show that *D17* branches from the *D5* across all the top 100 simulations (encompassing > 99% of the confidence interval). The split between *D17* and *D5* is estimated to have occurred on average 8,640 years (range:3,801-14,867) after the common ancestors of both *D17* and *D5* diverged from the common ancestors of the Neandertals *Chag8* and *Vi33.19,* and approximately 14,949 years (range:1,548-37,249 years) before the death of the *D5* individual (Fig. S26). Looking at the top 1,000 *mcecast* simulations, 4.5% and 2.4% indicate that *D17* branches from the common ancestors of all Neandertals, suggesting trifurcation. However, the likelihoods of these simulations are substantially lower than those of the best-fitting models (Figure S26).

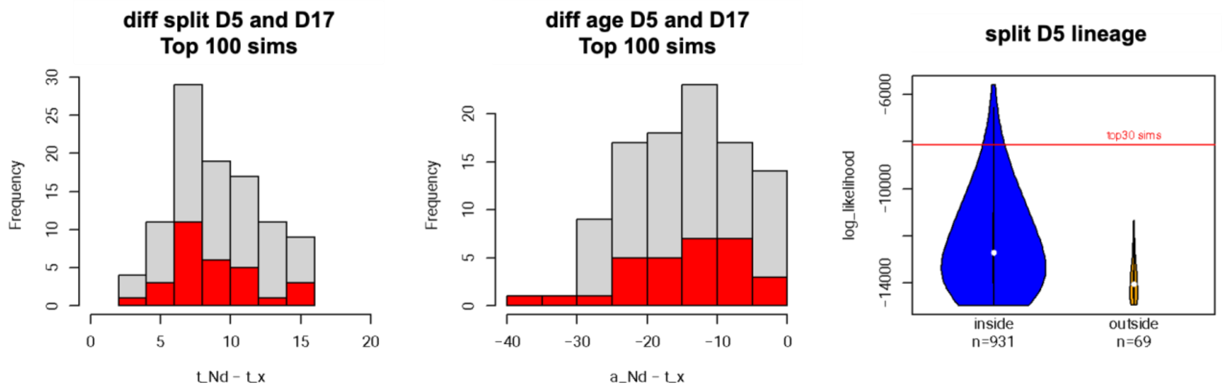

Figure S26. *Cecast* distributions of posteriors estimates. The left plot shows the differences between the split time of *D5* and the split time of *D17* (t_Nd – t_x) and the central plot shows the differences between the age of *D5* and the split time of *D17* (a_Nd – t_x) on the best 100 simulations (99.6% CIs). In both plots, the top 30 simulations (red), used to derive the standard 95% CIs in Table S13 and S14. The right (violin) plot shows the likelihood distributions for the top 1,000 simulations with alternative branching topologies for *D17*: in blue, splits occurring from or within the *D5* lineage; in orange, split occurring outside the *Denisova 5* lineage. Diff = difference, Sims = Simulations.

Table S13. Split time of *D17* and contamination estimates using two version of *cecast*.

Method	Split time (t_x)	Split pop	Contamination (c)	logLike	N sites
mcecast	130 (112-151) kya	*D5*	0.3 (0-1) %	-7,193	2,035,328

Table S14. Demographic parameters estimate in *mcecast.*

Parameter	estimate	min	max	unit	Description
t_x	130	112	151	kya	split time of the sample "*D17"* and its closest relative "*D5*"
t_HND	626	550	692	kya	split time between modern and archaic humans
t_ND	446	395	499	kya	split time between Neandertals and Denisovans
t_iH	416	311	500	kya	split time of introgressing Humans
t_sA	1460	719	2441	kya	split time of super-Archaic population
tm_HN	295	209	421	kya	time of modern Human to Neandertal gene-flow
tm_SD	327	154	471	kya	time of super-Archaic to Denisovan gene-flow
tm_ND	259	139	345	kya	time of Neandertal to Denisovan gene-flow
m_HN	8.9	1.1	14.7	%	amount of modern human to Neandertal gene-flow
m_SD	3.6	0.1	9.4	%	amount of super-Archaic to Denisovan gene-flow
m_ND	2.8	0.6	5.0	%	amount of Neandertal to Denisovan gene-flow
c	0.3	0.0	0.8	%	amount of modern human contamination

SI Appendix 11 – Archaic human effective population sizes inferred by PSMC

Arev Pelin Sümer, Alba Bossoms Mesa, Stéphane Peyrégne

We used the Pairwise Sequentially Markovian Coalescent (PSMC) (42) to investigate past effective population sizes. This provides a timeline of population history and insights into when groups split and how they remained connected through shared ancestry. PSMC infers these patterns by modeling the temporal distribution of coalescent events within a hidden Markov framework, allowing the reconstruction of demographic history from a single diploid genome. Alongside the *D17* genome, we analyzed the previously published archaic genomes of the Neandertals *D5* (18), *Vi33.19 (8)* and *Chag8* (25) and the Denisovan *D3* (10). These genomes were genotyped using snpAD (v.0.3.11) (15) and data were filtered using standard filters as detailed in SI Appendix 3. The ages of *D17* and *D5* were estimated using demographic curves inferred by PSMC and the previously estimated molecular age estimate for *Vi33.19* (8).

Inference of population size changes over time

We converted the snpAD (v.0.3.11) genotype calls for each ancient genome into psmcfa format, which is the input file format of PSMC. To do so we divided each genome into windows of 100bp, and determined for each window whether at least one heterozygous site was present (state K), whether all called sites were homozygous (state T), or more than 10% of the data was missing (state N). We then merged the data from all autosomes for each individual and ran PSMC (version 0.6.5). Due to the sensitivity of PSMC to missing data caused by filtering (10, 25), we followed the approach in Mafessoni *et al.*, 2020 (25) with the modifications of Sümer *et al.*, 2024 (43) to correct the inferred demographic history given the missing data. We did this by calibrating our parameters θ (mutation rate) and ρ (recombination rate) using simulations with scrm (44), and following the steps listed in Sümer *et al.*, 2024 (43). The pipeline used for this analysis is available through https://github.com/StephanePeyregne/calibratePSMC. We calculated the likelihood for each simulated combination of correction factors and identified the pair with the maximum likelihood. These were used to obtain the corrected demographic history (Fig. S27, S28, Table S15).

The inferred that the demographic histories of the four high coverage Neandertals were very similar to one another and different from the Denisovan *D3*, as shown previously (8, 25). *D5* and *D17* had almost identical curves between ~800,000 years BP and the death of the individuals, except for a small increase in *Ne* in the recent history of the *D5* Neandertal. To understand if this represents a genuine difference in the demographic histories of the two Neandertals, or noise caused by the uncertainty of PSMC curves close to the individuals' lifetime, we ran 50 bootstraps for the calibrated demography of *D5* and *D17* (with both the standard and modified filters) independently (Fig. S29). Most bootstraps do overlap at this bump ~140,000 years BP. Since other analyses (SI Appendix 4, 10, 12 and 13) indicate that *D17* falls on the lineage leading to *D5*, and should therefore have the same demographic history, and because this increase

in *Ne* is not observed when only neutrally evolving sites are used to infer the demographies (Fig. S30), we conclude that the recent increase inferred for *D5* is due to inaccuracy of PSMC.

Considering the effect of background selection

Since background selection influences the shape of the demographic curves and may therefore bias inferences (45, 46) we removed all regions with evidence of a reduction in genetic variation due to background selection following Sümer *et al*., 2024 (43). After removing regions with a *B*-score less than 900 (47), we repeated the calibration of the θ (mutation rate) and ρ (recombination rate) parameters. Results show similar patterns to those observed above (Table S17, Fig. S30).

Age estimates using demographic histories

We estimated the ages of *D17* and *D5* using the demographic histories inferred by PSMC, following the approach in (25) where we compared the demographic history reconstructed from the genome of *Vi33.19*, a Neandertal that lived around 45,000-50,000 years ago, and those of *D17* and *D5* to estimate the age difference between these individuals. For this analysis, we used 50,000 years as the age for *Vi33.19*, as the previous estimated (8).

Following the pipeline described (43) we first truncated the corrected demographic history of the younger individual, the *Vi33.19* Neandertal, obtained previously (Table S15), by 0 to 162,429 years in steps of 5,075 years. We simulated ten whole genomes each time, following that truncated demographic history, and filtered these with the filters applied to the genome of either the *D17* or the *D5* genomes. We obtained new demographic histories from these simulated and filtered genomes, by running PSMC, and evaluated the fit of this demography to the genome of *D17* and *D5*, independently. This was done by computing the likelihood with the command "psmc -N1 -i {input.demography} -o {output} {input.data}", as in previous studies (25, 43). Finally, we estimated the best fitting age for *Vi33.19* and *D17* or *D5*, by identifying the best fitting truncated demographic history of *Vi33.19* to the genome of the other two. Our estimates from the simulations are plotted in (Fig. S31) where the intervals between the dashed lines corresponding to the top 5% simulations with the highest likelihood.

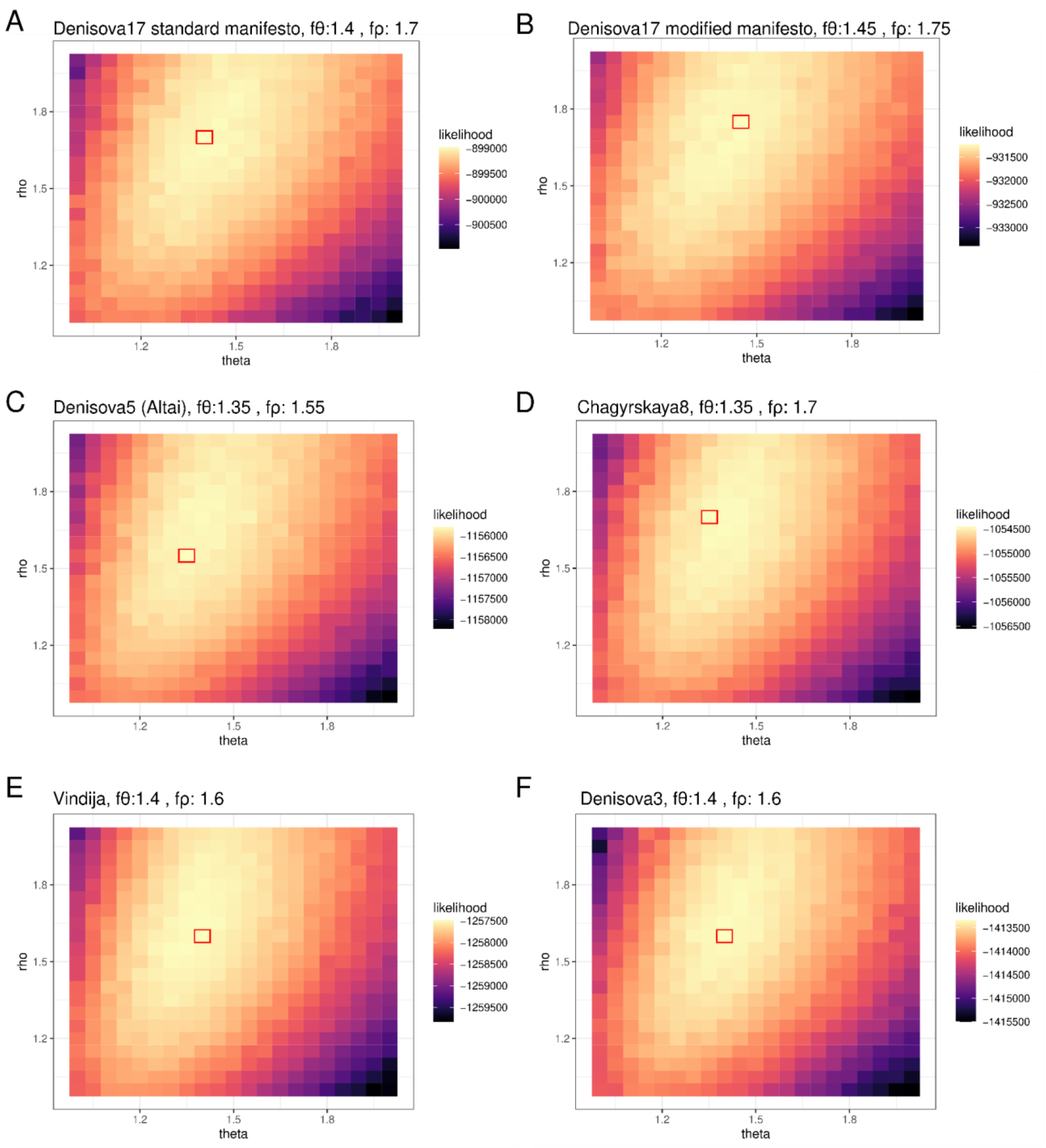

Figure S27. Fit of the corrected demographic histories for each pair of correction factors of the θ and ρ parameters (fθ and fρ). Likelihood of the real data given different correction factors, fθ and fρ, is colour coded. The best pair of correction factors for each genome is indicated with a red square and corresponds to the likelihood values reported in Table S15.

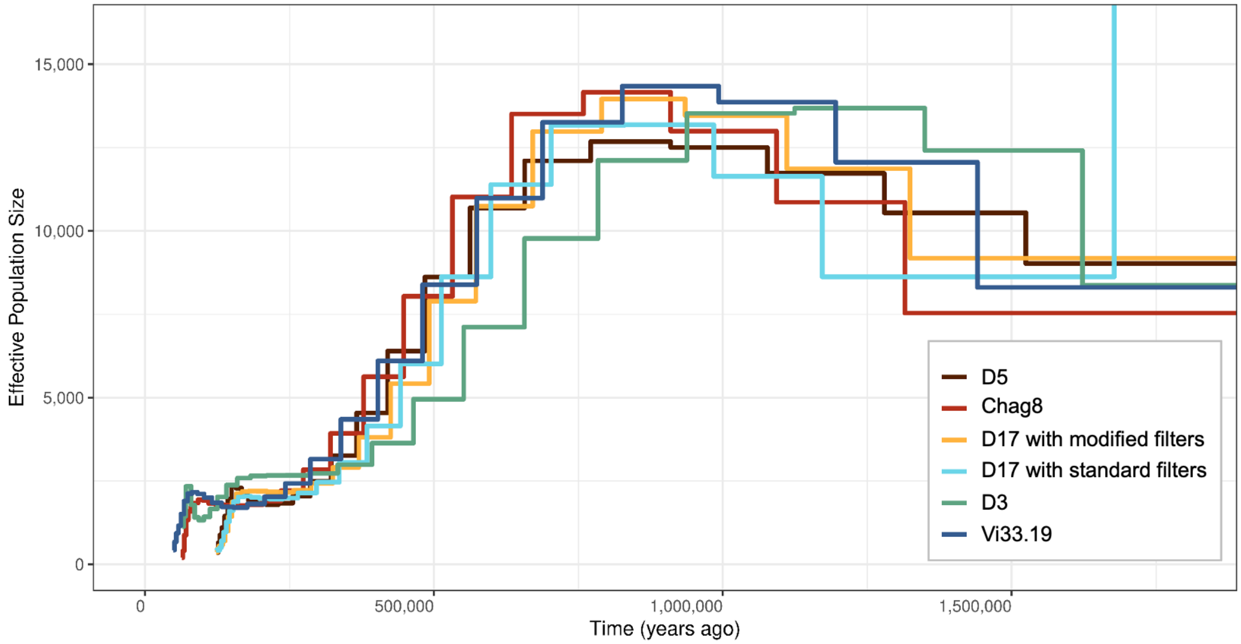

Figure S28. Corrected demographies using the factors reported in Table S15, assuming a mutation rate of 0.5×10^{-9} per year per base pair - or 1.45×10^{-8} per generation per base pair, for a generation time of 29 years. The curves are aligned by the age of each specimen: 50,000 years for *Vi33.19*; 120,000 years for *D17* and *D5*; and 80,000 for *Chag8* and the Denisovan *D3*.

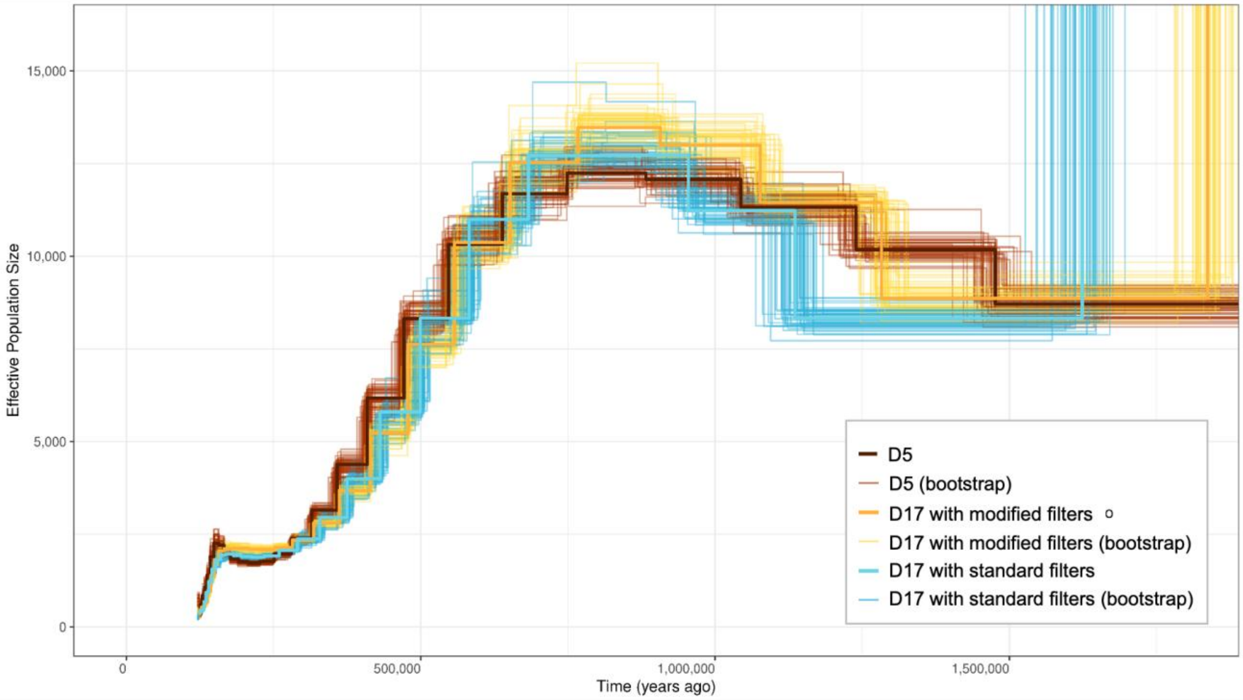

Figure S29. Corrected demographic curves for *D5*, *D17* with standard manifesto filters and *D17* with modified manifesto filters (SI Appendix 3), with 50 bootstraps for each. The curves are aligned by assuming 120,000 years of age for *D17* and *D5*, a mutation rate of 0.5×10^{-9} per year per base pair, a generation time of 29 years. We use the calibration factors reported in Table S15. Thick lines are the demographies plotted in Figure S28 while the thin ones are the bootstraps.

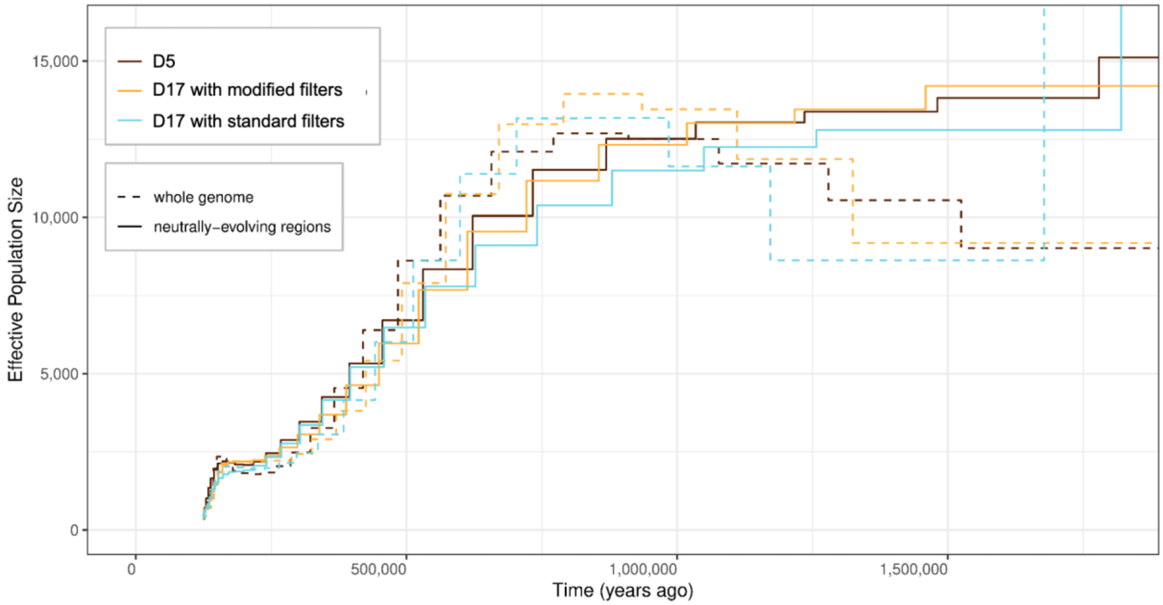

Figure S30. Demographies for *D17* and *D5* ("Altai") corrected for the effect of background selection and missing data. Factors are reported in Table S17. Dashed lines indicate demographic curves obtained using whole genomes, and solid lines only the genomic regions that presumably evolve neutrally.

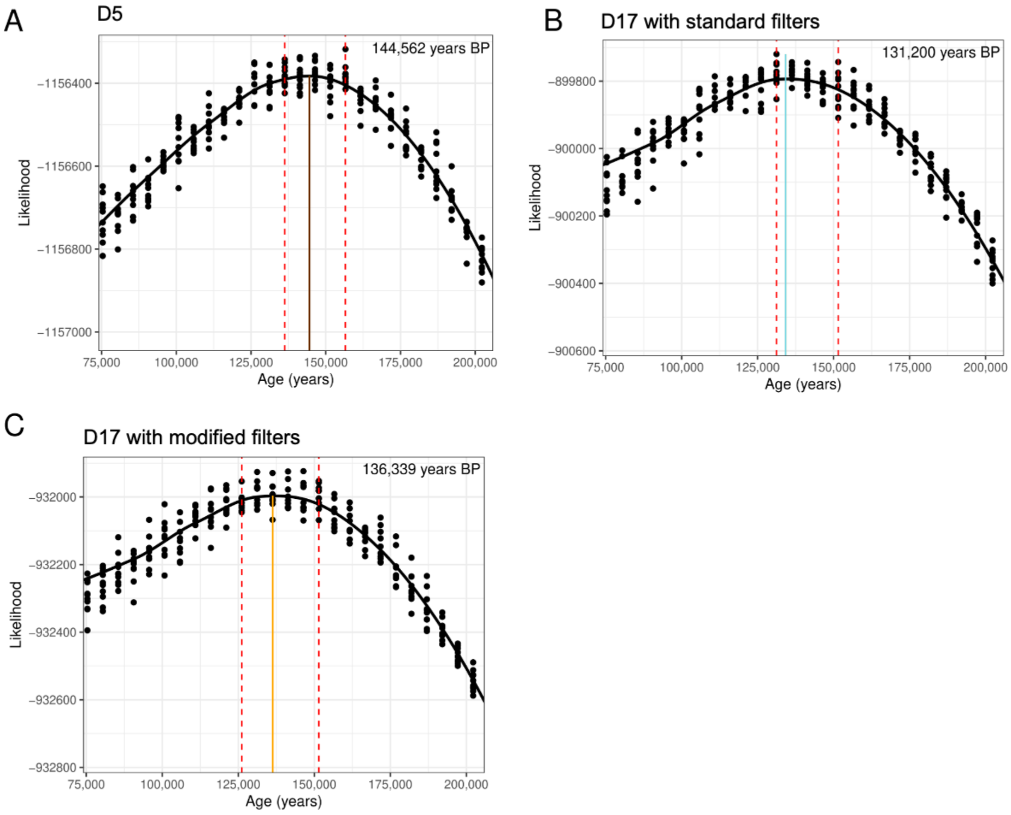

Figure S31. Age estimates for (**A**) *D5*; (**B**) *D17* with standard manifesto filters; (**C**) *D17* with modified manifesto filters, assuming *Vi33.19* is 50,000 years old. Intervals in red dashed lines indicate estimates from simulations with the best likelihood (top 5%), and the solid lines indicate the peak of the distribution, also reported on the top right in each plot.

Table S15 - Correction factors of the θ and ρ parameters ($f\theta$ and $f\rho$) for the genomes included in the PSMC analysis. Demographies inferred for San (*SS6004473*, *HGDP01036*)(8), Mbuti (*SS6004471*, *HGDP00982*)(8) and French (*SS6004468*, *HGDP00533*)(8) were used for the split time estimate analyses.

Genome	f_Theta	f_Rho	Likelihood
Neandertal D17 standard manifesto	1.4	1.7	-898,9767
Neandertal D17 modified manifesto	1.45	1.75	-931,230
Neandertal Chag8	1.35	1.7	-1,054,455
Neandertal D5	1.35	1.55	-1,155,793
Neandertal Vi33.19	1.4	1.6	-1,257,487
Denisovan D3	1.4	1.6	-1,413,338
Mbuti	1.3	1.2	-5,150,129
San	1.3	1.15	-5,212,127

Table S16. scrm parameters to produce simulations of the corrected demographic histories plotted in Fig. S29.

Genome	Simulation code
D17 Standard manifesto	scrm 2 1 -SC abs -p 10 -t 43560.9 -r 11748.5 2881033286 -en 0.1065 1 2.4254 -en 0.1775 1 2.0682 -en 0.2639 1 1.9697 -en 0.3688 1 2.3202 -en 0.4962 1 3.2487 -en 0.6512 1 4.8125 -en 0.8395 1 6.6519 -en 1.0684 1 8.0534 -en 1.3466 1 9.1759 -en 1.6846 1 9.9249 -en 2.0955 1 9.9905 -en 2.5949 1 9.6644 -en 3.2017 1 9.3500 -en 3.9393 1 9.3625 -en 4.8358 1 9.8180 -en 5.9253 1 10.6367 -en 7.2494 1 11.9997 -en 8.8587 1 14.6651 -en 10.8145 1 20.2007 -en 13.1915 1 29.9289 -en 16.0805 1 42.7828 -en 19.5916 1 55.2741 -en 23.8588 1 63.6304 -en 29.0450 1 65.0699 -en 35.3480 1 58.7833 -en 43.0085 1 42.9414 -en 63.6338 1 112.4672
D17 Modified manifesto	scrm 2 1 -SC abs -p 10 -t 66714.8 -r 17026.8 2881033286 -en 0.0762 1 1.2033 -en 0.1270 1 1.4567 -en 0.1888 1 1.5416 -en 0.2637 1 1.6286 -en 0.3548 1 2.1587 -en 0.4655 1 3.2437 -en 0.5999 1 4.6346 -en 0.7632 1 5.7698 -en 0.9616 1 6.6129 -en 1.2026 1 6.9953 -en 1.4954 1 6.8427 -en 1.8511 1 6.6011 -en 2.2833 1 6.4964 -en 2.8083 1 6.6045 -en 3.4460 1 6.9537 -en 4.2208 1 7.5336 -en 5.1621 1 8.5225 -en 6.3056 1 10.4566 -en 7.6947 1 14.4403 -en 9.3823 1 21.4230 -en 11.4325 1 30.5924 -en 13.9232 1 39.2991 -en 16.9490 1 44.7940 -en 20.6249 1 45.4286 -en 25.0906 1 41.0746 -en 30.5157 1 30.0294 -en 45.1130 1 80.4049
D5	scrm 2 1 -SC abs -p 10 -t 103262 -r 22873.2 2881033286 -en 0.0619 1 0.7580 -en 0.1029 1 1.0464 -en 0.1528 1 1.5321 -en 0.2131 1 2.2953 -en 0.2863 1 3.1115 -en 0.3751 1 3.8357 -en 0.4826 1 4.3607 -en 0.6130 1 4.4839 -en 0.7710 1 4.2493 -en 0.9626 1 3.9807 -en 1.1949 1 3.8245 -en 1.4764 1 3.8208 -en 1.8177 1 3.9698 -en 2.2314 1 4.2343 -en 2.7328 1 4.6638 -en 3.3408 1 5.6421 -en 4.0777 1 7.9087 -en 4.9710 1 12.0439 -en 6.0539 1 17.3980 -en 7.3665 1 21.8660 -en 8.9578 1 24.2248 -en 10.8867 1 25.1204 -en 13.2250 1 25.2723 -en 16.0594 1 24.6906 -en 19.4954 1 23.0977 -en 23.6606 1 18.9418 -en 34.8302 1 64.0323
Chag8	scrm 2 1 -SC abs -p 10 -t 1.55791e+06 -r 367638 2881033286 -en 0.0035 1 0.0219 -

	en 0.0058 1 0.0565 -en 0.0087 1 0.1007 -en 0.0122 1 0.1583 -en 0.0164 1 0.2276 -en 0.0215 1 0.2789 -en 0.0277 1 0.2763 -en 0.0353 1 0.2525 -en 0.0445 1 0.2487 -en 0.0557 1 0.2554 -en 0.0693 1 0.2526 -en 0.0858 1 0.2458 -en 0.1059 1 0.2465 -en 0.1303 1 0.2551 -en 0.1601 1 0.2700 -en 0.1962 1 0.2984 -en 0.2402 1 0.3580 -en 0.2936 1 0.4871 -en 0.3586 1 0.7447 -en 0.4376 1 1.1382 -en 0.5337 1 1.5216 -en 0.6505 1 1.7214 -en 0.7925 1 1.7537 -en 0.9652 1 1.7139 -en 1.1752 1 1.5889 -en 1.4305 1 1.1200 -en 2.1185 1 4.3035
Vi33.19	scrm 2 1 -SC abs -p 10 -t 2.1522e+07 -r 4.63662e+06 2881033286 -en 0.0003 1 0.0050 -en 0.0006 1 0.0064 -en 0.0008 1 0.0106 -en 0.0011 1 0.0142 -en 0.0015 1 0.0185 -en 0.0020 1 0.0212 -en 0.0026 1 0.0214 -en 0.0033 1 0.0204 -en 0.0041 1 0.0196 -en 0.0051 1 0.0194 -en 0.0063 1 0.0187 -en 0.0078 1 0.0178 -en 0.0096 1 0.0176 -en 0.0117 1 0.0188 -en 0.0143 1 0.0213 -en 0.0174 1 0.0250 -en 0.0212 1 0.0314 -en 0.0257 1 0.0442 -en 0.0312 1 0.0660 -en 0.0379 1 0.0928 -en 0.0459 1 0.1147 -en 0.0556 1 0.1267 -en 0.0673 1 0.1313 -en 0.0815 1 0.1292 -en 0.0986 1 0.1180 -en 0.1192 1 0.0911 -en 0.1743 1 0.3358
Denisova3	scrm 2 1 -SC abs -p 10 -t 449396 -r 95244.4 2881033286 -en 0.0182 1 1.0000 -en 0.0302 1 0.7380 -en 0.0446 1 1.2575 -en 0.0622 1 1.1686 -en 0.0833 1 0.7995 -en 0.1088 1 0.6081 -en 0.1397 1 0.6177 -en 0.1770 1 0.7360 -en 0.2220 1 0.8701 -en 0.2764 1 0.9820 -en 0.3422 1 1.1030 -en 0.4215 1 1.2347 -en 0.5174 1 1.3211 -en 0.6333 1 1.3125 -en 0.7732 1 1.2470 -en 0.9423 1 1.2250 -en 1.1465 1 1.3566 -en 1.3932 1 1.7792 -en 1.6912 1 2.6299 -en 2.0512 1 3.8493 -en 2.4860 1 5.0711 -en 3.0113 1 5.9203 -en 3.6459 1 6.3911 -en 4.4125 1 6.4998 -en 5.3384 1 6.0398 -en 6.4571 1 4.3939 -en 9.4406 1 20.1741
San (SS6004473, HGDP01036)	scrm 2 1 -SC abs -p 10 -t 3.48943e+06 -r 470238 2881033286 -en 0.0164 1 0.7845 -en 0.0268 1 1.0546 -en 0.0389 1 1.1066 -en 0.0532 1 1.2858 -en 0.0699 1 1.3674 -en 0.0895 1 1.3598 -en 0.1125 1 1.3550 -en 0.1394 1 1.2566 -en 0.1709 1 1.0790 -en 0.2079 1 0.9088 -en 0.2513 1 0.7804 -en 0.3021 1 0.6895 -en 0.3616 1 0.6311 -en 0.4315 1 0.6016 -en 0.5133 1 0.5961 -en 0.6092 1 0.6119 -en 0.7217 1 0.6536 -en 0.8535 1 0.7335 -en 1.0079 1 0.8657 -en 1.1890 1 1.0476 -en 1.4013 1 1.2353 -en 1.6501 1 1.3574 -en 1.9417 1 1.3789 -en 2.2835 1 1.3344 -en 2.6842 1 1.2977 -en 3.1539 1 1.4693 -en 4.3498 1 5.0458
Mbuti (SS6004471, HGDP00982)	scrm 2 1 -SC abs -p 10 -t 3.26666e+06 -r 474753 2881033286 -en 0.0172 1 0.5575 -en 0.0280 1 0.8056 -en 0.0408 1 0.9256 -en 0.0557 1 1.1389 -en 0.0733 1 1.3670 -en 0.0938 1 1.4694 -en 0.1180 1 1.4534 -en 0.1463 1 1.3397 -en 0.1795 1 1.1608 -en 0.2184 1 0.9714 -en 0.2641 1 0.8267 -en 0.3177 1 0.7323 -en 0.3806 1 0.6739 -en 0.4544 1 0.6430 -en 0.5409 1 0.6375 -en 0.6424 1 0.6570 -en 0.7615 1 0.7043 -en 0.9012 1 0.7875 -en 1.0650 1 0.9179 -en 1.2572 1 1.0974 -en 1.4827 1 1.2915 -en 1.7472 1 1.4320 -en 2.0575 1 1.4771 -en 2.4214 1 1.4476 -en 2.8483 1 1.4089 -en 3.3491 1 1.5570 -en 4.6258 1 5.2190

Table S17. Correction factors of the θ and ρ parameters (f_θ and f_ρ) for the genomes included in this part investigating the effect of background selection.

Genome	f_Theta	f_Rho	Likelihood
Neandertal D17 Standard manifesto	1.35	1.8	-388,096
Neandertal D17 Modified manifesto	1.45	2	-402,268
Neandertal D5 (Altai)	1.4	1.9	-501,489
Neandertal Chag8	1.45	1.85	-455,580
Neandertal Vi33.19	1.4	1.9	-501,489
Denisovan D3	1.4	1.8	-591,695

Table S18. Branch shortening estimates at the peak of the distribution plotted in Fig. S31, and corresponding likelihoods. In the figure, minimum and maximum values, corresponding to the estimates from simulations with the highest likelihood (top 5%), are those indicated by the dashed lines.

Genomes used		Estimated age for the focus (G*old*) genome			
**G*old***	**G**	**Peak of distribution**	**Likelihood**	**Minimum**	**Maximum**
Neandertal D5	*Vi33.19*	144,562	-1,156,383	135,275	155,575
Neandertal D17 Standard manifesto	*Vi33.19*	131,200	-899,720	131,200	151,500
Neandertal D17 Modified manifesto	*Vi33.19*	136,339	-931,997	126,125	151,500

SI Appendix 12 – Estimation of split time using F(A|B) statistics

Alba Bossoms Mesa, Arev Pelin Sümer, Stéphane Peyrégne

We estimated the split time between *D17* and other archaic genomes making use of the F(A|B) statistic (18, 25, 48), which measure the proportion of the sites that are heterozygous in the genome "B" that carry the derived allele in genome "A". This proportion will be higher the more recent the split time is. The standard deviations of the inferred f(A|B) values were estimated using a 5Mb block-jackknife.

Because f(A|B) estimation requires high-coverage genomes for the "B" population, we used the Neandertals *D17, D5 (18)*, *Vi33.19* (8) *Chag8* (25) and the Denisovan *D3* (10), which were genotyped and filtered as described (SI Appendix 3). We also estimated the split time of *D17* to a present-day human, the Mbuti individual HGDP00982 (10). To mitigate the effect of ancient damage and recurrent mutations, we restricted the analyses to transversions and biallelic sites.

To polarize the ancestral/derived state, we required an outgroup. For the majority of the analyses, we used a combination of four apes: chimpanzee (panTro4, GCA_000001515.4), bonobo (panPan1.1, AJFE00000000.2), gorilla (gorGor3, GCA_000001515.4) and orangutan (ponAbe2, GCA_000001545.3) (Table S19) and required that all four must have the same allele, which was assumed to be ancestral. We did not tolerate any missing data in the outgroups. As a check, we also explored an alternative using only chimpanzee and gorilla as outgroups (Table S20).

Finally, to render the F(A|B) values into population split times, we used the demographic history of population "B" as estimated using PSMC (42) (SI Appendix 11). Specifically, we followed the same calibration pipeline with the correction factors reported in Table S15 and the parameters in Table S16 to obtain the calibration curves. We assumed a mutation rate of $1.45 x 10^{-8}$ per base pair per generation, and a generation time of 29 years (~$0.5\ 10^{-9}$ per base per year). We sampled genomes from the simulations listed in Table S16 at time points from 0 to 200,000 years with steps of 1,000 years, and from 200,000 years to 800,000 years with steps of 5,000 years following Sümer *et al.*, 2024 (43). We ran ten replicates for each time point and calculated the F(A|B) statistics for each simulation. The calibration curves allowed us to estimate the split times that best fit the F(A|B) values.

We first estimated the f(A|B) values by using all four apes as outgroups. The results are summarized in Table S19 and Fig. S32. Comparison with Table S20 shows that the effect of outgroup choice (compared to using only two apes) is negligible. Thus, we used the more conservative approach with four apes for all subsequent analyses.

Because we anticipate that f(A|B) estimation can be sensitive to different coverage-based GC-filtering (SI Appendix 3), we repeated the results with a minimum of 0.05 on the lower end of the GC-coverage. The effect of GC-filtering can be seen by comparing Table S19 and S21.

Consistent with other analyses, we find that *D17*'s closes split is with *D5*, and that this split occurred at ~10 ky before the death of D5. Thus, we decided to pay special attention to those two Neandertals and

compare their split times with other archaic genomes. Because each high-coverage genome has different regions that pass the quality filters, we re-estimated the f(A|B) values using only sites that are both present in *D17* and *D5*. We present these results in Table S22. The values of f(A|B) and inferred split time either with D5 or D17 as population A, to other genomes as population B are virtually indistinguishable, highlighting the fact that they are more related to each other than to the other high coverage Neandertals.

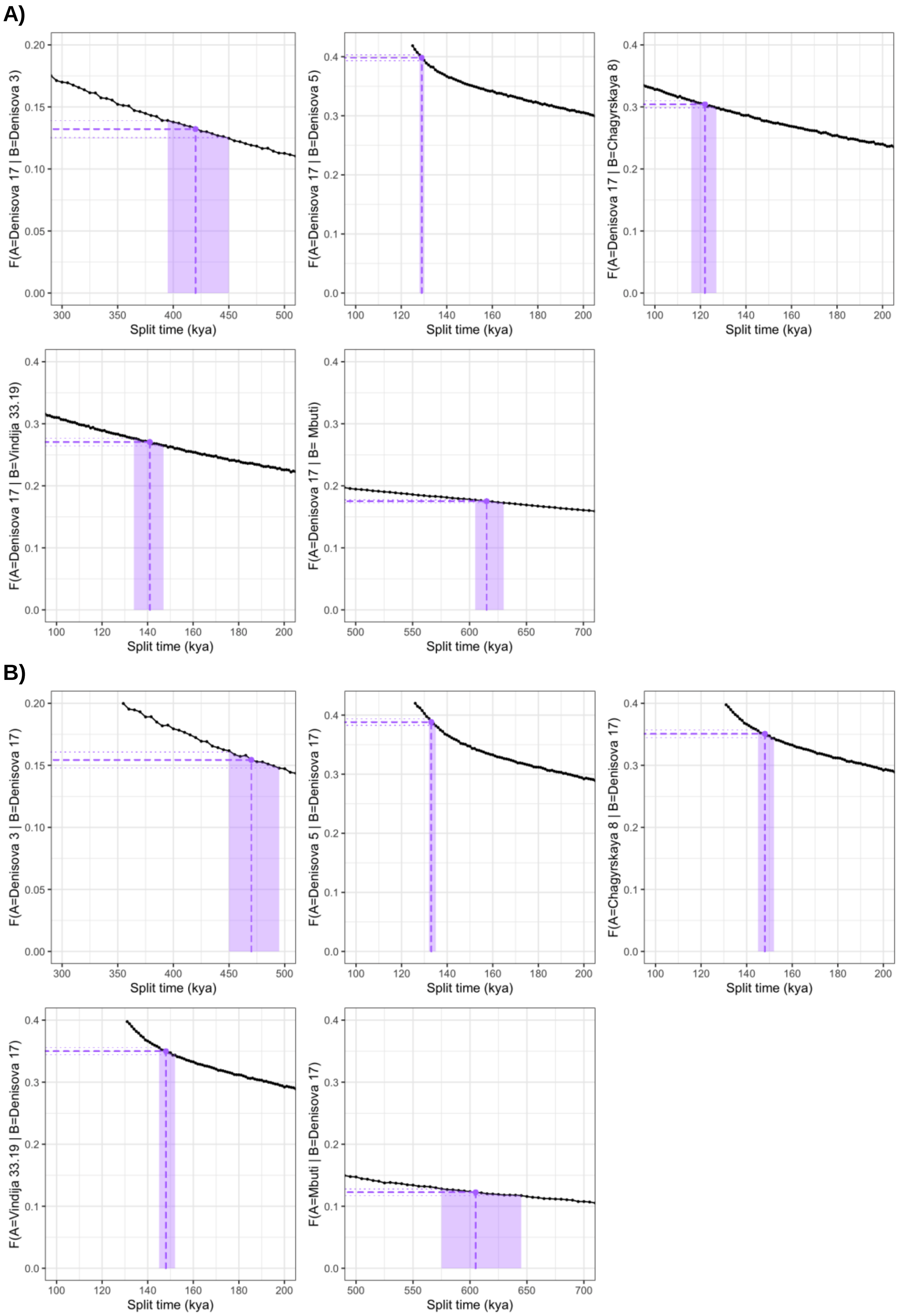

Figure S32: Calibration curves for translating f(A|B) to split time. The curves of population B are depicted in black, and the intersecting f(A|B) values with their 95% CI interval appear in purple **A)** the Neandertal *D17* as population "A". **B)** The Neandertal *D17* as population "B".

Table S19: Estimated f(A|B) values and population split times of *D17* and other high-coverage genomes, using chimpanzee, bonobo, gorilla and orangutan as outgroups. Split times assume a mutation rate of ~0.5 10^{-9} per base per year. For the branch shortening, we used the following ages: 50 kya years for *Vi33.19*; 120 kya for *D17* and *D5*; and 60 kya for both *Chag8* and the Denisovan *Denisova3*.

Population A	Population B	f(A|B) (%)	σ (%)	Split time (ky)	Split time + branch shortening (kya)
Denisovan D3	*Neandertal D17*	15.43	0.32	350 [375 - 330]	470 [495 - 450]
Neandertal D5		38.83	0.27	13 [15 - 12]	133 [135 - 132]
Neandertal Chag8		35.08	0.30	28 [32 - 25]	148 [152 - 145]
Neandertal Vi33.19		34.99	0.29	28 [32 - 25]	148 [152 - 145]
Mbuti		12.25	0.26	485 [525 - 455]	605 [645 - 575]
Neandertal D17	*Denisova3*	13.21	0.35	360 [390 - 335]	420 [450 - 395]
	Neandertal D5	39.82	0.25	9 [10 - 8]	129 [130 - 128]
	Neandertal Chag8	30.41	0.28	62 [67 - 56]	122 [129 - 123]
	Neandertal Vi33.19	27.05	0.30	91 [97 - 81]	141 [147 -131]
	Mbuti	17.51	0.12	615 [630 - 605]	615 [630 - 605]

Table S20: Estimated f(A|B) values and population split times of *D17* and other high-coverage genomes, using chimpanzee and bonobo as outgroups. Split times assume a mutation rate of ~0.5 10^{-9} per base per year. For the branch shortening, we used the following ages: 50 kya years for *Vi33.19*; 120 kya for both *D17* and *D5*; and 60 kya for *Chag8* and *Denisova3*.

Population A	Population B	f(A|B) (%)	σ (%)	Split time (ky)	Split time + branch shortening (kya)
Denisovan D3	*Neandertal D17*	15.71	0.31	340 [365 - 325]	460 [485 - 445]
Neandertal D5		38.90	0.27	13 [15 - 12]	133 [135 - 132]
Neandertal Chag8		35.19	0.30	26 [24 - 30]	146 [144 - 150]
Neandertal Vi33.19		35.16	0.28	27 [24 - 30]	147 [144 - 150]
Mbuti		12.69	0.26	460 [490 - 435]	580 [610 - 555]
Neandertal D17	*Denisovan D3*	13.62	0.36	345 [375 - 320]	395 [425 - 370]
	Neandertal D5	39.92	0.24	9 [10 - 8]	129 [130 - 128]
	Neandertal Chag8	30.66	0.28	62 [67 - 56]	122 [127 - 116]
	Neandertal Vi33.19	27.28	0.30	87 [95 - 81]	137 [145 - 131]
	Mbuti	17.63	0.12	610 [620 - 595]	610 [620 - 595]

Table S21: Estimated f(A|B) values and population split times of *D17* (with the modified coverage-based GC filtering) and other high-coverage genomes, using chimpanzee, bonobo, gorilla and orangutan as outgroups. Split times had the underlying assumption of a mutation rate of ~0.5 10^{-9} per base per year. For the branch shortening, we used the following ages: 50 kya years for *Vi33.19*; 120 kya for *D5*, 110 kya for *D17,* and 60 kya for *Chag8* and *Denisova3*.

Population A	Population B	f(A|B) (%)	σ (%)	Split time (ky)	Split time + branch shortening (kya)
Denisovan D3	*Neandertal D17*	15.42	0.31	380 [405 - 355]	490 [515 - 465]
Neandertal D5		38.78	0.28	15 [16 - 14]	125 [126 - 124]
Neandertal Chag8		35.02	0.30	30 [33 - 27]	140 [143 - 137]
Neandertal Vi33.19		34.98	0.28	30 [33 - 26]	140 [143 - 136]
Mbuti		12.18	0.26	520 [565 - 490]	630 [675 - 600]
Neandertal D17	*Denisovan D3*	13.36	0.35	360 [380 - 335]	410 [430 - 385]
	Neandertal D5	40.07	0.26	9 [10 - 7]	129 [130 - 127]
	Neandertal Chag8	30.70	0.28	58 [64 - 54]	118 [124 - 94]
	Neandertal Vi33.19	27.30	0.30	87 [95 - 81]	137 [145 - 131]
	Mbuti	17.62	0.12	610 [620 - 595]	610 [620 - 595]

Table S22: Estimated f(A|B) values and population split times of *D17* (standard filtering) and *D5* (using only sites present in both individuals) to other high-coverage genomes, using chimpanzee, bonobo, gorilla and orangutan as outgroups. A mutation rate of ~0.5 10^{-9} per base per year and for the following ages were assumed: 50 kya years for *Vindija 33.19*; 120 kya for both *D17* and *D5*; and 60 kya for *Chagyrskaya 8* and *Denisova 3.*

Population A	Population B	f(A|B) (%)	σ (%)	Split time (kya)	Split time + branch shortening (kya)
Neandertal D17	*Denisovan D3*	13.28	0.35	360 [385 - 335]	420 [445 - 395]
	Neandertal Chag8	30.54	0.28	60 [66 -55]	120 [126 - 115]
	Neandertal Vi33.19	27.17	0.31	80 [85 - 74]	130 [135 - 124]
	Mbuti	17.59	0.12	610 [600 - 625]	610 [600 - 625]
Neandertal D5	*Denisovan D3*	13.25	0.34	360 [385 - 335]	420 [445 - 395]
	Neandertal Chag8	30.56	0.29	60 [66 - 55]	120 [126 - 115]
	Neandertal Vi33.19	27.18	0.29	80 [85 - 74]	130 [135 - 124]
	Mbuti	17.59	0.12	610 [600 - 625]	610 [600 - 625]

SI Appendix 13 – Demographic history and divergence between the Neandertal *D17* and other high coverage archaic genomes inferred from *momi2*

Fabrizio Mafessoni

To gain additional line of evidence into how *D17* is related with other archaic hominins, we modeled the demographic history of the four high-coverage Neandertals and the Denisovan *D3*, together with present-day Yoruba individuals from the Simons Diversity Project, using *momi2 (49).* The program estimates demographic parameters by finding the combination of split times, population sizes, and sample ages that best explains the observed site frequency spectra across populations/individuals under a specified model of population history. To polarize the site-frequency spectra and allow the estimation of the ages of samples, we used a chimpanzee (*pantro4*). We parsed the vcfs of the genomes with the script *momi.extract_sfs* from the *momi2* suite (49), and computed the site-frequency-spectra in genomic regions retained for all individuals using two different filters, one in which all archaic genomes are filtered using the same stratified GC-content criteria (standard filter), and a looser filter that accounts for the different GC content of *D17* (modified filter) (SI Appendix 3).

We optimized this model using the function *stochastic_optimize* and ran 300 bootstraps for 500 iterations to obtain 5% confidence intervals. Tip dates were estimated for the ancient samples and fixed to 0 for present-day populations. A list of constraints, built upon estimates from previous works, was used for the model and is represented in Table S23.

We estimate that the split between *D17* and *D5* occurred approximately 7,000 years after their common ancestor diverged from the lineage leading to the *Vi33.19* Neandertal (Fig. S33, top panel), with 97% and >99% of the bootstraps supporting this scenario for the standard filter, and for the modified one, respectively. The range of estimates for the length of the shared branch between *D5* and *D17* vary slightly between the two filters (5-15ky for the standard filter and 5-20ky for the modified one), though the mode of the bootstrapped estimates peak at ~7ky for both filters. For both filters, *D5* is estimated to be older than *D17* (100% and >99% of the bootstrap samples when using the standard or the modified filter, respectively) by approximately 5-15 ky (Fig.S33, middle panel). We then tested if *D5* is directly ancestral to *D17*, by how long the private branch of *D5* is. We find that the population of *D5* was separated from that of *D17* for up to approximately 10ky. This indicates that *D5*, while not a direct ancestor of *D17*, lived in a population that shared most of its history with that of the latter (Fig. S33, bottom panel). It is worth noting that the effective population sizes of these groups are very small, suggesting that part of the observe separation may reflect local population structure rather than complete isolation. Finally, we estimate that *D17* lived 98.5 kya (94.5-109.8kya) and 99.4 kya (95.7-105.6kya), using the standard and modified filter, respectively (Fig. S34, S35).

Overall, we observe a general agreement with previous studies and consistency in the inferred demographic scenarios using the two different filtering schemes (Fig. S34, S35) with only minor differences.

For instance, we estimate that the split between Neandertals and modern humans occurred approximately 601 kya (550-630) or 560 kya (513-584-kya) using the standard and modified filter, respectively. Similarly, we infer an introgression from modern humans into Neandertals of 11.8% (2.7-16.4%) occurring around 291 kya (195-327kya) with the standard filter, compared to 8.9% (2.8-14.2%) and 264 kya (191-305kya) with the modified filter.

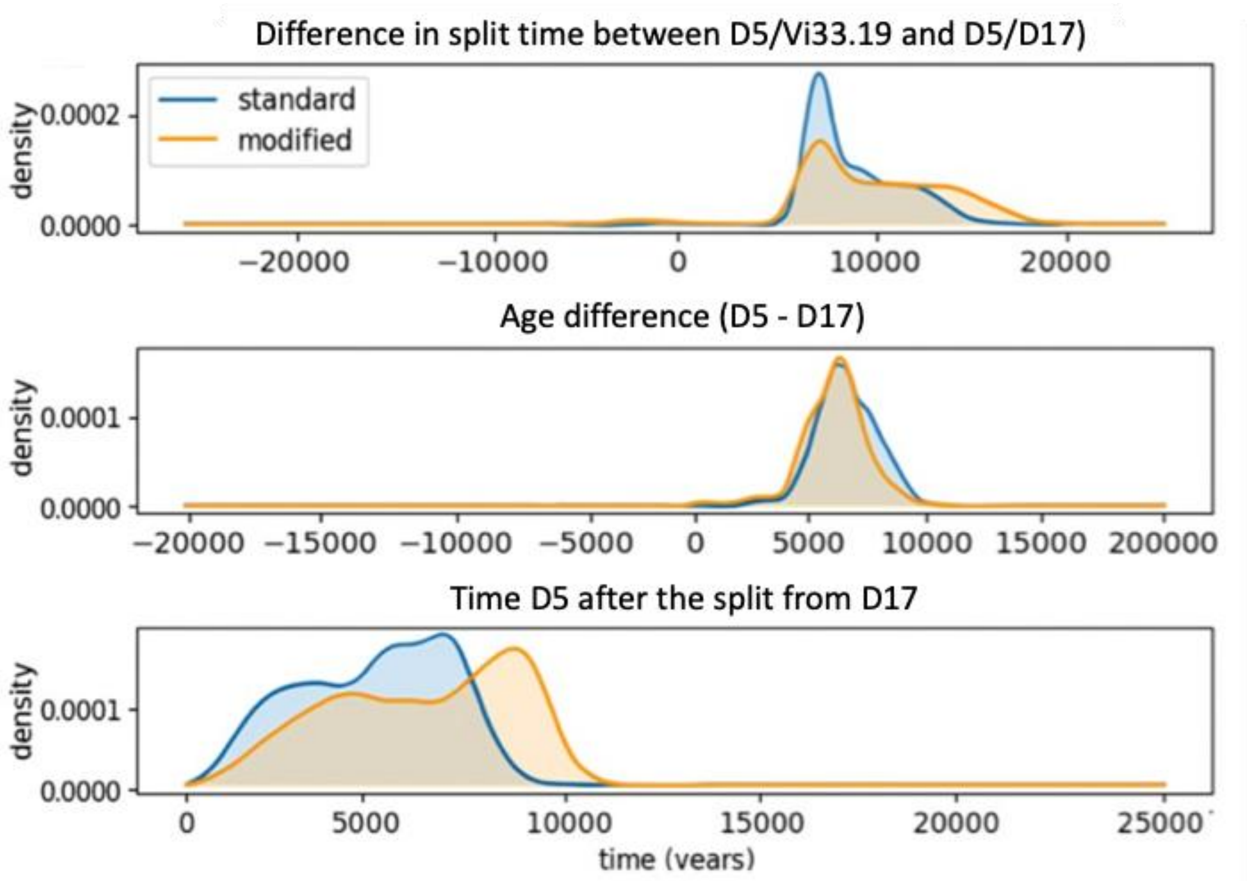

Figure S33. Distribution of the *momi2* bootstrap estimates for different parameters and different filtering schemes. Blue indicates estimates obtained using a site-frequency-spectrum estimated from genomes filtered using the same GC-content criterion (standard), while orange indicates the usage of a more relaxed GC-content filter for *D17*. Top) Distribution of the difference between the split time of *D5* from the *Vi33.19/Chag8* populations, and that of *D5* from the ancestral population of *D17*. Positive values indicate that the split of *D5* from *Vi33.19* preceded that of *D5* and *D17,* or in other words how long it took to the lineages of Neadertals found in Denisova case to separate after their split from the other high-coverage Neandertals (*Chagyrskaya 8* and *Vindiia 33.19*). Middle) Age difference between *D5* and *D17*. Bottom) Length in time of the private branch of *D5*, after the split from *D17*.

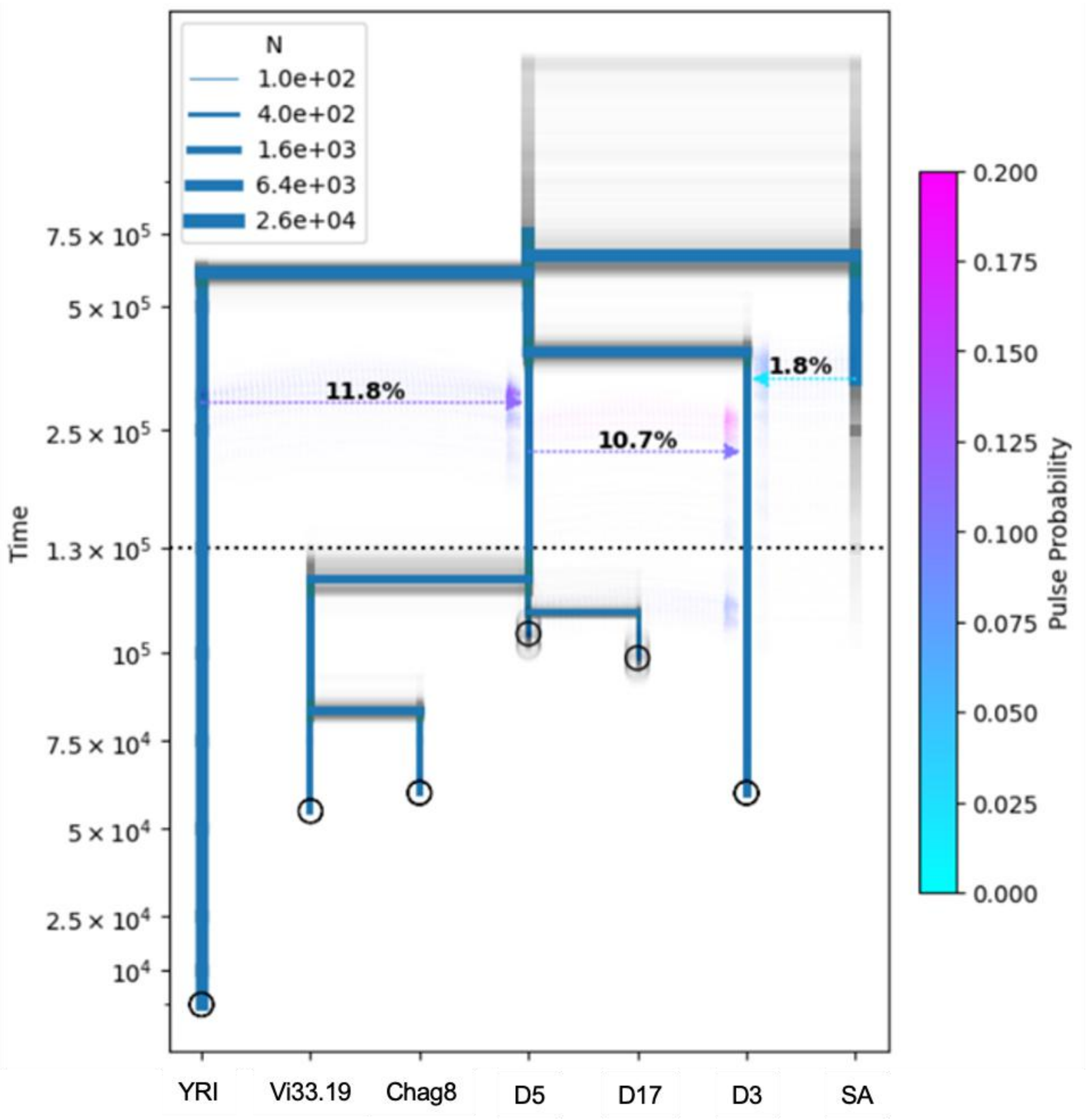

Figure S34. Model estimates from *momi2* applying the standard genomic filter for Yoruba (YRI), Neandertals *Vi33.19, Chag8, D5* or *D17 (Den17)*, Denisovan *D3* and a "super-archaic" (SA) hominin introgressing into Denisovans. Grey transparent shadows indicate estimates from individual bootstrap iterations (resamplings of the site-frequency-spectrum). The thickness of blue lines indicates the estimated effective population sizes as described in the legend. The color of the arrows indicates the proportion of admixture as indicated in the legend. Y-axis indicates time in years.

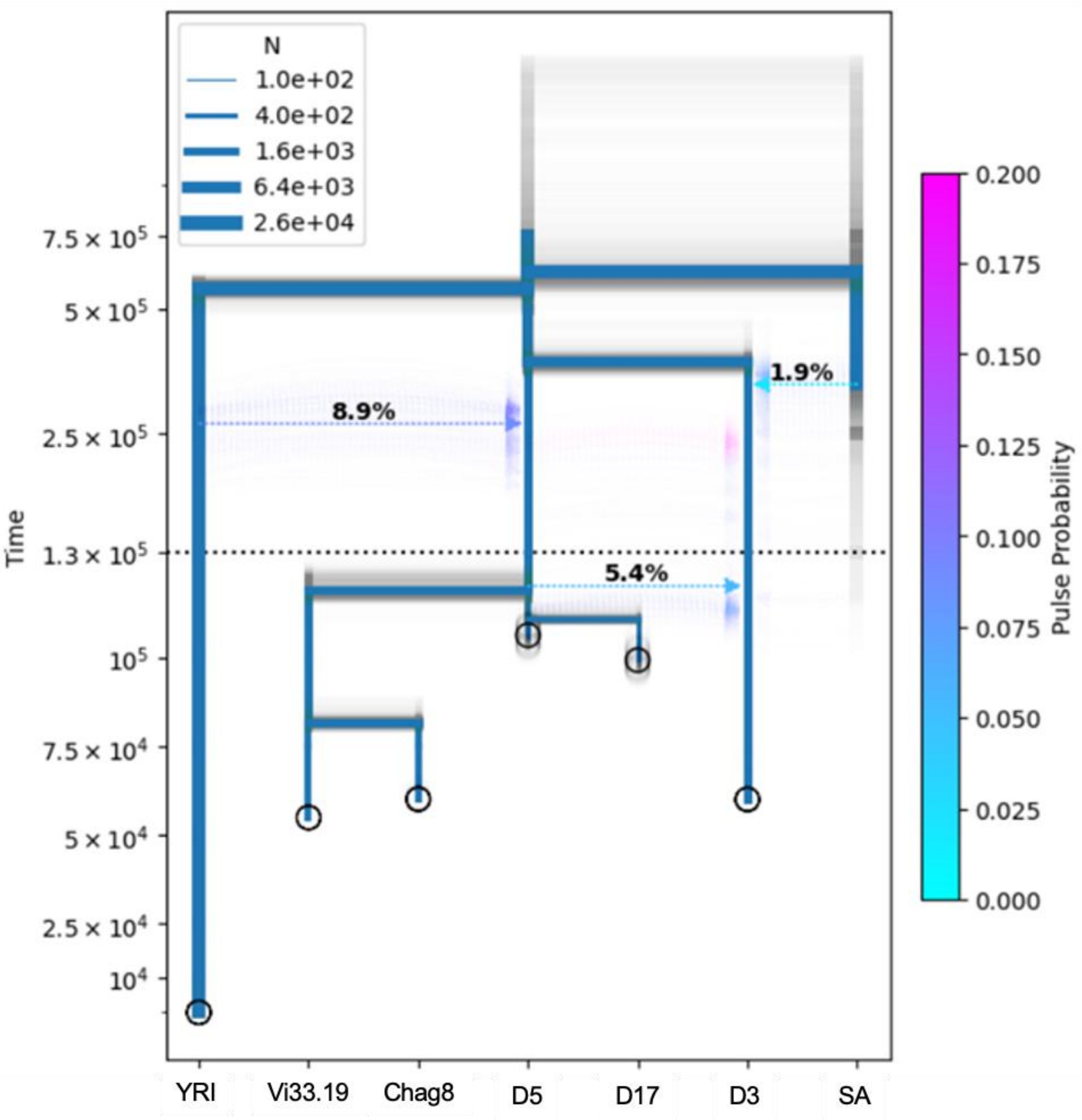

Figure S35. Model estimates from *momi2* applying the modified genomic filter for Yorubas (YRI), Neandertals *Vi33.19, Chag8, D5*, *D17*, Denisovan *D3* and a "super-archaic" (SA) hominin introgressing into Denisovans. Grey transparent shadows indicate estimates from individual bootstrap iterations (resamplings of the site-frequency-spectrum). The thickness of blue lines indicates the estimated effective population sizes as described in the legend. The color of the arrows indicates the proportion of admixture as indicated in the legend. Y-axis indicates time in years.

1045 **Table S23. Range of constraints and description of the parameters used in the model.**

Parameter	Meaning	Minimum value	Maximum value
n_yri	Ne Yoruba	500	100,000
n_D5	Ne *D5*	50	20,000
n_HND	Ne Ancestral archaic-modern humans	500	50,000
t_HND	Split archaic-modern humans	500,000	750.000
t_D5	Age *D5*	100,000	145,000
n_D3	Ne *D3*	50	20,000
n_ND	Ancestral archaic hominin Ne	500	50,000
t_ND	Split time Neandertal-Denisovans	300,000	t_den5_yri
t_D3	Age *D3*	60,000	80,000
n_Vin	Ne Vindija 33.19	50	20,000
n_N	Ne *D5*-Vindija 33.19 ancestor	500	20,000
t_Vin	Age Vindija 33.19	45,000	55,000
t_N	Split Time *D5*-Vindija 33.19	t_den5, t_vin	200,000
n_D17	Ne *D17*	50	20,000
n_D17_D5	Ancestral Ne *D7-D5*	500	20,000
t_D17	Age *D17*	60,000	145,000
t_D17_D5	Split time *D17-D5*	t_den5, t_den17	200,000
n_Chag8	Ne Chagyrskaya 8	50	20,000
n_Chag8_Vin	Ne Vindija-like Neandertals	500	50,000
t_Chag8_Vin	Split time Vindija 33.19-Chagyrskaya 8	80,000	200,000
t_Chag8	Age Chagyrskaya 8	60,000	90,000
p_pND	Prop. Introgression Neandertal Into Denisovans	0,0001	0.2
tm_ND	Time of Introgression Neandertal into Denisovans	t_den3,t_den5	t_den5_vin
p_HN	Prop. Introgression modern humans into Neandertals	0,0001	0.15
tm_HN	Time Introgression modern humans into Neandertals	t_den3_den5	t_den5_vin
p_SD	Prop. “super-archaic” introgression	0	0.1
t_SA	Time Split “super-archaic”	0	2,000,000
tm_SD	Time “super-archaic” introgression	t_den3,t_den5	t_SA,t_den3_den5

Table S24. Parameter estimates when the site frequency spectrum is computed filtering genomic regions according to the standard filter. Confidence intervals were estimated as the 2.5% and 97% quantiles of the estimates from bootstrapped data. Parameters are described in Table S23 and are given using the following naming convention: *t* indicates dates from branch shortening estimates, split times between two populations or introgression events; *n* indicate effective population sizes; *p* indicates the proportion for introgression events.

Parameter	Mean	2.50%	97.50%
n_yri	11853.11	11193.85	12205.27
n_D5	512.5889	108.3694	802.5221
n_HND	13459.61	13075.82	13882.95
t_HND	601352.6	549655.9	630235.4
t_D5	105373.2	101182.9	112089.4
n_D3	1879.546	1759.621	1982.602
n_ND	5715.127	3726.948	7602.796
t_ND	386886.2	355895.7	441685.6
t_D3	60085.26	60010.75	60313.95
n_Vin33.19	1058.787	954.5023	1266.748
n_N	2057.467	1903.348	53694.36
t_Vin33.19	54969.57	54742.69	54996.36
t_N	121066.1	116860	129172.4
n_D17	403.0179	212.9747	510.8323
n_D17_D5	716.6373	500	1945.703
t_D17	98457.1	94489.72	109824.7
t_D17_D5	111292.9	110296.1	118929.7
n_Chag8	4207.82	231.757	9536.149
n_Chag8_Vin33.19	1975.973	1470.224	2396.957
t_Chag8_Vin33.19	83508.56	81186.35	88336.24
t_Chag8	60089.79	60008.72	61003.86
p_pND	0.10726	0.016261	0.2
tm_ND	221649.2	106302.8	274819.6
p_HN	0.117705	0.02731	0.164443
tm_HN	291572.4	195303.4	326942.3
p_SD	0.017679	0.000997	0.15
t_SA	664974.8	599361.3	1960267
tm_SD	333039.8	116504.8	400713.1

Table S25. Parameter estimates when the site frequency spectrum is computed filtering genomic regions according to the standard filter. Confidence intervals were estimated as the 2.5% and 97% quantiles of the estimates from bootstrapped data. Parameters are described in Table S23 and are given using the following naming convention: *t* indicates dates from branch shortening estimates, split times between two populations or introgression events; *n* indicate effective population sizes; *p* indicates the proportion for introgression events.

**Parameter**	**Mean**	**2.50%**	**97.50%**
n_yri	11923.15	11191.66	12214.6
n_D5	438.9854	105.6878	738.3242
n_HND	13401.07	13051.81	13743.26
t_HND	560036.2	513051.2	584187.6
t_D5	106419.2	102636.4	109773.3
n_D3	1769.105	1650.314	1870.379
n_ND	4408.16	3064.052	6005.633
t_ND	372436	352746.9	428626.7
t_D3	60080.75	60008.5	60286.31
n_Vin	997.9802	898.1562	1177.544
n_N	1932.641	1804.615	2088.581
t_Vin	54970.84	54859.96	54994.16
t_N	119503.5	116527.6	125261.9
n_D17	365.3493	179.7865	451.8079
n_D17_D5	568.4179	500	1215.848
t_D17	99392.88	95737.87	105576.1
t_D17_D5	111035.2	110138.5	114879.6
n_Chag8	4095.626	288.2975	9580.059
n_Chag8_Vin	1870.43	1564.307	2183.963
t_Chag8_Vin	81986.36	80305.08	86381.63
t_Chag8	60138.49	60019.56	61499.63
p_pND	0.05367	0.021157	0.2
tm_ND	118786.4	108557.9	253942.6
p_HN	0.089002	0.028258	0.141522
tm_HM	263729.3	191072.1	304946.9
p_SD	0.018585	0.001535	0.125973
t_SA	616943.1	556755.5	1956563
tm_SD	329562.3	117349.9	386388.4

SI Appendix 14 – Population structure and effective population size estimates

Fabrizio Mafessoni

The fraction of the *D17* genome covered by HBD tracts is the highest among archaic genomes (SI Appendix 9). This is consistent with previous suggestions that Neandertals lived in small groups. We modeled the populations in which archaic hominins lived as metapopulations (many small, partially connected groups), characterized by groups of size *N*, a migration rate between groups *m* and a total number of groups *D*. The metapopulation was modeled as fully connected, with each group potentially symmetrically exchanging migrants with all other groups. We ran 10^4 full genome coalescent simulations using *scrm (44)* to approximate the likelihood to observe proportions of the genomes covered by HBD tracts given combinations of the parameters *N, m* and *D*. The likelihood was maximized using the approach used in Mafessoni *et al*., 2020 (25). Specifically, HBD tracts were classified as tracts longer than 10cM and as tracts between 2.5cM and 10cM, and the likelihood of observing a proportion of the genome falling in either of these two categories was approximated using simulations and maximized numerically using the R package *optimx (50)*. Confidence intervals were estimated using a normal approximation of the 95%-likelihood-ratio based confidence interval. Fig. S36 shows the combinations of parameters *N* and *m* that are compatible, *i.e.,* within the confidence interval, for *D* set to 20 groups. Siberian Neandertals (*D5*, *D17* and *Chag8*) appear strikingly different from ancient modern humans (AMH) (here represented by *Ust'Ishim*) and from the Denisovan *D3*. Interestingly, a likelihood ratio test (p-value<0.01) also supports a model in which they differ from the *Vi33.19* Neandertal, whose HBD tracts are compatible only with groups as small as 50 individuals when migration rates between local groups (demes) exceed >10%. This is in contrast to Siberian Neandertals, for which we estimate small group sizes (<50 individuals) and migration rates higher than 10% only under implausible scenarios, *i.e.*, group sizes <5 individuals. These values are similar when HBD tracts are determined using the optimal parameter π determined with the procedure described in SI Appendix 9 and genetic length of HBD tracts estimated using an African-American recombination map (Fig. S36), when less heterozygous sites/lower errors are allowed to break putative HBD tracts, *i.e*., the parameter π is set to a more stringent value of 0.99 (Fig. S37), or when a different recombination map is used (Fig. S38). Estimates for *D17* are not affected by the filtering scheme (Fig. S39).

The genome of *D17* allows us to explore the factor underlying the apparently smaller group sizes of Eastern Neandertals. Specifically, we can test whether ancestry (the *Vi33.19-Chag8* vs *D5-D17* subdivision) or geographical subdivision between the Western *Vindija Neandertal* and the Siberian Neandertals better explain the distributions of HBD tracts in Neandertals. To do this, we defined groups of individuals (Siberian Neandertals, Denisova Cave Neandertals, the "Late Neandertals" *Vi33.19* and *Chag8*, all Neandertals together) and estimated a single N, m and D parameter for each of these groups by maximizing the log likelihood. In other words, we assumed that within each group, all individuals lived in

groups of similar *N*, *m* and *D* parameters. We then used these to compute the likelihood of the different models describing different population subdivisions: Siberian Neandertals versus *Vi33.19,* Neandertals found in Denisova Cave versus "Late Neandertals" and all the four high coverage Neandertals together. We calculate the Akaike Information Criterion (AIC) of each model. The model with the lowest AIC, hence the most supported, is the subdivision between Siberian Neanderthals versus *Vi33.19* (AIC=-18.25). This model is significantly better than a model assuming that all Neandertals had the same population structure (relative likelihood = 0.008, AIC=-8.61) and a model subdividing Neandertals by ancestry (*Chag8*-*Vi33.19* versus *D5-D17*) (relative likelihood = 0.031, AIC=-11.32), potentially indicating that the ecological conditions that Neandertals experienced in Siberia had a stronger role than ancestry or population history. Note, however, that since *Vi33.19* is the youngest Neandertal and *Chag8* the second youngest, both subdivisions are conflated with time, leaving the temporal differences as a potential alternative explanation. The estimated number of individuals per group (*N*) and migration rates (*m*) for 20 demes for these population groups are shown in Figure S40.

We note that not only differences in group size but also in migration rates among groups may contribute to these results. Thus, under a metapopulation models consisting of 20 groups with fewer than 50 individual each, our results would indicate that modern humans and Denisovans had high migration rates (60-100%) between groups. In comparison, the metapopulation of late Neandertals from Vindija would have a more intermediate migration rate and the Siberian Neandertals markedly lower migration rates, approximately 1% for *D17*, and around 5% for *D5* and Chag8 suggesting more structured and isolated metapopulation of Neandertals in the Altai. Note that a model with full migration among groups (100%) lies outside of the confidence intervals for all Siberian Neandertals, but not *Vi33.19*, nor the Denisovan *D3,* when analysed individually (Figure S36, S37). This is consistent with the fact that a panmictic model with a single group (D=1, m=0) is rejected when contrasted with the metapopulation model for all Siberian Neandertals (likelihood ratio test p-value <0.01), excluding that a long-term low Ne can explain the observed patterns, while such model cannot be excluded for *Vi33.19* and Denisovan *D3*, when analysed individually (likelihood ratio test p-value 0.97 and 1, respectively).

We emphasize that our metapopulation model only considers long HBD tracts, which are mostly influenced by recent history (coalescent events occurred less than 100 generations before the observed genomes (25) but not long-term population dynamics and the whole Neandertal population. Thus, the total size of the estimated metapopulation (N*D) only aims to capture local groups, and not the total Neandertal population or long-term dynamics. Despite this, notice that the low N*D values (lower than 1000 individuals all for Siberian Neandertals) are comparable with estimates for the last time interval of the inferred demography by PSMC (399, 618 and only 10 individuals for *D17*, *D5* and *Chag8*, respectively). This likely reflects the fact that the decrease in estimated Ne by PSMC at the end of each demographic history is also caused by strong population substructure, consistently with the effects of structure on PSMC and supporting the notion that Neandertals lived in small groups (51, 52). We also examined the effects of population structure on the overall heterozygosity. First, we performed 100 whole genomes coalescent simulations for

the ranges of parameters N, D and m estimated for Neandertals and noticed that the overall expected heterozygosity is not significantly reduced compared to a single panmictic population of the same size. This occurs, despite the increased proportion of the genome in HBD, because in a metapopulation, haplotypes coming from different demes have deeper coalescences and thus more differences, “compensating” for the reduction in heterozygosity in HBD regions. Second, we computed heterozygosity when masking HBD tracts in simulated genomes. We estimate that this is 13-42% higher than the overall heterozygosity, consistently with the observations from the genomes (Table S11).

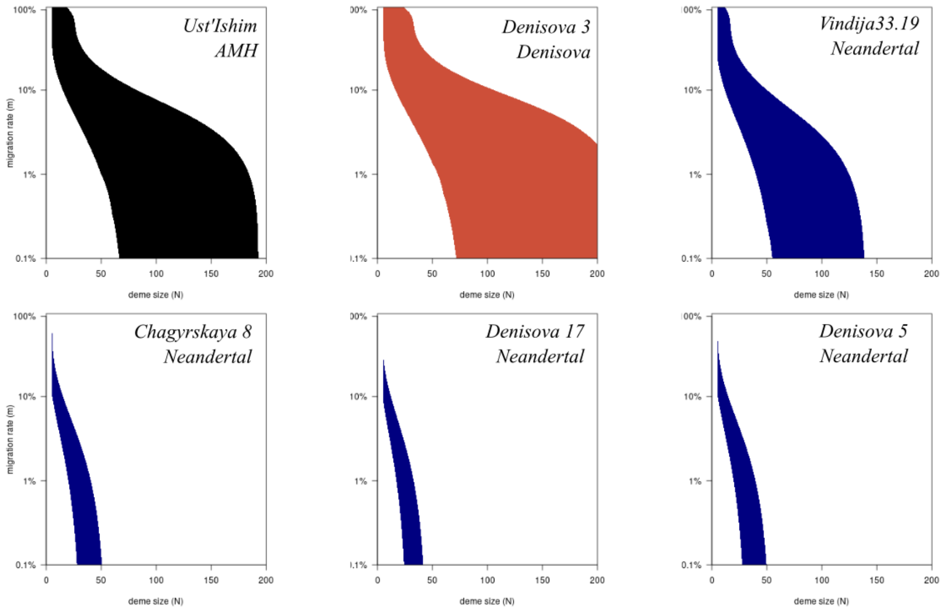

Figure S36. Likelihood surfaces for the metapopulation model for the modern human *Ust'Ishim* (black), the Denisovan *D3* (orange) and the four high coverage Neandertals (blue) (*Vindija33.19 (Vi33.19), Chagurskaya 8 (Chag8), Denisova 17 (D17) and Denisova 5 (D5)*), when the number of demes *D* is set to 20 and HBD tracts are estimated using the optimal π parameter and the African-American recombination map. The *N* parameter (group size) is shown on the x-axis, while *m* (migration rate) is shown on the y-axis in terms of percentage of migrants exchanged each generation between groups.

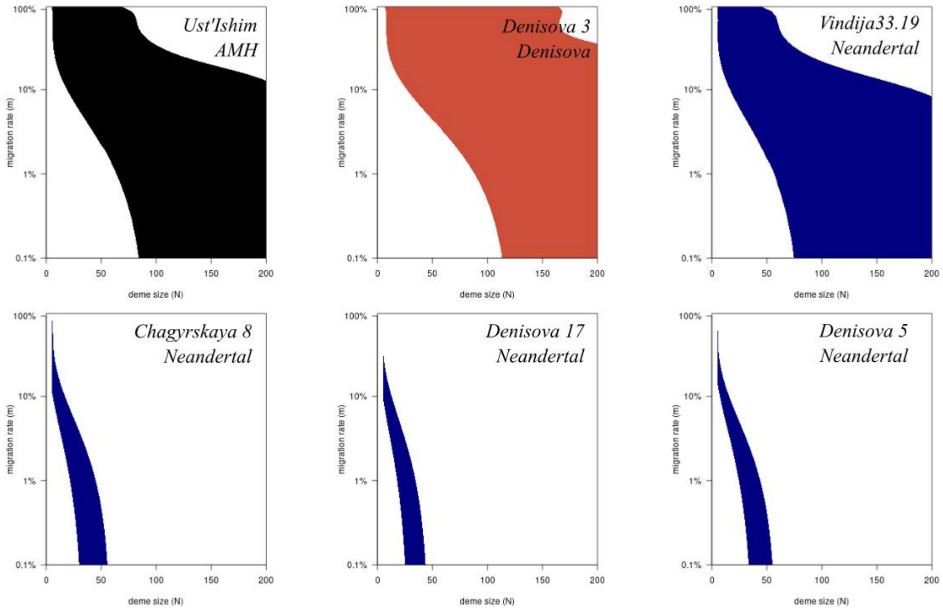

Figure S37. Likelihood surfaces for the metapopulation model for Ust'Ishim (black), D3 (red) and the four high coverage Neandertals (blue) (*Vindija33.19 (Vi33.19), Chagurskaya 8 (Chag8), Denisova 17 (D17) and Denisova 5 (D5)*),, when the number of demes D is set to 20 and HBD tracts are estimated using π=0.99 and the African-American recombination map. The N parameter (group size) is shown on the x-axis, while m (migration rate) is shown on the y-axis in terms of percentage of migrants exchanged each generation between groups.

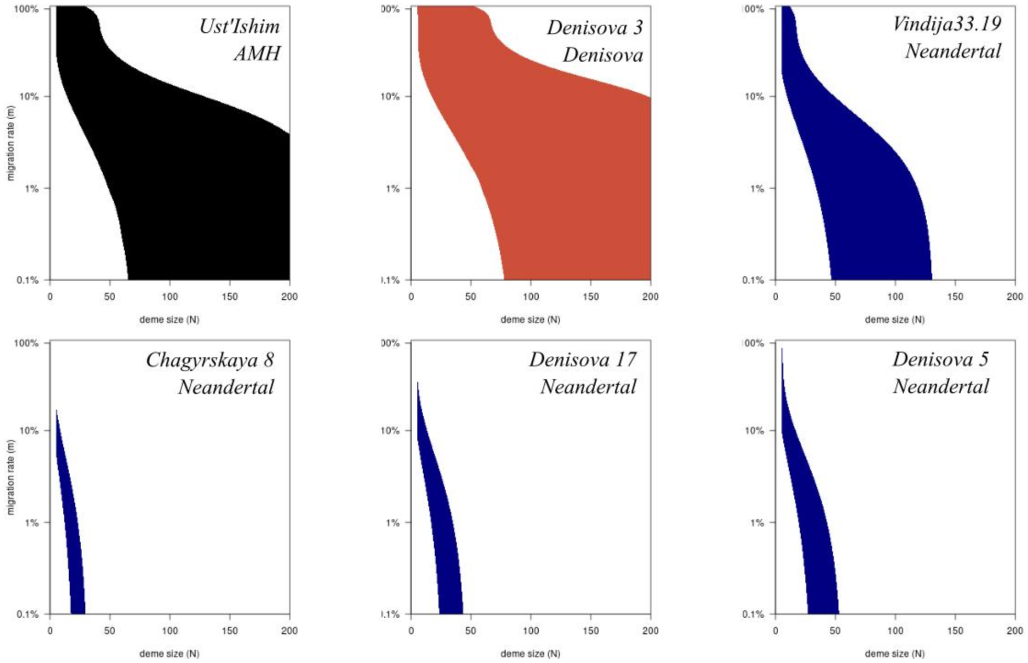

Figure S38. Likelihood surfaces for the metapopulation model for *Ust'Ishim* (black), *D3* (red) and the four high coverage Neandertals (blue) (*Vindija33.19 (Vi33.19), Chagurskaya 8 (Chag8), Denisova 17 (D17) and Denisova 5 (D5)*),, when the number of demes *D* is set to 20 and HBD tracts are estimated using the optimal π parameter and the European (EUR) recombination map. The *N* parameter (group size) is shown on the x-axis, while m (migration rate) is shown on the y-axis in terms of percentage of migrants exchanged each generation between groups.

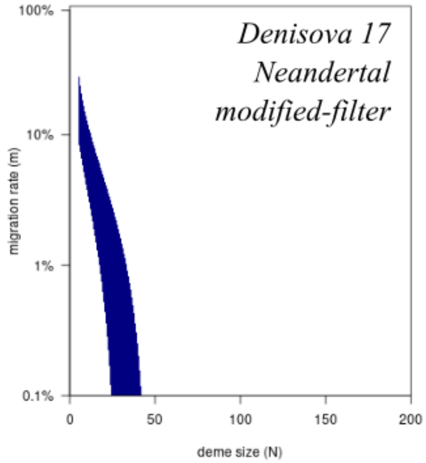

Figure S39. Likelihood surface for *D17 (Denisova 17)* with looser filtering scheme for number of demes *D* equal to 20 and HBD tracts estimated using the optimal π parameter and the African-American recombination map.

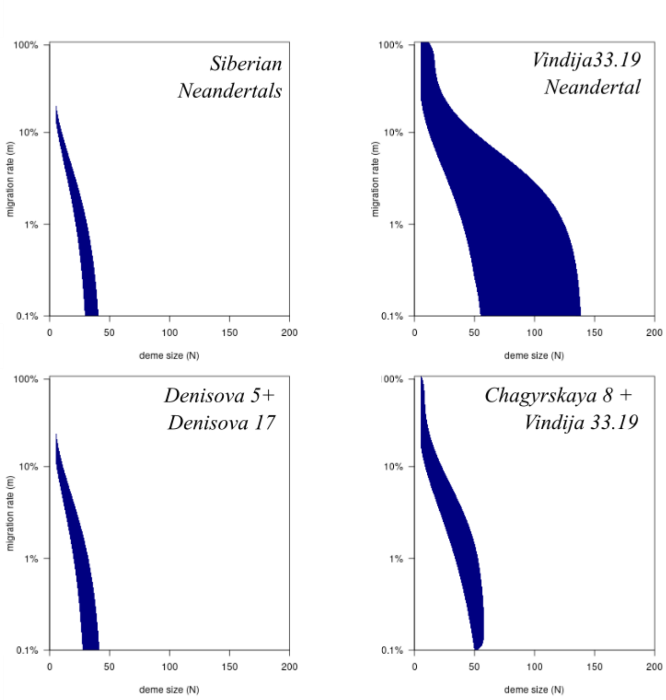

Figure S40. Parameter estimates (blue) for different groups of Neandertals for the groups included in the geographical subdivision of Neandertal genomes (top row, the earlier Siberian Neandertals (D5, D17 and Chag8) versus the later Croatian *Vindija 33.19* Neandertal (Vi33.19)) and the subdivision based on ancestry in *Chagyrskaya-Vindija 33.19* versus Neandertals from Denisova Cave (bottom row). Note that the better explanatory power of the top model is reflected in stronger differences in the parameter differences between the two groups.

SI Appendix 15 – Genetic differentiation among Neandertals

Hugo Zeberg

To quantify the differentiation among Neandertals across their geographical range, we estimated the fixation index (F_{ST}), which measures the proportion of genetic variation explained by population structure. However, the small sample size presents two challenges. First, Wright's original estimator of F_{ST} (53) is upwardly biased for small samples, a limitation that can be addressed by the Hudson estimator (54, 55). Second, small samples yield large standard errors in allele-frequency estimates, which can be mitigated by considering many variants.

We implemented the Hudson estimator of F_{ST} (54) in a custom Bash pipeline. For each population pair, allele frequencies were obtained using `PLINK 2.0` (`--freq`) from VCF files restricted to biallelic SNPs passing standard quality filters. In addition, all analyses were performed after excluding genomic regions tentatively identified as Denisovan introgressed fragments in the *D17* genome. Hudson's F_{ST} was then computed as a ratio of averages across all sites:

$$F_{ST} = \frac{\overline{(p_A - p_B)^2 - p_A(1 - p_A)/(n_A - 1) - p_B(1 - p_B)/(n_B - 1)}}{\overline{p_A(1 - p_B) + p_B(1 - p_A)}}$$

where:
p_A = allele frequency in population A
p_B = allele frequency in population B
n_A = number of chromosomes sampled in population A
n_B = number of chromosomes sampled in population B

To assess the reliability of the Hudson estimator under our small sample size condition, we first, estimated the *Fst* between 108 Yoruba and 104 Japanese genomes, the population pair with the highest F_{ST} among population represented in the 1,000 Genomes project (56, 57). Confidence intervals were estimated by block-jackknifing over non-overlapping 5 Mb genomic windows. We then compared these values with those from random subsamples of single genome or genome pairs per group. Our result show that single genomes from each population are consistent with the estimate from the full cohort (F_{ST} = 0.164, 95% CI: 0.162–0.166) (Fig. S41A). A similar result was obtained for 10 Mbuti versus nine Papuan Highlander genomes (F_{ST} = 0.267, 95% CI: 0.263–0.271), a pair long recognized as highly differentiated(57, 58). Again, single-genome estimates matched those from the full sets of genomes (Fig. S41A). Finally, comparing an ancient modern human genome (*Ust'Ishim*) which is approximately 45,000 years old with present-day South Asian genomes (F_{ST} = 0.052, 95% CI: 0.049–0.056) produced results in agreement with comparisons between *Ust'Ishim* and randomly sampled of South Asian individual genome (Fig. S41A). Together, these analyses show that the Hudson estimator performs well even when applied to single genomes or to genomes differing in age by up to 45,000 years.

Applying the same approach to Neandertals gives a different outcome in that subsampling single genomes results in inflated estimates relative to comparing two and three genomes against each other (Fig. S41A). This may be explained by the high level of autozygosity in Neandertals, which causes many segments of the two chromosomes in an individual to share resent ancestry and therefore not represent independent observations for estimating allele frequencies. To account for this, we estimated the degree of autozygosity by estimating individual-level inbreeding coefficients (*F*) from genome-wide heterozygosity (using `PLINK` `--ibc`), and used these to rescale the effective sample size:

$$n_{\mathrm{eff}} = \frac{n}{1+F}$$

This correction was applied only to the small-sample denominator term in the Hudson estimator. Autozygosity was estimated for each genome individually and incorporated into pairwise comparisons. This adjustment explicitly depends on sample size and is only relevant when sample sizes are small. After correction, F_{ST} values calculated between single genomes faithfully reproduce those calculated with all available high-coverage Neandertal genomes (Fig. S41B).

Pairwise F_{ST} values between the available Neandertal genomes reveal three clusters (Fig. S41C). First, F_{ST} is close to zero (95% CI: 0.00–0.014) between *Vi33.19* and the Neandertal *Goyet GN1*, an approximately 45,000-year-old Neandertal genome sequenced to ~ 22-fold coverage from Belgium that recently became available online (ENA accession PRJEB98484). Similarly, F_{ST} is close to zero between *D5* and *D17* (95% CI: 0.00–0.066). Henceforth, we refer to the pair *Vi33.19/Goyet GN1* as *Western* and the pair *D5/D17* as *Eastern* Neandertals (Fig. S41C). Notably, when corrected for shared descent between chromosomal copies, one chromosome of *D17* is ”as related” to a chromosome of *D5* as it is to the other *D17* chromosome; the same holds for *Vi33.19* and *GN1*. In addition, *Chag8* forms a distinct cluster, which we term *Western-derived* Neandertals, as it shares ancestry with *Western* Neandertals yet is differentiated from those (F_{ST} = 0.178, 95% CI: 0.152–0.204).

The differentiation between *Western* and *Eastern* Neandertals (F_{ST} = 0.299, 95% CI: 0.290–0.309; Fig. S41) is substantial, consistent with prolonged isolation of small populations, and slightly exceeds that observed among one of the most diverged present-day population pairs (Mbuti and Papuan Highlands, F_{ST} = 0.267, 95% CI: 0.263–0.271). Thus, Neandertals did not represent a panmictic Eurasian population. Instead, they were divided into distinct and small regional groups, far smaller and likely more isolated than those typical of modern human globally, despite being separated by relatively modest geographic distances.

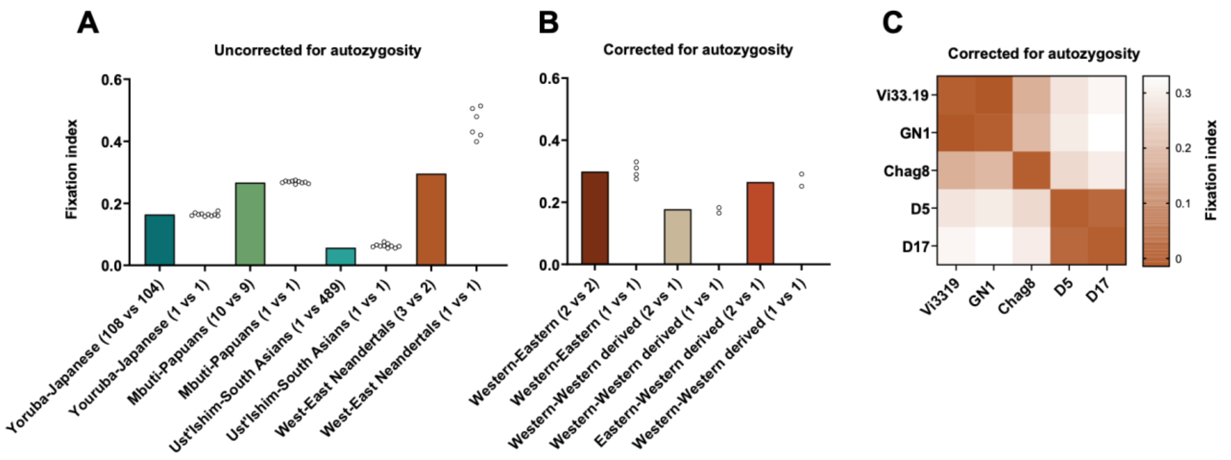

Figure S41. Fixation indices for modern and Neandertal population pairs. **A**) Fixation indices (Hudson's F_{ST}) for different population pairs. Bars show estimates based on multiple genomes per population, while open circles indicate values based on single-genome comparisons. Note the apparent inflation of F_{ST} between West (*Chag8* “C8”, *Goyet Q56-1* “GN1”, *Vindija 33.19* “V33.19”) and East (*D5*, *D17*) Neandertals when using single genomes. **B**) Heatmap of pairwise F_{ST} among high-coverage Neandertal genomes, corrected for autozygosity. Three clusters are evident: Western (V33.19, G56), Western-derived (*C8*), and Eastern (*D5, D17*). **C**) F_{ST} between the three Neandertal groups. Bars represent estimates based on several genomes, while open circles show subsampled single-genome comparisons.

SI Appendix 16 – Denisovan ancestry in Neandertals *D17* and *D5*

Leonardo N. M. Iasi

The discovery of an offspring of a Neandertal mother and a Denisovan father in Denisova Cave suggest that the Altai region was a geographic area where Neandertals and Denisovans met and admixed (59). Here we screened the high-coverage Neandertal genomes *D17* and *D5* for Denisovan ancestry to determine whether they contain evidence of recent admixture. We also investigate the timing of these potential admixture events and whether they trace back to a single occurrence.

Putative Denisovan ancestry in the Neandertal genomes

We inferred Denisovan ancestry in the genome of the *D5* and *D17* Neandertal genomes using *admixfrog* version 0.7.1(60). We defined set of informative sites, by identifying all positions in the genome where a randomly sampled allele from an individual with sub-Saharan-related ancestry (SGDP B_Mbuti-4 (10)) differed from the corresponding alleles in a panel of archaic genomes. This panel included five high coverage Neandertals, *D17*, *D5* (18), *Vi33.19* (8), *Chag8* (25), *GN1* (ENA accession PRJEB98484); and two high coverage Denisovans, *D3* (10) and *D25* (61). For all individuals, we excluded tandem repeats and indels and retained only regions that could be uniquely mapped in the genome using 35-mers and applied a GC-content-conditioned on coverage of ≥ 10x (SI Appendix 3). All archaic genotypes were called using snpAD version 0.3.11(15)

Following the approach of Meyer *et al.*, 2016 (19) we identified “diagnostic positions” by randomly sampling one allele from each individual and retaining only sites with non-missing genotypes in all genomes. Ancestral alleles were determined using the chimpanzee (*PanTro6*), gorilla (*GorGor3*), bonobo (*PanPan1*) and orangutan (*PanAbe3*) reference genomes aligned to the human reference genome (*hg19*; downloaded from http://hgdownload.cse.ucsc.edu/goldenpath/hg19/). Sites were kept when at least three of these species shared the same allele and at most one was missing. Only positions where at least one hominin carried a derived allele were retained. The resulting sites were filtered again using the mappability track map35_99 filter (retaining positions in the genome where a 35bp sequence maps with >99% confidence to a single location), yielding a total of 2,289,202 diagnostic positions.

We built our *admixfrog* reference panels from genotype information as follows:

Denisovan (DEN): *D3* and *D25*
Neandertal (NEA): *Vi33.19*, *Chag8* and *GN1*
Modern Humans/ African (AFR): We use all female individuals from the 1k Genomes including Mende, Mandenka, Yoruba, Esan and Luhya individuals.
Chimpanzee (PAN): chimpanzee reference genome *panTro6*.

The modern humans were used as reference for contamination estimates. The chimpanzee reference genome was used to infer the ancestral state for each variant. If no ancestral state information was

available, we used the default option of uniform priors in *admixfrog.* We used either the African American (AA map)(62) or the deCODE(41) recombination map to assign genetic distances.

The command used for *admixfrog* was:

```
admixfrog-bam --ref ref.file --bamfile input.bam --force-bam --deam-cutoff 3 --out output.in.xz --length-bin-size 15

admixfrog --infile  output.in.xz  --ref ref.file -o out.name \
--states NEA DEN  --cont-id AFR --ll-tol 1e-2 --bin-size 5000 \
--est-F --est-tau --freq-F 3  --freq-contamination 3 --e0 1e-2  --est-error \
--ancestral PAN  --run-penalty 0.1 --max-iter  250 --n-post-replicates 200 \
--filter-pos 50 --filter-map 0.000  --init-guess NEA
```

Depending on the recombination map used, we estimated putative Denisovan ancestry at 6.7-7% in the *D17* genome and at 6.9-7.2% in the *D5* genome (Tables S26). As *admixfrog* cannot distinguish Denisovan introgressed DNA from incomplete lineage sorting (ILS), these proportions likely reflect contributions from both processes.

Calling of putative Denisovan segments in the Neandertal genomes

We used the posterior probabilities from *admixfrog* to visualize putative Denisovan segments in each genome using two different penalties of 0.25 and 0.4 that control how the model treats the boundaries of these segments. Penalty 0.25 makes it more likely for the algorithm to split segments into smaller pieces, and 0.4 more likely to extend segments and join adjacent ones.

```
admixfrog-rlt --out output.rle.xz –in input.bin.xz --run-penalty [0.25,0.4]
```

We restrict our analysis to segments equal or longer than length cutoffs of 0.05 cM, 0.1 cM and 0.2 cM. If not stated otherwise we present the results under the African American map for penalty 0.4 and segments equal or longer than 0.2 cM, as this has been found to most effectively reduce false negatives (60, 63). We excluded segments overlapping the Major Histocompatibility Complex (MHC) since long term balancing selection in this region might maintain long segments of ILS (64). The number, average and combined length of segments longer or equal than 0.2 cM is listed in Table S27. The location of the segments in the *D17* and *D5* genomes are shown in Fig. S42.

We assessed whether the putative Denisovan introgressed segments identified in the *D17* and *D5* Neandertal genomes (Fig. S42) are truly Denisovan rather than false positives, by looking how reads overlapping Denisovan-specific SNPs in these segments matched Denisovan alleles. Figure S43 shows the frequency of reads matching to the reference genomes for the five longest identified segments in *D17* and *D5*. These long segments match multiple alleles found only in the Denisovan genome, which suggests that they originate from recent introgression events.

Correlation of the Denisovan ancestry in *D5* and *D17*

We tested whether the locations of autosomal segments of Denisovan ancestry in the *D5* and *D17* Neandertal genomes are correlated, following the approach previously described in Massilani *et al*, 2020

(65). Specifically, we measured the overlap between 0.005 cM bins that fall within inferred Denisovan segments longer or equal to 0.2 cM in both genomes. To establish the level of overlap expected by chance, we generated an empirical distribution of random segment sharing, by randomly shuffling the positions of the segments across the genomes and re-computing the overlap for each simulated scenario. This shuffling was repeated 1,000 times to produce an empirical distribution of "random sharing" from which we estimated *p*-values using the ecdf function in R by comparing the observed overlap to the random expectation. For each recombination map and penalty, the *p*-value of the observed overlap are statistically significant under a threshold of 0.05 (Table S28), indicating that the Denisovan segments in *D5* and *D17* overlap more than expected by chance. These suggest that at least part of the Denisovan ancestry in the two genomes derives from shared admixture events.

A formal possibility is that the two Denisovan genomes, *D3* and *D25,* used as reference, carry Neandertal ancestry, and that this confounds our analysis as we use these genomes as references to call Denisovan ancestry in the Neandertals. However, in that case, both Denisovans would need to have Neandertal segments at the same location, which seem unlikely considering that they lived almost 150,000 years apart.

Dating of Denisovan introgression event

We estimated the time since Denisovan introgression (t_m) in *D5* and *D17* using a Bayesian approach based on the length distribution of the inferred autosomal Denisovan segments in both genomes. In this model, t_m represents the rate parameter of an exponential distribution, which we used as the likelihood function, with a Gamma prior defined by shape parameter α, rate β, number of segments (*n*). The posterior distribution is obtained by multiplying the likelihood function by the prior distribution and then normalizing the result. The segment length distribution is truncated by a minimum length cutoff, which we account for by subtracting the truncation *c* from each segment length x_i (in Morgans). The shape parameter of the Gamma prior is given by adding the number of segments to the prior value of α, and the rate parameter is given by adding the sum of all (x_i - *c) values* to β.

$$P(t_m|\, x_i > c) = Gamma(t_m|\alpha + n, \beta + \sum_{i=1}^{n} x_i - c)$$

We estimated the time for all combinations of genetic map, penalty and minimum cutoff using a shape prior α = 2.5 and rate prior β = 0.0025 (Fig. S44).

There is considerable variation in the estimated dates when using different length cutoffs, but they remain largely stable between genetic maps and penalties. This suggests that the variation is not due to a single underlying distribution with random noise. One possibility is that shorter segments have a higher false positive rate than longer segments, as observed in (60, 63). Although the posterior distributions are overlapping, the *D5* Neandertal consistently shows a younger estimated date than the *D17* Neandertal. If the introgression event was shared, this constitutes an independent line of evidence that *D17* lived after *D5* (Table S29).

Conditioning on the most restrictive length cutoff of 0.2 cM, we estimate that *D5* is approximately 284 generations older than *D17* (average across all penalties and maps) which translates roughly to 8,200 years assuming a generation time of 29 years per generation, consistent with the branch shortening estimates (SI Appendix 6).

Denisovan ancestry in other Neandertals

We next investigate whether Denisovan ancestry is also present in later Neandertals, as suggested by recent studies (66, 67). We run *admixfrog* on the high-coverage *Vi33.19* Neandertal from Croatia (8), the high-coverage *Chag8* Neandertal from the Altai mountains (25) and four low-coverage late Neandertals (48). We used the same ascertainment as before but remove *Vi33.19* or *Chag8* from the reference panel when those individuals were the targets.

The overall proportions of Denisovan ancestry in those other Neandertals are much lower than in *D5* and *D17* (Table S30). However, as mentioned earlier, *admixfrog* cannot distinguish segments coming from incomplete lineage sorting from more recent introgression, we focus on longer DNA segments that may suggest admixture.

Similar to above, we called Denisovan introgressed segments using a penalty of 0.4 under the African-American map and a length cut-off of 0.2 cM and at least 10,000 bp. We find one segment in the *Vi33.19* Neandertal with a length of 0.230 cM, close to our 0.2 cM cutoff. In *Chag8*, we identify 7 segments, the longest estimated at 0.595 cM. We could not identify any segments longer than 0.2 cM for *Goyet GN1, Spy94a, LesCottesZ4-1514* or *Mezmaiskaya2* using the African American map (Table S31).

We investigated the state of Denisova-specific SNPs in the longest segments in each genome under the African American map, by plotting them together with the reference (Fig. S45). The segments in *Vi33.19* and *Chag8* show matching to unique Denisovan sites. The segments in the other Neandertals do not show good matching to unique Denisovan sites and might stem from ILS.

Origin of segments identified by *admixfrog*: Denisovan introgression or ILS?

As *admixfrog* cannot distinguish segments resulting from ILS from those resulting from introgression, we test whether the putative Denisovan segments identified in *D17*, *D5*, *Vi33.19* and *Chag8* originate from ILS or introgression by estimating the Time to the Most Recent Common Ancestor (TMRCA) between the putative introgressed segments and Denisovan genomes. Introgressed segments can have a TMRCA younger than the split time whereas segments that stem from ILS can only have TMRCAs older than the split time. The introgression hypothesis is therefore supported when estimated times are younger than the split time between Neandertals and Denisovans.

We estimated the TMRCA following ((68, 69), using the number of pairwise differences *D*. However, because chromosomes are not phased, we do not know if heterozygous alleles are on the putative introgressed Denisovan or Neandertal haplotype. Therefore, we estimate the TMRCA only for putative Denisovan segments that are in a homozygous state, as detected by *admixfrog*. We use all homozygous

segments called with a restrictive penalty of 0.1, minimum genetic length of 0.05 cM on the African-American map and a minimum physical length of 100 kb.

The TMRCA is then modeled as:

$$P(D \mid t) = Pois(Nt)$$

Where *D* is the number of pairwise difference between the putatively introgressed segment and the Denisovan genome, *t* is the total branch length in generations until coalescence between the two genomes and *N* the number of sites on the segment where both genomes have genotype calls. The maximum likelihood estimator for *t* is given by:

$$t=(D/N)\mu$$

We used a mutation rate μ of 1.45e-8 per generation, assuming a generation time of 29 years, which correspond to 0.5e-9 mutations per base pair per year as in (18). We corrected for the sampling time by adding $t_{age\_ind\_1} + t_{age\_ind\_2}$ to *t*. The following ages (in years before present) were used for Denisovans: *D3* = 68,000 (10) and *D25* = 205,000 (61); for Neandertals *Vi33.19* = 49,000; *Chag8* = 78,000; *D5* = 119,000 and *D17* = 110,000. The TMRCA was then calculated as *t*/2. Upper and lower estimates were obtained using the *pois.exact* function from the *epitools R* package (version 0.9) (Fig. S46).

We find that the TMRCAs for the homozygous putative Denisovan segments in *D5* and *D17* Neandertals are younger than the Neandertal-Denisovan split time, while the TMRCAs for segments in *Vi33.19* and *Chag8* are all compatible with ILS (Fig.S46). Although, it was previously suggested that *Chag8* carries Denisovan introgressed segments using one Denisovan reference genome (67), the addition of a second Denisovan genome as reference and the estimation of the TMRCAs suggest that there is not strong evidence for recent, direct admixture with Denisovans. This is further supported by the observation that *Vi33.19* and *Chag8* do not differ in their allele sharing with the Denisovan *(25)*. We conclude that there are no clear evidence of recent direct admixture between Neandertals and Denisovans outside of Denisova Cave.

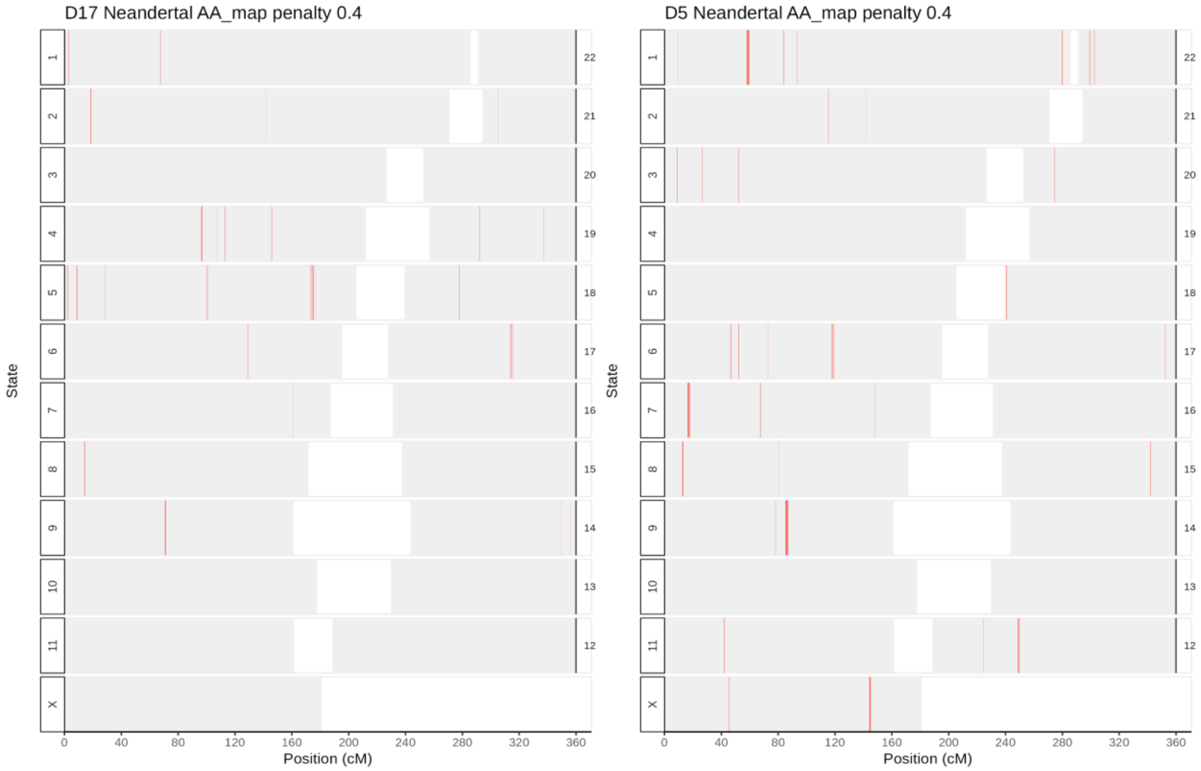

Figure S42. Genomic location of putative Denisovan segments inferred using *admixfrog* with penalty 0.4 and minimum length cutoff of 0.2 cM (red) in the *D5* and *D17* Neandertal genomes.

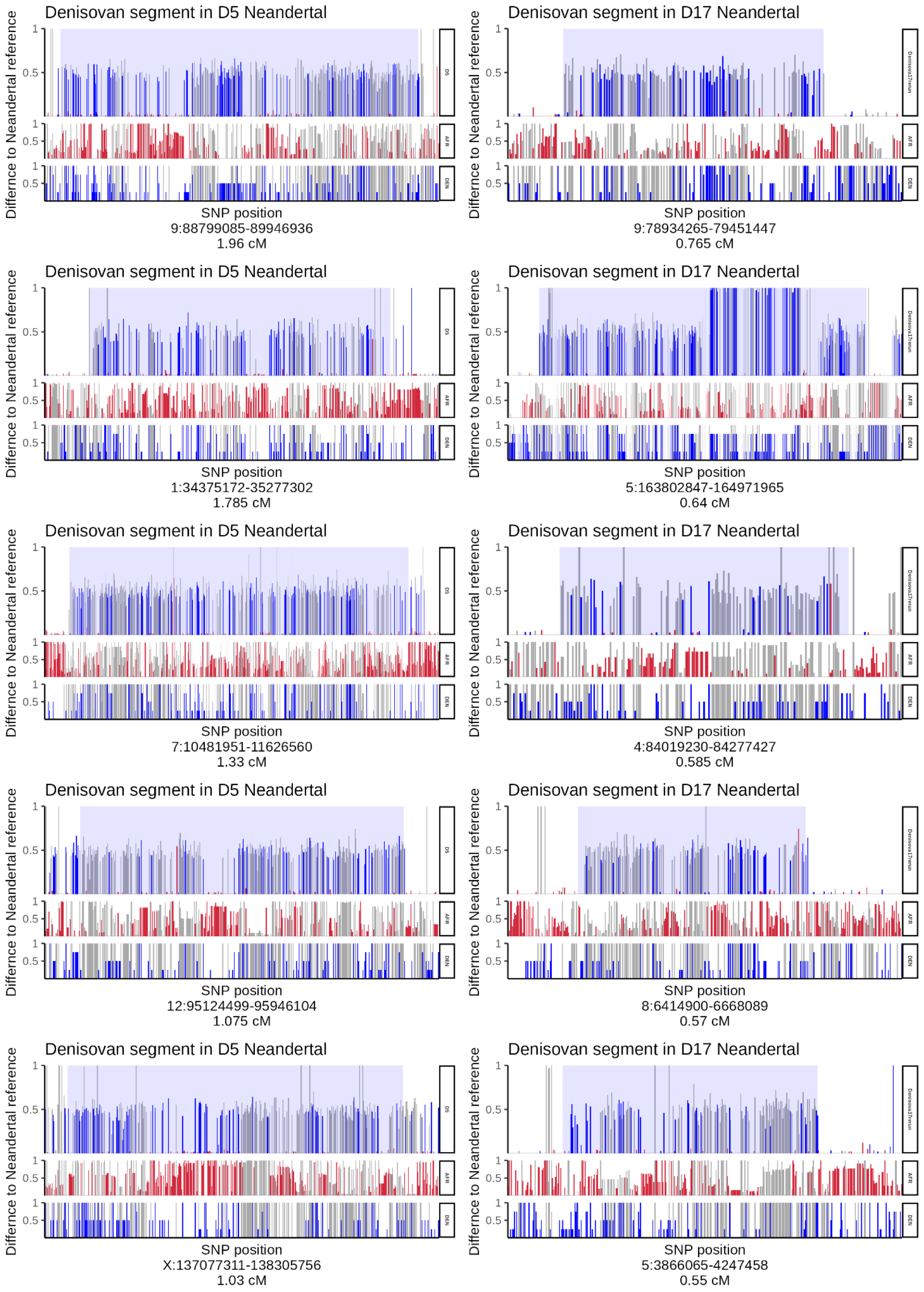

Figure S43. Five longest putatively Denisovan introgressed segments in the *D5* and *D17* Neandertal. Each plot represents an introgressed segment (light blue box) with its position (chrom: start-end) and length in centiMorgan (cM) annotated below the plot. The x-axis spans the segment itself (light blue box) plus flanking region on both sides including diagnostic positions located 100kb upstream and 100kb downstream of the segment. The bar height on y-axis represents the difference in allele frequency between the reference Neandertals and Denisovans (lower subplot), the reference Africans (middle subplot), or the target individual (upper subplot). Bar colors indicate Denisovan alleles (blue) or Africans alleles (red), while grey bars indicate alleles found in multiple references.

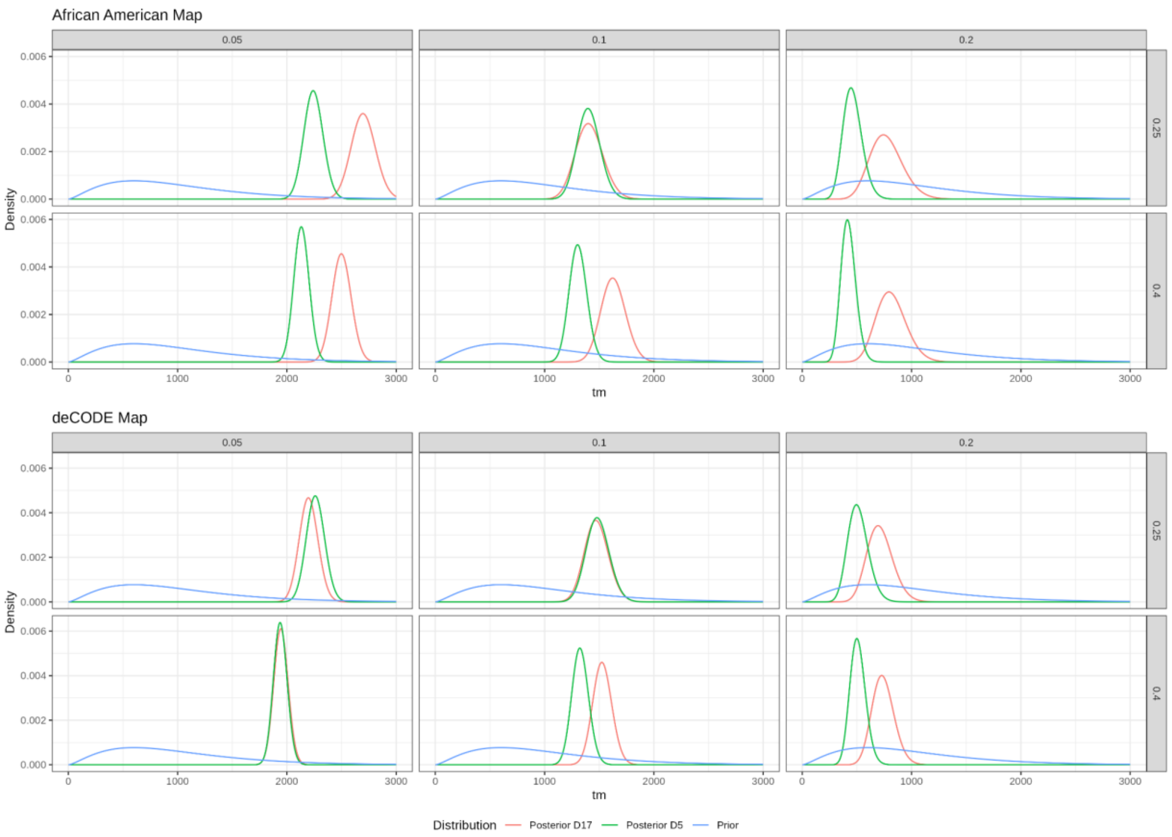

Figure S44. Posterior distribution of the time since Denisovan introgression (t_m) in generations for *D17* (green) and the *D5* Neandertals (red) under two different genetic maps. Blue line indicates the prior distribution of t_m. Upper facets indicate minimum length for the segments in cM. Right facet the penalty used.

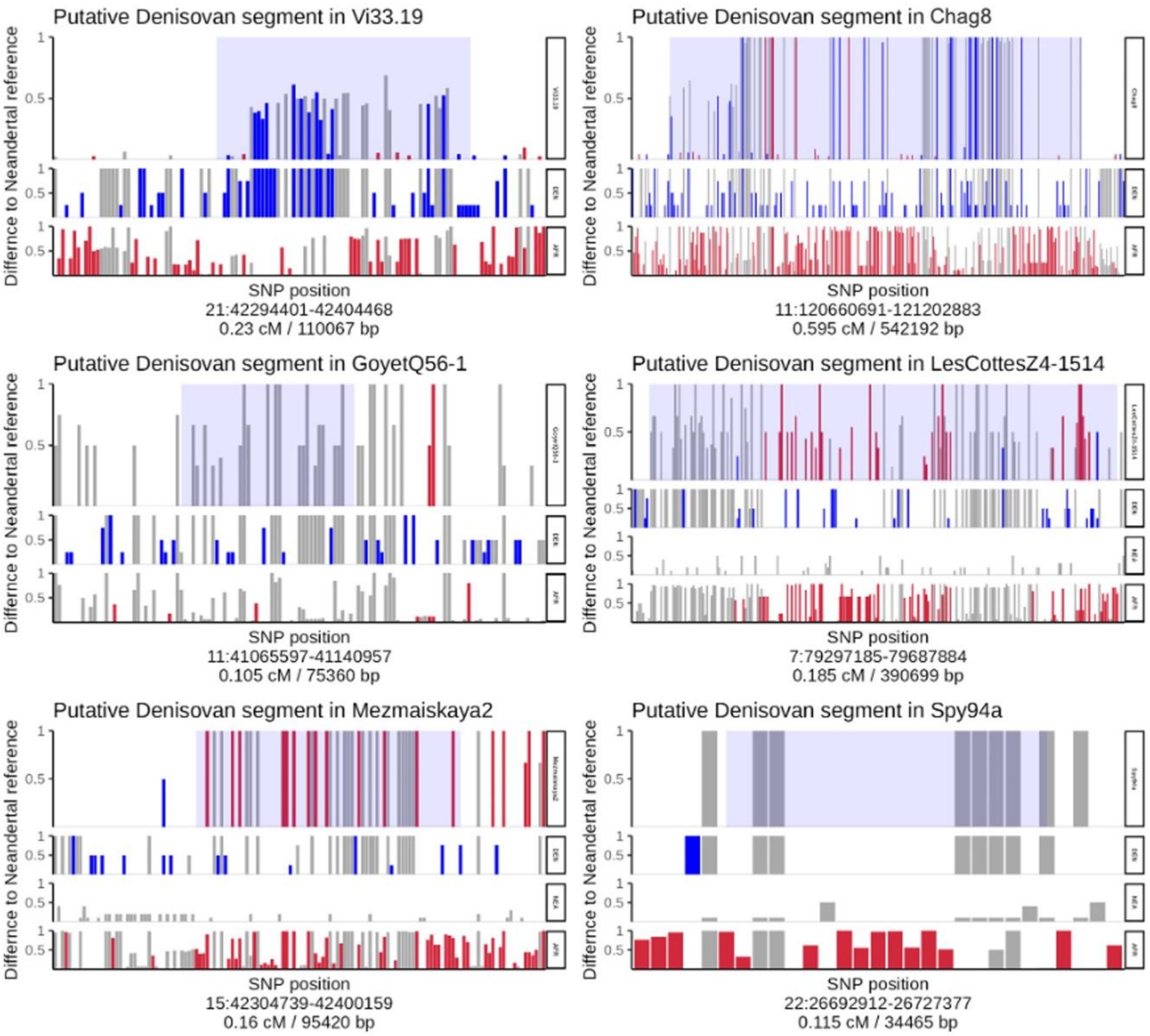

Figure S45. Longest putatively introgressed segments from Denisovans in the Neandertals *Vi33.19 (Vi33.19), Chag8 (Chag8), GoyetQ56-1 (GN1), Spy94a, Mezmaiskaya2 and LesCottesZ4-1514*. Each plot represents an introgressed segment (light blue box) with its position (chrom: start-end) and length in cenitMorgan (cM) annotated below the plot. The x-axis spans the segment itself (light blue box) plus flanking region on both sides including diagnostic positions located 100kb upstream and 100kb downstream of the segment. The bar height on y-axis represents the difference in allele frequency between the reference Neandertals and Denisovans (lower subplot), the reference Africans (middle supplot), or the target individual (upper subplot). Bar colors indicate Denisovan alleles (blue) or Africans alleles (red), while grey bars indicate alleles found in multiple references.

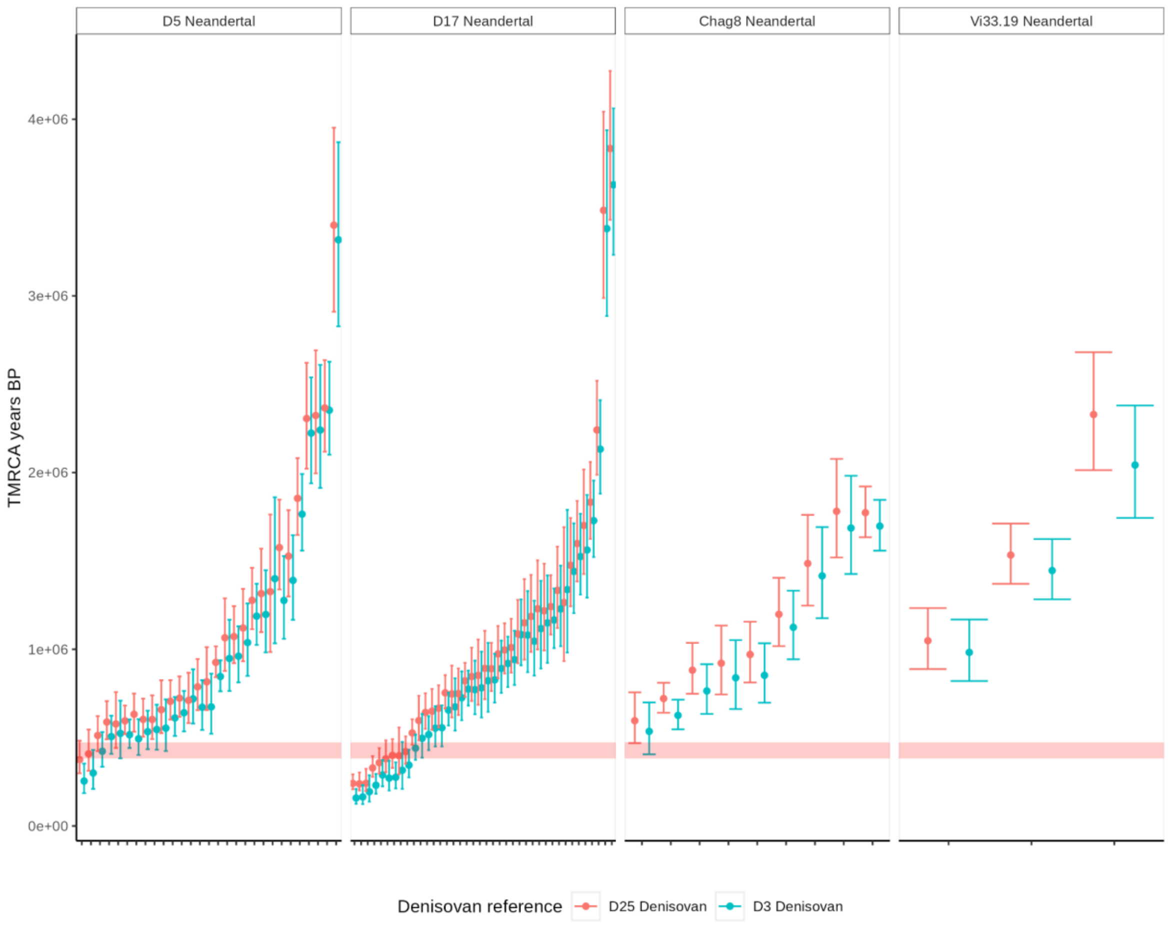

Figure S46. Time to the most recent common ancestor between the putatively introgressed Denisovan segments in Neandertals and two Denisovan genomes. Estimates were obtained using either the African-American or deCODE map with penalty 0.25 or 0.4 and either *D3* (blue) or *D25* (red) as reference. Times are given in years before present. Shaded red area indicates split time between Neandertals and Denisovans.

Table S26. Proportion of putative Denisovan ancestry in the *D5* and *D17* Neandertal genomes estimated using *admixfrog* with two recombination maps.

Individual	Genetic map	Mean proportion	Lower interval	Upper interval
Neandertal D5	AA_map	0.072	0.070	0.073
Neandertal D5	deCODE	0.069	0.068	0.070
Neandertal D17	AA_map	0.070	0.069	0.071
Neandertal D17	deCODE	0.067	0.066	0.068

Table S27. Putative Denisovan segments *D5* and *D17* Neandertal genomes longer than 0.2 cM using different penalties and genetic maps from *admixfrog.*

Individual	Genetic map	Penalty	number of segments >= 0.2 cM	Mean length	Cumulative length in Mb
Neandertal D5	AA_map	0.25	29	0.43	102.24
Neandertal D5	AA_map	0.40	40	0.45	129.64
Neandertal D5	deCODE	0.25	32	0.41	92.53
Neandertal D5	deCODE	0.40	55	0.41	147.12
Neandertal D17	AA_map	0.25	24	0.33	70.58
Neandertal D17	AA_map	0.40	33	0.32	85.21
Neandertal D17	deCODE	0.25	34	0.34	86.18
Neandertal D17	deCODE	0.40	52	0.34	118.00

Table S28. Correlation of Denisovan ancestry in *D17* and the *D5* Neandertal genomes.

Genetic map	Penalty	Correlation	n segments	n overlaps	Total amount in cM	Amount overlapping in cM	Bootstrap mean correlation	Bootstrap sd correlation	Empirical p-value
AA_map	0.25	0.026	54	1	20.40	0.285	-0.00014	0.00805	0.026
deCODE	0.25	0.032	67	2	24.82	0.455	0.00031	0.00875	0.016
AA_map	0.4	0.017	76	1	28.78	0.29	0.00015	0.00837	0.048
deCODE	0.4	0.126	109	11	40.17	2.66	-0.00007	0.00892	0

Table S29. Estimates for the time since admixture in generations.

Mean tm *Den17*	Mean tm *D5*	Lower CI *D17*	Lower CI *D5*	Upper CI *D17*	Upper CI *D5*	Difference Altai vs. *D17*	Posterior_diff_ *D5_D17*_years	n segments *D17*	n segments *D5*	Penalty	Genetic map	min_l
2702	2245	2484	2073	2924	2422	-458	-13268	590	655	0.25	AA_map	0.05
1412	1402	1182	1206	1666	1614	-10	-297	123	177	0.25	AA_map	0.1
771	462	510	309	1092	644	-309	-8965	24	26	0.25	AA_map	0.2
2199	2261	2038	2098	2373	2429	62	1796	660	725	0.25	deCODE	0.05
1476	1486	1270	1287	1690	1706	11	309	180	195	0.25	deCODE	0.1
713	511	504	346	963	707	-202	-5866	34	28	0.25	deCODE	0.2
2502	2132	2331	2001	2674	2274	-370	-10729	812	923	0.40	AA_map	0.05
1629	1308	1417	1154	1858	1470	-322	-9326	205	258	0.40	AA_map	0.1
818	424	575	300	1107	566	-394	-11429	33	37	0.40	AA_map	0.2
1947	1939	1823	1821	2080	2064	-8	-225	884	960	0.40	deCODE	0.05
1529	1327	1367	1180	1701	1478	-202	-5866	307	300	0.40	deCODE	0.1
741	509	559	376	954	654	-231	-6712	52	49	0.40	deCODE	0.2

Table S30. Estimates of the overall proportion of Denisovan ancestry from *admixfrog* for six Neandertals, using two different recombination maps.

Individual	Genetic map	Mean	Upper	Lower
Neandertal *Vi33.19*	AA_Map	0.014	0.015	0.014
Neandertal *Vi33.19*	deCODE	0.011	0.012	0.010
Neandertal *Chag8*	AA_Map	0.026	0.026	0.025
Neandertal *Chag8*	deCODE	0.022	0.023	0.022
Neandertal *GN1*	AA_Map	0.002	0.002	0.001
Neandertal *GN1*	deCODE	0.001	0.001	0.001
Neandertal *LesCottesZ4-1514*	AA_Map	0.008	0.008	0.007
Neandertal *LesCottesZ4-1514*	deCODE	0.006	0.006	0.006
Neandertal *Mezmaiskaya2*	AA_Map	0.008	0.008	0.007
Neandertal *Mezmaiskaya2*	deCODE	0.006	0.007	0.006
Neandertal *Spy94a*	AA_Map	0.003	0.004	0.003
Neandertal *Spy94a*	deCODE	0.002	0.002	0.002

Table S31. List of identified putative Denisovan segments by *admixfrog* in other Neandertals longer than 0.2 cM using different penalties and genetic maps.

Individual	Penalty	Genetic map	Number of segments ≥ 0.2 cM	Mean length cM	Cumulative length Mb
Neandertal *Chag8*	0.10	AA_Map	1	0.34	4.227
Neandertal *Chag8*	0.10	deCODE	3	0.23	8.683
Neandertal *Chag8*	0.25	AA_Map	5	0.25	9.537
Neandertal *Chag8*	0.25	deCODE	9	0.26	18.461
Neandertal *Chag8*	0.40	AA_Map	7	0.31	17.302
Neandertal *Chag8*	0.40	deCODE	17	0.31	39.026
Neandertal *LesCottesZ4-1514*	0.40	deCODE	1	0.20	0.644
Neandertal *Mezmaiskaya2*	0.25	deCODE	1	0.42	6.355
Neandertal *Mezmaiskaya2*	0.40	deCODE	1	0.43	6.460
Neandertal *Vi33.19*	0.10	deCODE	1	0.31	0.930
Neandertal *Vi33.19*	0.25	deCODE	1	0.32	0.969
Neandertal *Vi33.19*	0.40	AA_Map	2	0.24	2.184
Neandertal *Vi33.19*	0.40	deCODE	3	0.33	3.365

SI Appendix 17 – Neandertals and modern human admixture

Leonardo N. M. Iasi, Hugo Zeberg

Potential early modern human ancestry in *D5* and *D17*

Gene flow from early modern humans into Neandertals has been suggested (18, 19, 70–72). We investigate whether early modern human ancestry can be detected in the *D5* and *D17* genomes using *admixfrog* with the same ascertainment and reference panel as in SI appendix 16.

The command used for *admixfrog* was:

```
admixfrog-bam --ref ref.file --bamfile input.bam --force-bam --deam-cutoff 3 --out output.in.xz --length-bin-size 15

admixfrog --infile  output.in.xz  --ref ref.file -o out.name \
--states NEA DEN AFR  --cont-id AFR --ll-tol 1e-2 --bin-size 5000 \
--est-F --est-tau --freq-F 3  --freq-contamination 3 --e0 1e-2  --est-error \
--ancestral PAN  --run-penalty 0.1 --max-iter  250 --n-post-replicates 200 \
--filter-pos 50 --filter-map 0.000  --init-guess NEA

admixfrog-rlt --out output.rle.xz –in input.bin.xz --run-penalty 0.4
```

Depending on the recombination map used, we estimated putative modern human ancestry in both genomes to be around 3% (Table S32). We called the putative introgressed modern human segments longer than 0.2cM in each genome and found that these segments are, on average, shorter than those introgressed from Denisovans (Table S33). Figure S47 shows the five longest segments in the *D17* and *D5* genomes and how the frequency of reads matches the allele frequency in the reference populations.

Only one segment in each of the *D5* and *D17* genomes appear to be relatively well supported along almost the entire length of the estimated introgressed region (*e.g.*, on chromosome 14 in *D17* and chromosome 1 in *D5*). In some cases, the frequency of reads carrying the allele matching the modern human state is very low or nearly zero (*e.g.*, *D17* chromosomes 10, 4 and 5).

When comparing segments called as modern human, longer or equal to 0.2 cM, in the *D5* genome using the African-American map and penalty of 0.4 with segments previously identified as modern human introgressed using *ARGweaverD (72),* four out of nine segments identified here overlap with segments found in the previous study (72). Overall, we found a few well-supported segments and an overlap with previous identified ones, consistent with an admixture between Neandertals and early modern humans older than 100,000 years before *D5* lived.

Affinities of Neandertal ancestry in present-day genomes to Neandertal genomes

To investigate the relative affinities of Neandertal introgressed genomic segments in present-day non-Africans to the high-coverage Neandertal genomes, we identified Neandertal tracts using HMMix (73) on

the combined HGDP (74) + 1000 Genome Project dataset (56) (n= 4,091; gnomAD v3.1.2). Tracts were inferred using default parameters, specifying Sub-Saharan African populations as outgroups to represent non-admixed modern human variation.

For each introgressed tract, we counted the number of single-nucleotide variants carrying an Neandertal allele that matched each high-coverage genome, *Vi33.19*, *GN1*, *Chag8*, *D5* and *D17*. Each tract was assigned to the archaic genome with the highest number of matching variants, excluding cases with equal counts.

This approach provides a coarse-grained estimate of the likely archaic source of introgressed fragments and allows comparison of their relative representation across modern populations. The total number of tracts assigned to each Neandertal genome was subsequently summed to obtain the counts shown in Fig. S48.

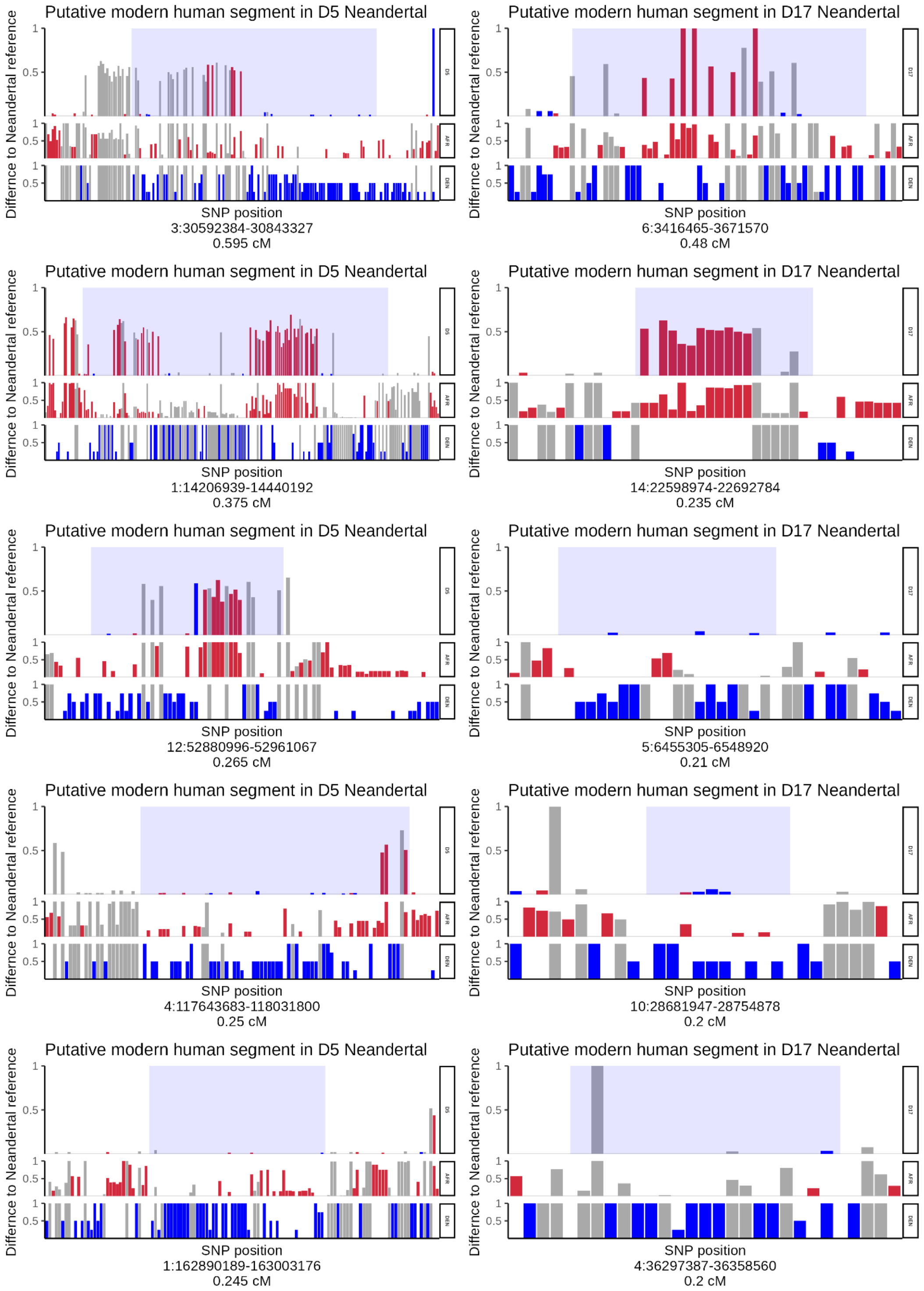

Figure S47. Five longest putatively introgressed segments from modern humans in the *D5* and *D17* Neandertal. Each plot represents an introgressed segment (light blue box) with its position (chrom: start-end) and length in centiMorgan (cM) annotated below the plot. The x-axis spans the segment itself (light blue box) plus flanking region on both sides including diagnostic positions located 100kb upstream and 100kb downstream of the segment. The bar height on y-axis represents the difference in allele frequency between the reference Neandertals and Denisovans (lower subplot), the reference Africans (middle subplot), or the target individual (upper subplot). Bar colors indicate Denisovan alleles (blue) or Africans alleles (red), while grey bars indicate alleles found in multiple references.

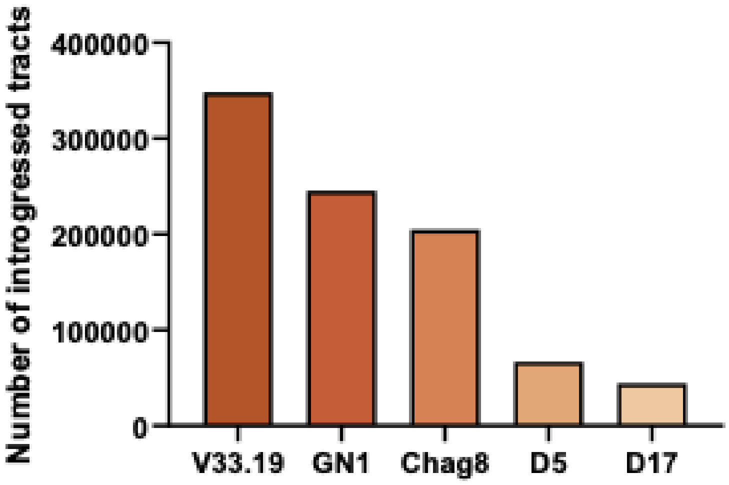

Figure S48. Comparative overview of the relative affinities of Neandertal introgressed segment in present-day non-Africans to different Neandertal individual. Bar chart showing the total number of introgressed tracts assigned to each high-coverage Neandertal genome (*Vi33.19, GN1, Chag8, D5,* and *D17*).

Table S32. Overall modern human and Denisovan ancestry estimates from *admixfrog* for the *D5* and *D17* Neandertals using two different recombination maps.

Individual	Genetic map	Mean AFR	Lower AFR	Upper AFR	Mean DEN	Lower DEN	Upper DEN
Neandertal *D5*	AA_map	0.037	0.036	0.037	0.048	0.047	0.049
Neandertal *D5*	deCODE	0.036	0.035	0.037	0.046	0.045	0.047
Neandertal *D17*	AA_map	0.033	0.032	0.034	0.048	0.047	0.049
Neandertal *D17*	deCODE	0.033	0.033	0.034	0.045	0.044	0.046

Table S33. Summary of Denisovan and modern human ancestry segments in *D5* and *D17* Neandertals for segments equal or longer 0.2 cM.

Individual	Penalty	Genetic map	Ancestry	Number of segments $\geq$ 0.2 cM	Mean length in cM	Cumulative length in Mb
Neandertal *D5*	0.25	AA_map	AFR	2	0.25	5.74
Neandertal *D5*	0.25	AA_map	DEN	15	0.41	54.20
Neandertal *D5*	0.25	deCODE	AFR	0	0.00	0.00
Neandertal *D5*	0.25	deCODE	DEN	19	0.44	71.32
Neandertal *D5*	0.4	AA_map	AFR	9	0.29	14.45
Neandertal *D5*	0.4	AA_map	DEN	22	0.50	98.58
Neandertal *D5*	0.4	deCODE	AFR	12	0.23	28.58
Neandertal *D5*	0.4	deCODE	DEN	21	0.62	97.73
Neandertal *D17*	0.25	AA_map	AFR	2	0.22	1.99
Neandertal *D17*	0.25	AA_map	DEN	12	0.35	49.42
Neandertal *D17*	0.25	deCODE	AFR	3	0.28	5.28
Neandertal *D17*	0.25	deCODE	DEN	15	0.41	52.36
Neandertal *D17*	0.4	AA_map	AFR	6	0.25	6.43
Neandertal *D17*	0.4	AA_map	DEN	18	0.36	64.28
Neandertal *D17*	0.4	deCODE	AFR	8	0.25	10.18
Neandertal *D17*	0.4	deCODE	DEN	27	0.37	78.84

SI Appendix 18 – The Y chromosome of the Neandertal *D17*

Yaniv Swiel

Data processing

DNA sequences mapped to the Y chromosome were processed as described in SI Appendix 3. After the application of the *map35_100* filter (18), a total of 2,688,353 confidently aligned sequences were retained for downstream analyses. The read length distribution (Fig. S49) and the DNA deamination patterns (Fig. S50, Table S34) of the Y chromosome sequences are similar to those observed in the rest of the genome.

As described in SI Appendix 3, genotypes were called with *snpAD (15)*. However, as *snpAD* is a diploid genotyper and heterozygous positions are not expected outside of the pseudoautosomal regions of the Y chromosome, we converted 95 heterozygous genotype calls to homozygous calls based on the lowest homozygous posterior probability calculated by *snpAD*. As previously described in SI Appendix 3, we applied GC-based coverage filters to exclude positions below the 0.5% percentile and above the 97.5% percentile of each GC-dependent coverage distribution. After removing tandem repeats and repetitive regions that are not removed by the *map35_100* filter by applying the *capture_full* filter (71), we retain a total of 4.45 million confidently called genotypes positions. The mean coverage of the final set of Y chromosome genotypes is 18.8x with an average genotype quality of 94.2.

Comparative Y chromosome dataset

We compiled a dataset (Table S35) including Y chromosomes from *28* present-day humans, 4 ancient modern humans and 4 Neandertals. In order to represent the variation among present-day human Y chromosomes, we selected 15 non-African and 10 African individuals from the Human Genome Diversity Project (75). We also included two Y chromosomes (representing haplogroups A0a1 and A1a) from the 1000 Genomes Project (76) as well as two Y chromosomes representing the A00 haplogroup (*i.e*., the most basal modern human lineage) (77). As the two A00 Y chromosomes are relatively low coverage, but are derived from closely related individuals, their sequence data was merged into a single BAM file as done in the original study (77). The dataset also contains high coverage shotgun-sequenced Y chromosomes from radiocarbon-dated ancient modern humans *Ust'Ishim* (26), *Loschbour* (27), *Yana 1* (78) and an individual from Shum Laka (2/SE II) carrying the A00 haplogroup (79). We also included capture sequenced Y chromosomes from the Neandertals *Mezmaiskaya 2* (15.8x coverage) (71), *Spy94a* (2) (1.7x coverage) (71) and *Chagyrskaya 2* (46.8x coverage) (67), obtained with a ~6.9 Mb Y chromosome capture probe set, as well as the capture sequenced Y chromosome of the *El Sidrón 1253* Neandertal (7.9x coverage) (80) obtained with a smaller 560 kb Y chromosome capture array.

Data from the HGDP and 1KGP individuals were processed by first flagging unaligned reads adapter sequences with *MarkIlluminaAdapters* from Picard tools (v2.18.29) (81) and then aligning them to hg19 with BWA-MEM (v0.7.17) (82, 83). Unaligned reads of the ancient individual from Shum Laka were

aligned to hg19 using BWA (v0.4.9) with ancient parameters (-n 0.01 -o 2 -l 16500). We then used the data processing pipeline described for the *D17* Y chromosome to process the Y chromosome data of all of the individuals in our dataset, using only positions between the 2.5% percentile and the 97.5% percentile of each GC-dependent coverage distribution (SI Appendix 3). Due to the low coverage of *Spy94a* and *El Sidrón 1253*, for both datasets we only removed sites with coverage higher than the 99% percentile of the respective GC-dependent coverage distribution.

Finally, in order to identify ancestral alleles, we extracted chimpanzee (*panTro6*) Y chromosome sequences that align to the *hg19* Y chromosome from the UCSC *hg19-panTro6* multiple alignment file from which we 'called' *panTro6* genotypes from the alignment to *hg19*.

Estimating modern human contamination

We estimated the proportion of modern human male DNA contamination in the sequences of the *D17* Y chromosome by determining the frequency of modern human-derived alleles at positions where other Neandertals carry the ancestral allele. To define positions informative about modern human contamination, we identified sites where both *Chagyrskaya 2* and *Mezmaiskaya 2* carry the chimpanzee allele while all present-day humans carry the allele seen in the *hg19* reference genome. This resulted in a set of 402 positions (comprising 141 transversions and 261 transitions).

We computed a contamination estimate using the aligned sequences of the *D17* Y chromosome (Table S36). To minimize errors caused by DNA damage, we do not count a forward-oriented sequence if one of the informative alleles is a C or a reverse-oriented sequence if one of the informative alleles is a G. Using this approach we estimate that 1.72% of all sequences could derive from modern human contamination. Using AuthentiCT (23), a tool that estimates modern human contamination based on DNA damage patterns, on the same data set, we obtained a slightly lower contamination estimate of 0.91% ± 0.896 for all reads.

We then estimated modern human contamination based on the genotype calls of the *D17* Y chromosome. We were able to confidently call *D17* genotypes at 391 of the Neandertal Y chromosome contamination-informative sites, of with 385 matching the ancestral allele, resulting in a genotype-based contamination estimate of 1.53% (95% binomial CI 0.32 - 2.75). If only transversions are considered, the modern human allele is found at one of the 139 *D17* genotypes leading to a contamination estimate of 0.72% (95% CI 0 - 2.12).

Missense variants

We used the Ensembl Variant Effect Predictor (VEP; tool version 107, database version 107) (84) to identify missense variants in the *D17* Y chromosome based on the *GRCh37* annotation database v107. A total of five variants in four genes (Table S37) were identified and classified as having MODERATE impact. All five of the variants are found in present-day populations (gnomAD database v4.0.0). Only one of them, rs7067496, is found at appreciable frequency (allele frequency = 7.8%) while the others are rare variants

(allele frequency < 0.01%). No clinically significant phenotypes are reported in ClinVar for the detected variants. As a comparison, Table S38 reports the ancestral (chimp) allele along with the allele found in the Y chromosomes of the other Neandertals and in the Y chromosomes of two Denisovans (*Denisova4* and *Denisova8*).

Relationship between Neandertal Y chromosomes

To resolve the relationships amongst the Neandertal Y chromosomes we first used genotype calls to determine the relationship among the three Y chromosomes for which we have better coverage (*D17*, *Chagyrskaya2* and *Mezmaiskaya2*). We defined a set of 840 diagnostic positions where the *D17* allele differs from both the human reference allele and the chimpanzee allele. At these positions, we counted the number of sites where one of *Mezmaiskaya 2* or *Chagyrskaya 2* carries the derived allele while the other carries the ancestral allele (Table S39) and found only one shared derived allele between *D17* and *Mezmaiskaya 2*, whereas *Chargyrskaya 2* shares none. This suggest that *D17* Y chromosome is an outgroup to both *Mezmaiskaya 2* and *Chagyrskaya 2 Y chromosomes.* This is further supported by the observation that when diagnostic positions are defined based on the *Chagyrskaya2* Y chromosome, 99 out of 900 such sites are shared with *Mezmaiskaya2*.

We then resolve the Y chromosomal relationships among Neandertals by defining diagnostic sites for each of *Chagyrskaya2*, *Mezmaiskaya2 and D17* Y chromosomes, where one carries a derived allele while the others carry the ancestral allele (*i.e*., the allele matching both the chimpanzee and the human reference). We next estimated for the lower-coverage Neandertal Y chromosomes of *Spy94a* and *El Sidrón 1253,* the proportion of sequences in which the allele matches the derived allele for each of the three high-coverage Y chromosomes (Table S40). To minimize deamination-induced errors, we disregard a sequence if one of the informative alleles is a C when the sequence is in the forward orientation or a G when the sequence is in the reverse orientation. We find that the *D17* Y chromosome represents an outgroup to all previously available Neandertal Y chromosome sequenced, with *Spy94a* being more closely related to *Mezmaiskaya 2* than to *Chagyrskaya 2*, while *El Sidrón 1253* is more closely related to *Chagyrskaya 2* than to *Mezmaiskaya 2*.

Estimating the TMRCA of Neandertal and modern human Y chromosomes

We estimated the time to the most recent common ancestor (TMRCA) among the Neandertal and modern human Y chromosome using all five available Neandertal Y chromosome sequences (*D17, Chagyrskaya2, Mezmaiskaya2, El Sidrón1253,* and *Spy94a*) together with both ancient and present-day modern human sequences (Table S35). We followed the method described in Swiel *et al*., 2025 (85), which mitigates reference bias that affect the branch lengths of both the most divergent modern human Y chromosome

haplogroups (A and B) and Neandertal haplogroups by restricting the analysis to relatively slowly evolving regions of the Y chromosome, as determined by the divergence between humans and chimpanzees (85).

We first determined the modern human Y chromosome mutation rate using the sequence of the 45,000-year-old *Ust'Ishim* individual from Siberia, by counting mutations missing on the *Ust'Ishim* Y chromosome in comparison to present-day non-African Y chromosomes. We estimate a mutation rate of the complete mappable Y chromosome of 7.14×10^{-10} (95% CI 4.92×10^{-10} to 9.70×10^{-10}) mutations/bp/year while the mutation rate of the more conserved regions of the Y chromosome (based on human-chimpanzee sequence divergence) was 6.64×10^{-10} (95% CI 4.12×10^{-10} to 9.57×10^{-10}) mutations/bp/year. To determine the time since the divergence of 2 Y chromosome sequences, whether between Neandertals and modern human or among modern human, we counted the number of derived mutations that accumulated on each sequence since they split, using the chimpanzee (*panTro6*) Y chromosome to identify the ancestral state. Finally, we converted the counts of derived mutations into a TMRCA estimate using the mutation rate. Confidence intervals were computed using a bootstrapping approach by repeatedly sampling derived mutation counts from a Poisson distribution with the λ parameter set to the observed derived mutation count. We then calculated the TMRCA for each resampling and generated a simulated TMRCA distribution from which we computed 95% confidence intervals.

Reference bias can distort phylogeny by making sequences appear artificially closer to, or more distant from, the reference genome, thereby shortening or inflating branch lengths in the tree(85). If reference bias is not accounted for when performing this analysis, the TMRCA estimates differ based on whether the A00 Y chromosome or a non-African Y chromosome is used. Furthermore, the TMRCA estimate for the low-coverage *Spy94a* Y chromosome (Fig. S53) is older than the TMRCAs computed for the higher coverage Neandertal Y chromosomes presumably due to genotyping errors (Fig. S52). After correcting for the effect of reference bias by restricting to the conserved regions, we estimate a modern human-Neandertal Y chromosomal TMRCA around 400ka using either the non-African or the A00 branch (Fig. S51, S52). Reliable TMRCA estimate for *Spy94a* could not be achieved without a minimum coverage cutoff (Figure S53). With a minimum coverage filter of at least 2x, however, the modern human-*Spy94a* TMRCA is consistent with the TMRCAs estimates with the other Neandertal Y chromosome sequences, but, as observed also with El Sidron, the limited number of sites covered result in wider confidence intervals when using these 2 sequences (Table S41).

The molecular age of *D17* based on Y chromosome divergence

We estimated the age of the Neandertal D*17* Y chromosome by calculating the difference in the number of derived Y-chromosomal mutations between *D17* and present-day humans (Fig. S54). We then converted this difference into an age estimate using the previously determined human Y chromosome mutation rate, obtaining an age of approximately 95,000 years.

Phylogenetic Analysis

Using the Y chromosome dataset filtered for human-chimpanzee divergence, we constructed a Y chromosome phylogeny with BEAST2 (v2.6.7) (86) which allowed us to estimate the age of the *D17* Y chromosome as well as the modern human-Neandertal TMRCA and the TMRCA between *D17* and the other Neandertals. *Spy94a* and *El Sidrón 1253* were not included in this analysis due to the low amount of data available for their Y chromosomes.

As BEAST requires a FASTA file as input, we used a custom Python script to convert an input VCF file to FASTA format. Although we included only variable sites to reduce the computational complexity, we edited the BEAST .xml file to specify the nucleotide composition of the invariant sites. To estimate the best-fitting nucleotide substitution model for our dataset (Table S42), we used the BEAST2 package bModelTest (87) which averages over all possible nucleotide substitution models while constructing the phylogeny.

The tip dates of all modern samples were set to zero and uniform priors were used to specify the ages of the ancient samples. We used the confidence intervals of the respective IntCal20 calibration curves (88) to define the range of the priors for the radiocarbon-dated samples and we used wide priors to estimate the molecular ages of *D17* and *Chagyrskaya2* Y chromosomes. The starting tip dates and the ranges of the tip date priors are defined in Table S43. The initial Y chromosome mutation rate was set to $7.34x10^{-10}$ mutations/bp/year as determined in (71), but we set a wide uniform prior of $4x10^{-10}$ to $10x10^{-10}$ mutations/bp/year to allow BEAST to estimate the Y chromosome mutation rate.

To select the best-fitting tree and clock models for our dataset, we used the BEAST2 MODEL_SELECTION package to run a path sampling analysis on four model combinations:

- Strict clock, constant population size
- Relaxed log-normal clock, constant population size
- Strict clock, Coalescent Bayesian Skyline
- Relaxed log-normal clock, Coalescent Bayesian Skyline

The log marginal likelihood for each model combination was estimated using 50 path steps, a value of 0.3 for the alpha parameter used to space out the steps, and a chain length of 10 million MCMC iterations for each step. We used an 80% burn-in for each chain and a pre-burn-in stage of 5 million iterations. Table S44 shows the marginal likelihood for each model combination as determined by the path sampling analysis. The model combinations with the highest support are those implementing the Coalescent Bayesian Skyline population model. As the support for the model combination with the relaxed log normal clock is not significantly better than the strict clock (Bayes factor = 0.49), we constructed the Y chromosome phylogeny using the simpler strict clock model together with the Coalescent Bayesian Skyline tree model.

To construct the Y chromosome phylogeny, we ran four independent MCMC chains of 55 million iterations with a 10 million iteration pre-burn-in stage for each chain. Each chain was sampled every 5,000 iterations and logcombiner was used to combine the resulting log and tree files. Finally, TreeAnnotator was used to generate a single best-supported tree from the combined tree file (Fig. S55) using a 10% burn-in.

The best-supported nucleotide substitution model based on bModelTest was the unnamed 123421 model with the relative nucleotide substitution rates shown in Table S42. The Y chromosome mutation rate was estimated to be 6.75×10^{-10} (95% Highest Posterior Density (HPD) Interval: 6.03×10^{-10} to 7.50×10^{-10}) mutations/bp/year which is consistent with the mutation rate of the more conserved regions of the Y chromosome (based on human-chimpanzee sequence divergence) of 6.64×10^{-10} (95% CI 4.12×10^{-10} to 9.57×10^{-10}) mutations/bp/year estimated above. We estimate that the Neandertal-modern human TMRCA to 395ka (95% HPD Interval: 351- 442ka) and that *D17* lived around at 121ka (95% HPD Interval: 100-142ka). Considering the large confidence intervals, these estimates are consistent with those obtained using the branch shortening method described above (around 400ka for the Neandertal-modern human TMRCA and around 97ka for *D17*). We find that, as proposed above, *D17* is an outgroup to *Chagyrskaya 2* and *Mezmaiskaya 2* with a Y chromosome TMRCA of 166ka (95% HPD Interval: 145-187ka). Finally, we estimate the TMRCA of the *Chagyrskaya 2* and the *Mezmaiskaya 2* Y chromosomes to be around 121ka (95% HPD Interval: 105-137ka).

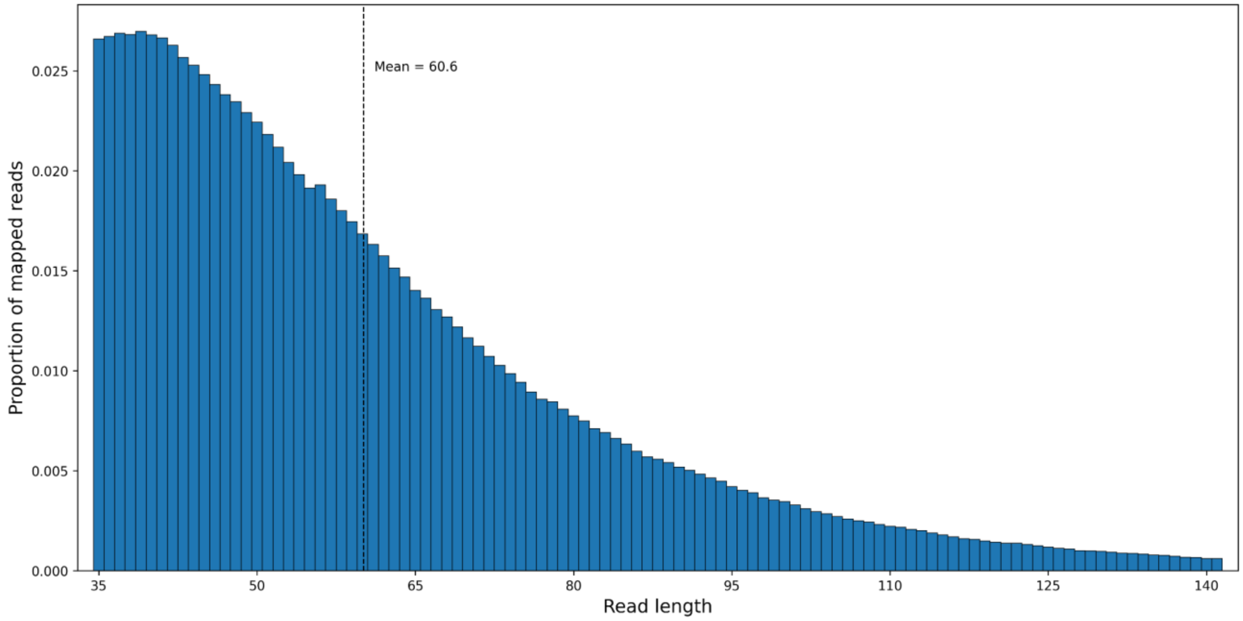

Figure S49. Length distribution of the *D17* Y chromosome sequences that passed the mapping and quality filters.

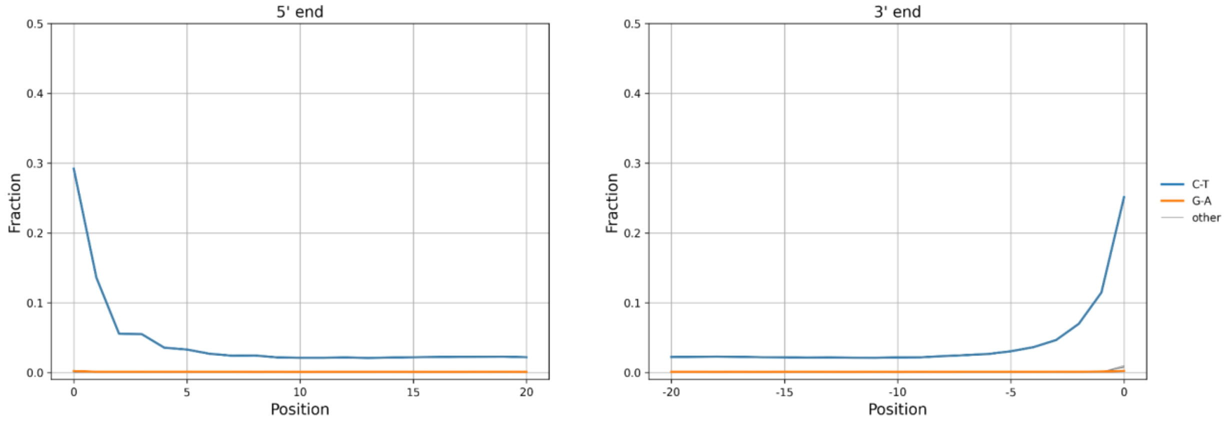

Figure S50: Ancient DNA deamination patterns of the *D17* Y chromosome sequences used to call genotypes.

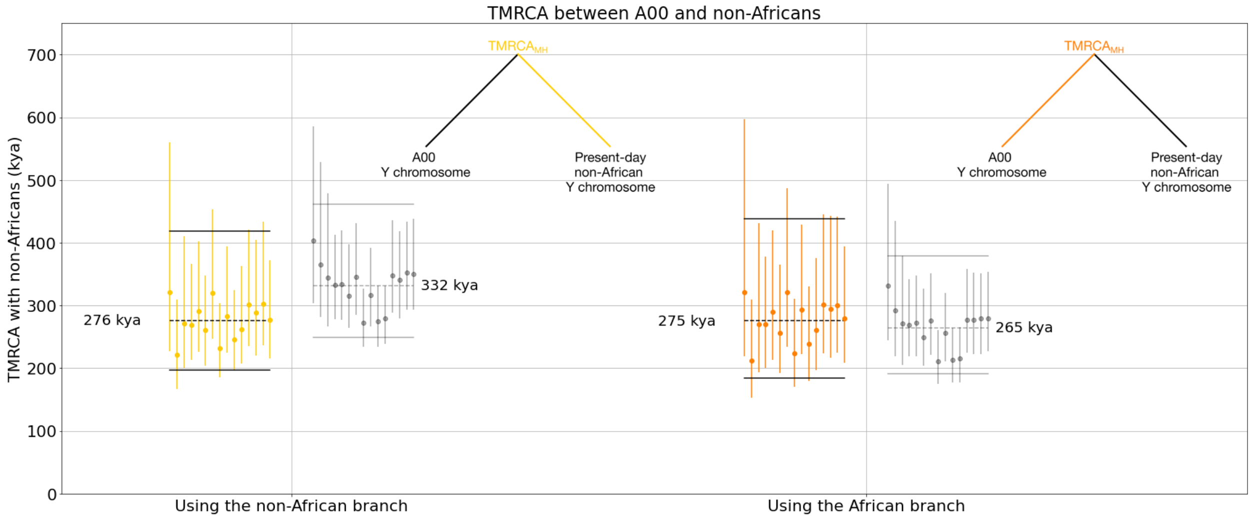

Figure S51: TMRCA estimates between a present-day A00 Y chromosome and 15 present-day non-African Y chromosomes. The plots in colour represent the TMRCAs calculated with the filtered Y chromosome where the effect of reference bias is minimised, while the plots in grey represent the MRCAs estimated using the full Y chromosome. Each dot denotes the TMRCA point estimate with a single present-day non-African Y chromosome, and the error bars represent 95% CIs computed using the resampling method described above. The dotted horizontal lines show the mean TMRCA calculated overall non-African Y chromosomes and the solid horizontal lines represent the overall 95% CIs.

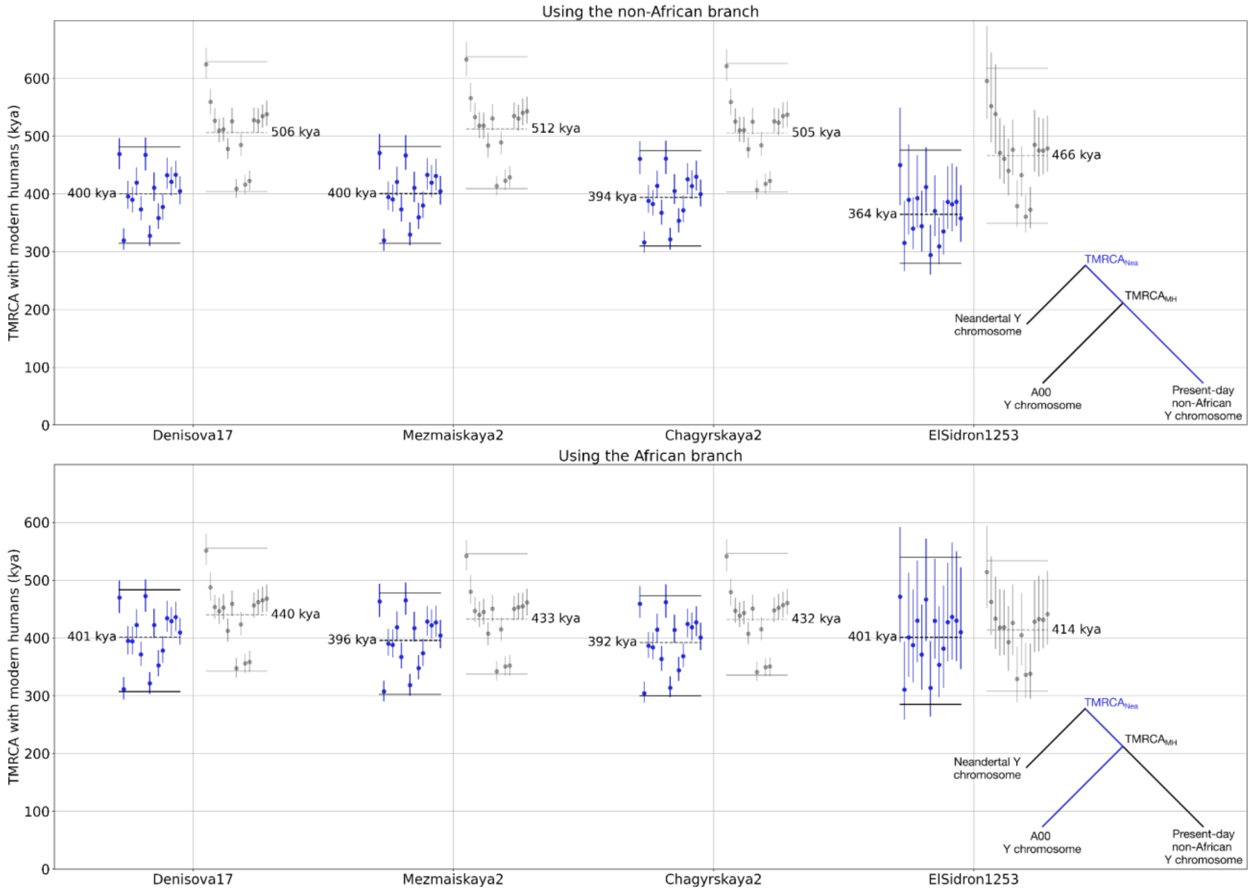

Figure S52. Y chromosome TMRCA estimates between Neandertal Y chromosomes and 15 present-day non-African Y chromosomes.

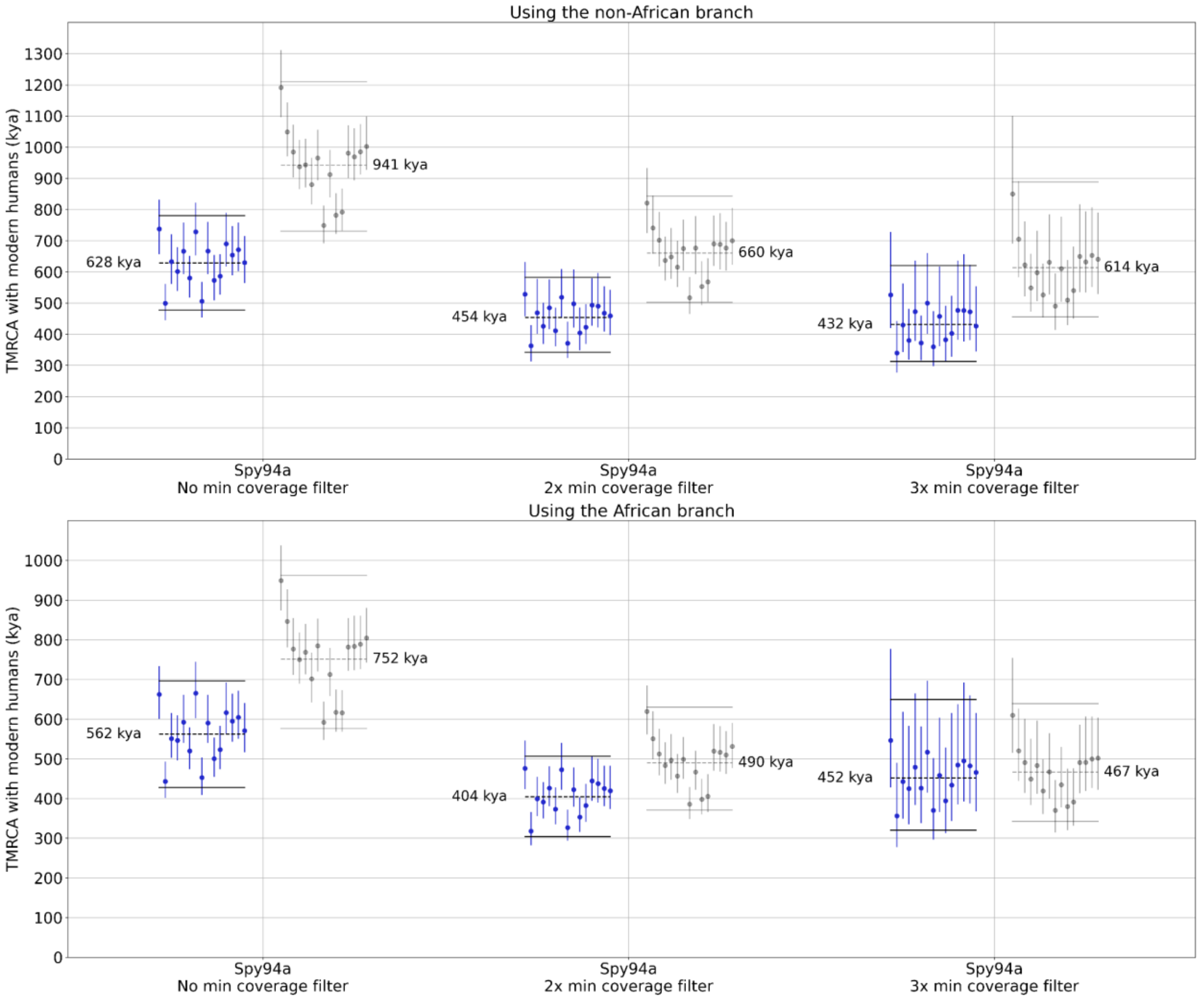

Figure S53. Y chromosome TMRCA estimates between the *Spy94a* Y chromosome (using different minimum coverage filters) and 15 present-day non-African Y chromosomes.

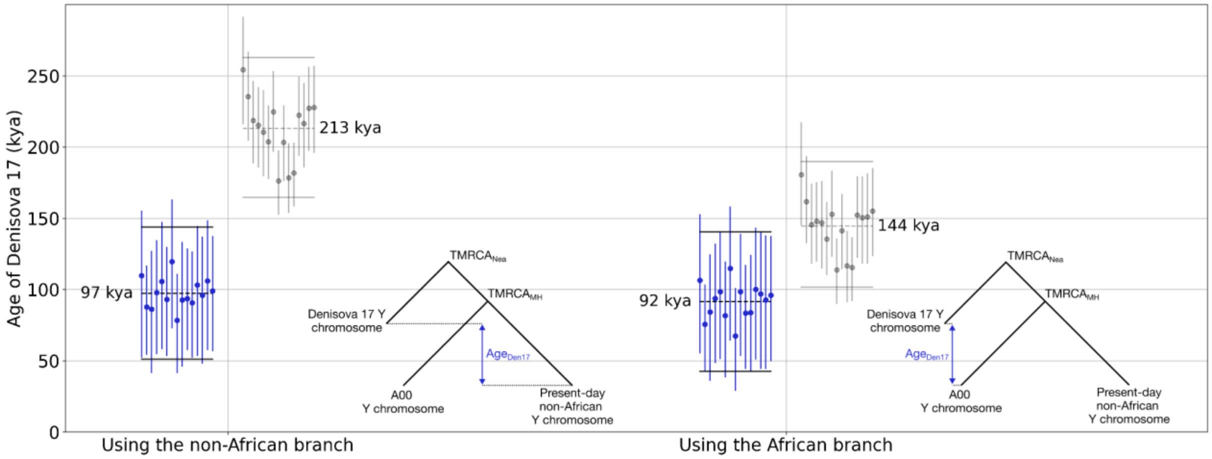

Figure S54. Estimate of the age of *D17* based on the number of mutations missing on his Y chromosome compared to those of present-day humans.

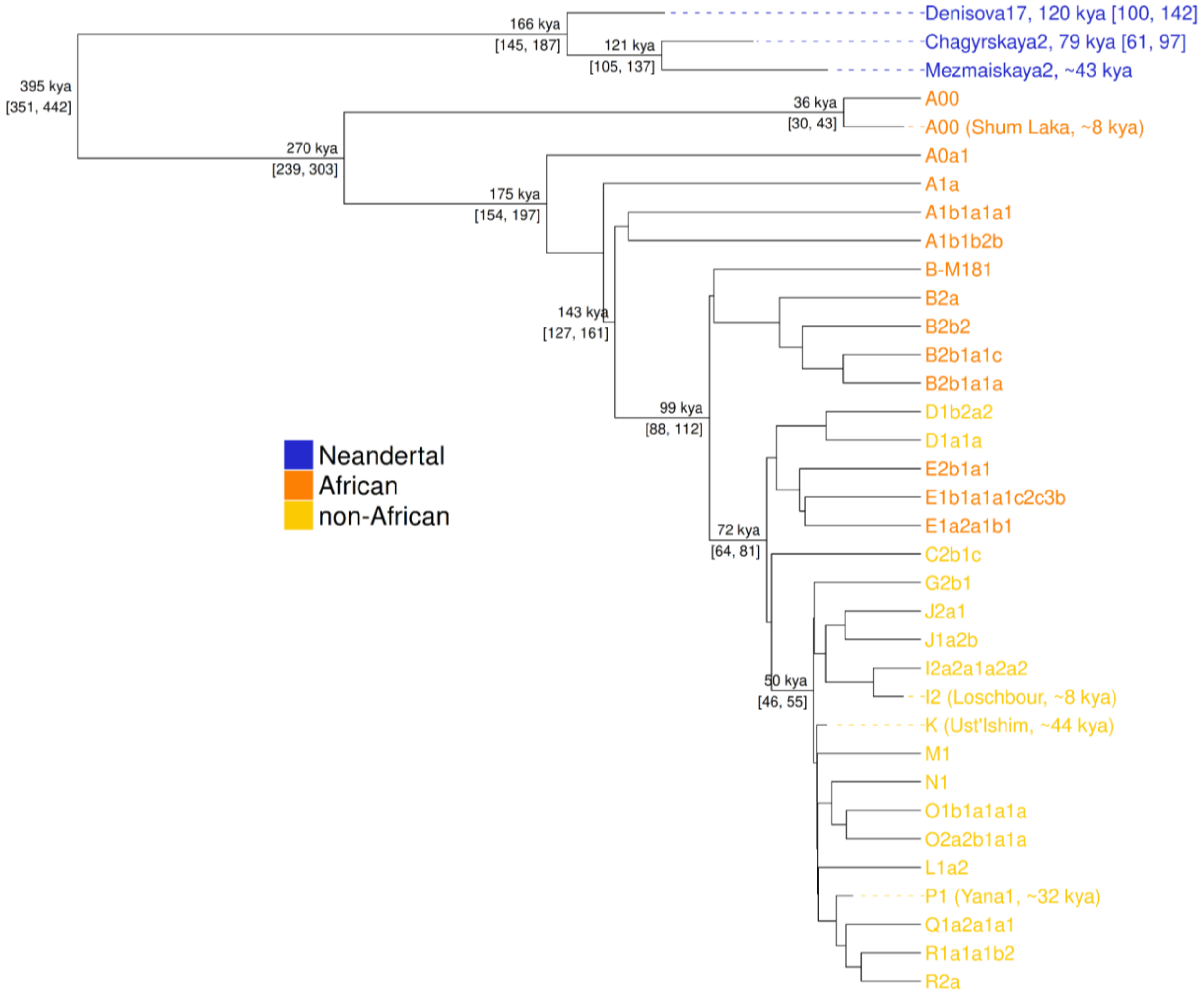

Figure S55. Y chromosome phylogeny constructed by BEAST2. The estimated TMRCAs and the age estimates for *D17 (Denisova 17)* and *Chagyrskaya2* are based on the mean of their respective posterior probability distributions, while the numbers in brackets denote 95% Highest Posterior Density intervals. Dated ancient individuals are marked with calibrated (IntCal20) radiocarbon dates.

Table S34. Number of uniquely mapped sequences passing the quality filters along with the proportion of sequences with terminal C-to-T substitutions within the first three or last three positions.

Mapped sequences	Mean sequence length	Sequences with terminal C-to-T substitutions	% sequences with terminal C-to-T substitutions
2,688,353	60.6	454,023	16.89

Table S35. Human Y chromosomes included in the comparative dataset.

Individual	Data Source	Sample accession	Haplogroup	Age (years BP)
Chagyrskaya2	(10)	SAMEA110640443	Neandertal	64000 (48000–83000)
Mezmaiskaya2	(2)	SAMEA7062760	Neandertal	43449 (42156–44965) [IntCal20]
Spy94a	(2)	SAMEA7062761	Neandertal	40922 (40445–41381) [IntCal20]
El Sidrón 1253	(11)	SAMEA2061884	Neandertal	49000 (46000–53000) [IntCal13]
Shum Laka (2/SE II)	(9)	SAMEA6104836	A00	7913 (8008–7839) [IntCal20]
Loschbour	(7)	SAMEA2697124	I2	8025 (7936–8171) [IntCal20]
Ust'Ishim	(6)	SAMEA2656891	K	44366 (42904–45930) [IntCal20]
Yana1	(8)	SAMEA5684442	P1	31850 (31532–32200) [IntCal20]
A00	(5)	GRC13292545<br>GRC13292546	A00	0
HG02982	(4)	SAMN01761225	A0a1	0
HG02666	(4)	SAMN00779975	A1a	0
HGDP01029	(3)	SAMEA2580841	A1b1a1a1	0
HGDP01406	(3)	SAMEA2580853	A1b1b2b	0
HGDP00931	(3)	SAMEA2580871	B-M181	0
HGDP00478	(3)	SAMEA2580834	B2a	0
HGDP00453	(3)	SAMEA2580803	B2b2	0
HGDP00475	(3)	SAMEA2580818	B2b1a1c	0
HGDP00984	(3)	SAMEA2580836	B2b1a1a	0
HGDP00757	(3)	SAMEA2581095	D1b2a2	0
HGDP01214	(3)	SAMEA2581057	D1a1a	0
HGDP01031	(3)	SAMEA2580848	E2b1a1	0

HGDP01200	(3)	SAMEA2580900	E1a2a1b1	0
HGDP00908	(3)	SAMEA2580889	E1b1a1a1c2c3b	0
HGDP00103	(3)	SAMEA2581152	C2b1c	0
HGDP00213	(3)	SAMEA2581196	G2b1	0
HGDP00127	(3)	SAMEA2581169	I2a2a1a2a2	0
HGDP00057	(3)	SAMEA2581295	J1a2b	0
HGDP00056	(3)	SAMEA2581294	J2a1	0
HGDP00549	(3)	SAMEA2581681	M1	0
HGDP01009	(3)	SAMEA2581745	Q1a2a1a1	0
HGDP00136	(3)	SAMEA2581321	R1a1a1b2	0
HGDP00218	(3)	SAMEA2581199	R2a	0
HGDP01298	(3)	SAMEA2581140	L1a2	0
HGDP01192	(3)	SAMEA2581001	N1	0
HGDP01225	(3)	SAMEA2581048	O2a2b1a1a	0
HGDP01190	(3)	SAMEA2580999	O1b1a1a1a	0

Table S36. Estimate of modern human contamination based on the sequences of the *Denisova 17* Y chromosome.

	Sequences with the modern human allele	Number of sequences overlapping informative sites	Contamination %	95% binomial CI
**All sequences**	57	3250	1.72	(1.28, 2.17)

Table S37. Missense variants detected in the *D17* Y chromosome.

Missense Variant	SNP	Max frequency	Gene	Gene Function	Phenotypes in modern humans associated with other variants in these genes
Y_6932032_G/C	rs776986764	0.001087	*TBL1Y*	Transcription activator	Deafness, Y-Linked 2
Y_9384122_A/C	rs866266594	0.006822	*FAM197Y1*	RNA Gene	Not available
Y_14832620_G/T	rs7067496	0.7452	*USP9Y*	Peptidase	Spermatogenic failure, Y-linked, 2<br>Partial Chromosome Y Deletion
Y_21868167_C/T	rs771050756	0.00001778	*KDM5D*	Histone demethylase	Spermatogenic failure, Y-linked, 2
Y_21905071_C/T	rs1034100630	0.00006879			

Table S38. Sharing of missense variants amongst Neandertal and Denisovan Y chromosomes. The numbers in brackets represent the coverage at these positions.

Variant	Ancestral allele	*Den17*	*Chag2*	*Mez2*	*Spy94a*	*El Sidrón 1253*	*Den4*	*Den8*
Y_6932032_G/C	G	C (24)	C (56)	C (17)	-	-	G (1)	G (4)
Y_9384122_A/C	G	C (7)	C (51)	C (8)	-	-	C (3)	C (3)
Y_14832620_G/T	T	T (18)	-	-	-	T (16)	-	T (5)
Y_21868167_C/T	T	T (11)	T (53)	T (12)	T (1)	T (19)	C (1)	C (6)
Y_21905071_C/T	T	T (27)	T (69)	T (16)	T (1)	T (12)	T (1)	T (5)

Table S39. Relationship (based on genotypes) between the *D17* Y chromosome and the Y chromosomes of *Chagyrskaya 2* and *Mezmaiskaya 2*. Sites used for this analysis are present in all three Neandertals and are derived in Denisova 17 but ancestral in the human reference.

	Number of positions	*Mezmaiskaya 2*	*Chagyrskaya 2*
**All positions**	840	1	0
**Transversions only**	315	0	0

Table S40. Y chromosome relationship (based on sequenced fragments) between *D17*, *Chagyrskaya 2*, *Mezmaiskaya 2* and *Spy 94a*, *El Sidrón 1253*.

		Ancestral allele count	Derived allele count	% matching	95% binomial CI
D17* *(113 diagnostic sites)	***Spy94a***	45	1	2%	(0.0, 6.4)
	El Sidrón 1253	86	0	0%	(0.0, 0.0)
***Mezmaiskaya 2* *(169* diagnostic sites)**	***Spy94a***	41	17	29%	(17.6, 41.0)
	El Sidrón 1253	23	2	8%	(0.0, 18.6)
Chagyrskaya 2* *(106 diagnostic sites)	***Spy94a***	36	0	0%	(0.0, 0.0)
	El Sidrón 1253	7	25	78%	(63, 92)

Table S41. The number of positions used for the TMRCA analyses of the low-coverage Neandertals.

	No human-chimp divergence filter	With human-chimp divergence filter
Spy94a (no coverage filter)	1,927,875	1,055,301
Spy94a (2x min coverage filter)	842,160	465,275
Spy94a (3x min coverage filter)	331,085	182,860
El Sidrón 1253	356,451	197,647

Table S42. Nucleotide substitution rates estimated by bModelTest

Substitution types	Substitution rates from bModelTest
AC	0.547
AG	1.857
AT	0.268
CG	0.925
CT	1.857
GT	0.547

Table S43. Tip date priors for the ancient individuals in the BEAST analysis.

**Individual**	**Initial/Starting Tip Date (years before present)**	**Tip Date Prior (years before present)**
A00_I10871	7,913	7,839 - 8,008
Loschbour	8,025	7,936 - 8,171
Yana1	31,850	31,532 - 32,200
Mezmaiskaya 2	43,449	42,156 - 44,965
Ust'Ishim	44,366	42,904 - 45,930
Chagyrskaya 2	72,000	40,000 - 120,000
D17	120,000	40,000 - 250,000

Table S44. Marginal log-likelihood for the model combinations analysed using the path sampling method in BEAST2. The two best-supported model combinations are shown in bold.

**Clock Model**	**Tree Model**	**Marginal log-likelihood**
Strict	Coalescent Constant Population	-3,203,877.57
**Strict**	**Coalescent Bayesian Skyline**	**-3,203,862.45**
Relaxed Log Normal	Coalescent Constant Population	-3,203,876.85
**Relaxed Log Normal**	**Coalescent Bayesian Skyline**	**-3,203,861.33**

References:

1. S. Brown, *et al.*, The earliest Denisovans and their cultural adaptation. *Nat Ecol Evol* 6, 28–35 (2022).

2. J. Dabney, *et al.*, Complete mitochondrial genome sequence of a Middle Pleistocene cave bear reconstructed from ultrashort DNA fragments. *Proc Natl Acad Sci U S A* 110, 15758–63 (2013).

3. N. Rohland, I. Glocke, A. Aximu-Petri, M. Meyer, Extraction of highly degraded DNA from ancient bones, teeth and sediments for high-throughput sequencing. *Nat Protoc* 13, 2447–2461 (2018).

4. M.-T. Gansauge, *et al.*, Single-stranded DNA library preparation from highly degraded DNA using T4 DNA ligase. *Nucleic Acids Res* 45, e79 (2017).

5. M. T. Gansauge, M. Meyer, Manual and automated preparation of single-stranded DNA libraries for the sequencing of DNA from ancient biological remains and other sources of highly degraded DNA. *Nat Protoc* in press (2020).

6. M. Kircher, S. Sawyer, M. Meyer, Double indexing overcomes inaccuracies in multiplex sequencing on the Illumina platform. *Nucleic Acids Res* 40, e3 (2012).

7. G. Renaud, U. Stenzel, J. Kelso, leeHom: adaptor trimming and merging for Illumina sequencing reads. *Nucleic Acids Res* 42, e141–e141 (2014).

8. K. Prüfer, *et al.*, A high-coverage Neandertal genome from Vindija Cave in Croatia. *Science* 358, 655–658 (2017).

9. H. Li, R. Durbin, Fast and accurate short read alignment with Burrows-Wheeler transform. *Bioinformatics* 25, 1754–1760 (2009).

10. M. Meyer, *et al.*, A high-coverage genome sequence from an archaic Denisovan individual. *Science* 338, 222–6 (2012).

11. Q. Fu, *et al.*, The genetic history of Ice Age Europe. *Nature* 534, 200–205 (2016).

12. GitHub - yanivsw/ancient_dna_cpp_tools. Available at: https://github.com/yanivsw/ancient_dna_cpp_tools [Accessed 4 January 2026].

13. H. Li, *et al.*, The Sequence Alignment/Map format and SAMtools. *Bioinformatics* 25, 2078 (2009).

14. A. McKenna, *et al.*, The genome analysis toolkit: A MapReduce framework for analyzing next-generation DNA sequencing data. *Genome Res* 20 (2010).

15. K. Prüfer, SNPAD: An ancient DNA genotype caller. *Bioinformatics* 34 (2018).

16. T. Derrien, *et al.*, Fast Computation and Applications of Genome Mappability. *PLoS One* 7, e30377 (2012).

17. G. Benson, Tandem repeats finder: A program to analyze DNA sequences. *Nucleic Acids Res* 27 (1999).

18. K. Prüfer, *et al.*, The complete genome sequence of a Neanderthal from the Altai Mountains. *Nature* 505, 43–49 (2014).

19. M. Meyer, *et al.*, Nuclear DNA sequences from the Middle Pleistocene Sima de los Huesos hominins. *Nature* 531, 504–507 (2016).

20. K. J. Karczewski, *et al.*, The mutational constraint spectrum quantified from variation in 141,456 humans. *Nature* 581, 434–443 (2020).

21. S. Sawyer, J. Krause, K. Guschanski, V. Savolainen, S. Pääbo, Temporal Patterns of Nucleotide Misincorporations and DNA Fragmentation in Ancient DNA. *PLoS One* 7, e34131 (2012).

22. A. W. Briggs, *et al.*, Patterns of damage in genomic DNA sequences from a Neandertal. *Proc Natl Acad Sci U S A* 104, 14616–14621 (2007).

23. S. Peyrégne, B. M. Peter, AuthentiCT: A model of ancient DNA damage to estimate the proportion of present-day DNA contamination. *Genome Biol* 21 (2020).

24. GitHub - cesaredef/cecast. Available at: https://github.com/cesaredef/cecast [Accessed 21 August 2025].

25. F. Mafessoni, *et al.*, A high-coverage Neandertal genome from Chagyrskaya Cave. *Proc Natl Acad Sci U S A* 117, 15132–15136 (2020).

26. Q. Fu, *et al.*, Genome sequence of a 45,000-year-old modern human from western Siberia. *Nature* 514, 445–449 (2014).

27. I. Lazaridis, *et al.*, Ancient human genomes suggest three ancestral populations for present-day Europeans. *Nature* 513, 409–413 (2014).

28. D. L. Altshuler, *et al.*, A map of human genome variation from population-scale sequencing. *Nature* 467, 1061–1073 (2010).

29. P. Awadalla, *et al.*, Direct Measure of the De Novo Mutation Rate in Autism and Schizophrenia Cohorts. *Am J Hum Genet* 87, 316 (2010).

30. J. C. Roach, *et al.*, Analysis of Genetic Inheritance in a Family Quartet by Whole Genome Sequencing. *Science* 328, 636 (2010).

31. A. Scally, R. Durbin, Revising the human mutation rate: implications for understanding human evolution. *Nat Rev Genet* 13, 745–753 (2012).

32. K. E. Langergraber, *et al.*, Generation times in wild chimpanzees and gorillas suggest earlier divergence times in great ape and human evolution. *Proc Natl Acad Sci U S A* 109, 15716–15721 (2012).

33. F. M. T. A. Busing, E. Meijer, R. Van Der Leeden, Delete-m Jackknife for Unequal m. *Stat Comput* 9, 3–8 (1999).

34. N. Patterson, *et al.*, Ancient admixture in human history. *Genetics* 192, 1065–93 (2012).

35. GitHub - DReichLab/AdmixTools: Tools test whether admixture occurred and more. Available at: https://github.com/DReichLab/AdmixTools [Accessed 20 January 2026].

36. H. Ringbauer, G. Coop, N. H. Barton, Inferring Recent Demography from Isolation by Distance of Long Shared Sequence Blocks. *Genetics* 205, 1335–1351 (2017).

37. D. Popli, S. Peyrégne, B. M. Peter, KIN: a method to infer relatedness from low-coverage ancient DNA. *Genome Biol* 24, 1–22 (2023).

38. J. Kelleher, A. M. Etheridge, G. McVean, Efficient Coalescent Simulation and Genealogical Analysis for Large Sample Sizes. *PLoS Comput Biol* 12, e1004842 (2016).

39. M. Caballero, *et al.*, Crossover interference and sex-specific genetic maps shape identical by descent sharing in close relatives. *PLoS Genet* 15, e1007979 (2019).

40. A. G. Hinch, *et al.*, The landscape of recombination in African Americans. *Nature* 476, 170 (2011).

41. A. Kong, *et al.*, Fine-scale recombination rate differences between sexes, populations and individuals. *Nature* 467, 1099–1103 (2010).

42. H. Li, R. Durbin, Inference of human population history from individual whole-genome sequences. *Nature* 475, 493–496 (2011).

43. A. P. Sümer, *et al.*, Earliest modern human genomes constrain timing of Neanderthal admixture. *Nature 2024 638:8051* 638, 711–717 (2024).

44. P. R. Staab, S. Zhu, D. Metzler, G. Lunter, scrm: efficiently simulating long sequences using the approximated coalescent with recombination. *Bioinformatics* 31, 1680 (2015).

45. F. Pouyet, S. Aeschbacher, A. Thiéry, L. Excoffier, Background selection and biased gene conversion affect more than 95% of the human genome and bias demographic inferences. *Elife* 7 (2018).

46. P. Johri, *et al.*, The Impact of Purifying and Background Selection on the Inference of Population History: Problems and Prospects. *Mol Biol Evol* 38, 2986–3003 (2021).

47. G. McVicker, D. Gordon, C. Davis, P. Green, Widespread genomic signatures of natural selection in hominid evolution. *PLoS Genet* 5 (2009).

48. M. Hajdinjak, *et al.*, Reconstructing the genetic history of late Neanderthals. *Nature* 555, 652–656 (2018).

49. J. Kamm, J. Terhorst, R. Durbin, Y. S. Song, Efficiently inferring the demographic history of many populations with allele count data. *J Am Stat Assoc* 115, 1472–1487 (2020).

50. J. C. Nash, On Best Practice Optimization Methods in R. *J Stat Softw* 60, 1–14 (2014).

51. O. Mazet, W. Rodríguez, L. Chikhi, Demographic inference using genetic data from a single individual: Separating population size variation from population structure. *Theor Popul Biol* 104, 46–58 (2015).

52. T. Cousins, A. Scally, R. Durbin, A structured coalescent model reveals deep ancestral structure shared by all modern humans. *Nature Genetics 2025 57:4* 57, 856–864 (2025).

53. S. WRIGHT, The genetical structure of populations. *Ann Eugen* 15, 323–354 (1951).

54. R. R. Hudson, M. Slatkin, W. P. Maddison, Estimation of Levels of Gene Flow from DNA Sequence Data. *Genetics* 132, 583 (1992).

55. D. Ortega-Del Vecchyo, M. Slatkin, FST between archaic and present-day samples. *Heredity (Edinb)* 122, 711 (2018).

56. A. Auton, *et al.*, A global reference for human genetic variation. *Nature* 526, 68–74 (2015).

57. E. M. Scott, *et al.*, Characterization of greater middle eastern genetic variation for enhanced disease gene discovery. *Nat Genet* 48, 1071–1079 (2016).

58. L. L. Cavalli-Sforza, P. Menozzi, A. Piazza, The History and Geography of Human Gene. (Abridged paperback edition). *J R Anthropol Inst* 2, 413 (1996).

59. V. Slon, *et al.*, The genome of the offspring of a Neanderthal mother and a Denisovan father. *Nature* 561, 113–116 (2018).

60. B. M. Peter, 100,000 years of gene flow between Neandertals and Denisovans in the Altai mountains. *bioRxiv* 2020.03.13.990523 (2020). https://doi.org/10.1101/2020.03.13.990523.

61. S. Peyrégne, *et al.*, A high-coverage genome from a 200,000-year-old Denisovan. *bioRxiv* 2025.10.20.683404 (2025). https://doi.org/10.1101/2025.10.20.683404.

62. A. G. Hinch, *et al.*, The landscape of recombination in African Americans. *Nature* 476, 170–175 (2011).

63. L. N. M. Iasi, *et al.*, Neanderthal ancestry through time: Insights from genomes of ancient and present-day humans. *Science* 386, eadq3010 (2024).

64. A. L. Hughes, M. Yeager, Natural selection at major histocompatibility complex loci of vertebrates. *Annu Rev Genet* 32, 415–435 (1998).

65. D. Massilani, *et al.*, Denisovan ancestry and population history of early East Asians. *Science* 370, 579–583 (2020).

66. F. A. Villanea, *et al.*, The MUC19 gene: An evolutionary history of recurrent introgression and natural selection. *Science (1979)* 389, eadl0882 (2025).

67. L. Skov, *et al.*, Genetic insights into the social organization of Neanderthals. *Nature* 610 (2022).

68. P. K. Albers, G. McVean, Dating genomic variants and shared ancestry in population-scale sequencing data. *PLoS Biol* 18 (2020).

69. S. Peyrégne, J. Kelso, B. M. Peter, S. Pääbo, The evolutionary history of human spindle genes includes back-and-forth gene flow with Neandertals. *Elife* 11 (2022).

70. M. Kuhlwilm, *et al.*, Ancient gene flow from early modern humans into Eastern Neanderthals. *Nature 2016 530:7591* 530, 429–433 (2016).

71. M. Petr, *et al.*, The evolutionary history of Neanderthal and Denisovan Y chromosomes. *Science (1979)* 369 (2020).

72. M. J. Hubisz, A. L. Williams, A. Siepel, Mapping gene flow between ancient hominins through demography-aware inference of the ancestral recombination graph. *PLoS Genet* 16 (2020).

73. L. Skov, *et al.*, Detecting archaic introgression using an unadmixed outgroup. *PLoS Genet* 14 (2018).

74. S. Mallick, *et al.*, The Simons Genome Diversity Project: 300 genomes from 142 diverse populations. *Nature* 538, 201–206 (2016).

75. A. Bergström, *et al.*, Insights into human genetic variation and population history from 929 diverse genomes. *Science* 367 (2020).

76. M. Byrska-Bishop, *et al.*, High-coverage whole-genome sequencing of the expanded 1000 Genomes Project cohort including 602 trios. *Cell* 185, 3426-3440.e19 (2022).

77. M. Karmin, *et al.*, A recent bottleneck of Y chromosome diversity coincides with a global change in culture. *Genome Res* 25, 459 (2015).

78. M. Sikora, *et al.*, The population history of northeastern Siberia since the Pleistocene. *Nature* 1 (2019). https://doi.org/10.1038/s41586-019-1279-z.

79. M. Lipson, *et al.*, Ancient West African foragers in the context of African population history. *Nature* 577, 665–670 (2020).

80. F. L. Mendez, G. D. Poznik, S. Castellano, C. D. Bustamante, The Divergence of Neandertal and Modern Human Y Chromosomes. *Am J Hum Genet* 98, 728 (2016).

81. Picard Tools - By Broad Institute. Available at: https://broadinstitute.github.io/picard/?utm_source=chatgpt.com [Accessed 12 October 2025].

82. H. Li, R. Durbin, Fast and accurate short read alignment with Burrows-Wheeler transform. *Bioinformatics* 25, 1754–60 (2009).

83. H. Li, R. Durbin, Fast and accurate long-read alignment with Burrows-Wheeler transform. *Bioinformatics* 26, 589–595 (2010).

84. W. McLaren, *et al.*, The Ensembl Variant Effect Predictor. *Genome Biol* 17 (2016).

85. Y. Swiel, J. Kelso, S. Peyrégne, Resolving the source of branch length variation in the Y chromosome phylogeny. *Genome Biol* 26 (2025).

86. R. Bouckaert, *et al.*, BEAST 2.5: An advanced software platform for Bayesian evolutionary analysis. *PLoS Comput Biol* 15 (2019).

87. R. R. Bouckaert, A. J. Drummond, bModelTest: Bayesian phylogenetic site model averaging and model comparison. *BMC Evol Biol* 17, 1–11 (2017).

88. P. J. Reimer, *et al.*, The IntCal20 Northern Hemisphere Radiocarbon Age Calibration Curve (0–55 cal kBP). *Radiocarbon* 62, 725–757 (2020).
